# Metallothionein‐Inspired Asymmetric Heteroatom Doping of Single‐Atom Nanozymes for Multi‐Enzyme Biocatalysis

**DOI:** 10.1002/advs.202517502

**Published:** 2025-11-03

**Authors:** Kaijuan Chen, Qianfan Chen, Bernt Johannessen, Chun‐Ho Lin, Long Hu, Kang Liang, Jieying Liang

**Affiliations:** ^1^ School of Chemical Engineering Australian Centre for NanoMedicine The University of New South Wales Sydney NSW 2052 Australia; ^2^ Graduate School of Biomedical Engineering The University of New South Wales Sydney NSW 2052 Australia; ^3^ Australian Synchrotron ANSTO Clayton Victoria 3168 Australia; ^4^ School of Materials Science and Engineering University of New South Wales (UNSW) Sydney NSW 2052 Australia

**Keywords:** biocatalysis, biomineralization, metal‐organic frameworks, nanozymes, single atom catalysts

## Abstract

Single‐atom catalysts (SACs) exhibit enzyme‐mimicking activity but are often limited by single‐enzyme–like functions and modest catalytic efficiency. Here, a metallothionein‐inspired heteroatom doping strategy is reported to construct asymmetric Fe single‐atom catalysts (FeN_3_S). Fe^3^⁺ is coordinated with cysteine via strong mercaptide bond formation, followed by zeolitic imidazolate framework‐8 (ZIF‐8) biomineralization and pyrolysis. The FeN_3_S catalyst displays markedly enhanced multi‐enzyme activities—including NADH oxidase‐, oxidase‐, peroxidase‐, and catalase‐like activities—with 1.35–4.60‐fold improvements compared to sulfur (S)‐free analogues. This high multi‐enzyme efficiency arises from i) atomically dispersed Fe from biomineralization; ii) the large surface area and pore volume retained from the original metal–organic framework, and iii) the S‐doping achieved through the strong mercaptide coordination between Fe and S. The S doping not only tunes electronic structure of Fe single atom to reduce activation barriers and enhances substrate interaction, but also facilitates charge transfer. As a result, FeN_3_S induces ≈90% tumor cell suppression within one day through reactive oxygen species generation and disruption of the NADH/NAD⁺ balance, highlighting its strong potential for cancer therapy. This work provides a bioinspired strategy for advancing SACs toward multifunctional biocatalysis and biomedical applications.

## Graphical abstract

A metallothionein‐inspired heteroatom doping strategy is reported to construct asymmetric Fe single‐atom catalysts, which display markedly enhanced multi‐enzyme activities—including NADH oxidase‐, oxidase‐, peroxidase‐, and catalase‐like activities. This high multi‐enzyme efficiency arises from atomically dispersed Fe from biomineralization, the large surface area and pore volume retained from the original metal–organic framework, and regulated asymmetric coordination of F single atom.

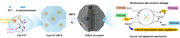

## 1 Introduction

Natural enzymes are widely used in biocatalysis, medicine, and industry due to their remarkable catalytic efficiency and substrate specificity.^[^ ^1^, ^2^, ^3^ ^]^ However, their practical applications are often limited by poor stability, sensitivity to temperature and pH, low tolerance to organic solvents, and high production costs.^[^ ^4^, ^5^ ^]^ To address these limitations, nanozymes—nanomaterials with enzyme‐like activity—have emerged.^[^ ^6^, ^7^ ^]^ Compared with natural enzymes, nanozymes offer advantages such as high stability, facile design, and low manufacturing cost.^[^ ^8^, ^9^, ^10^, ^11^ ^]^ However, their catalytic activity and selectivity are generally lower than those of natural enzymes, and their potential cytotoxicity remains a concern.^[^ ^12^, ^13^, ^14^, ^15^ ^]^ As one of the emerging nanozymes, single‐atom catalysts (SACs) have recently attracted significant attention, demonstrating excellent structural stability, high catalytic activity, and selectivity.^[^ ^16^ ^]^ For example, the developed SACs have been demonstrated to mimic the activities of peroxidase (POD),^[^ ^17^ ^]^ oxidase (OXD),^[^ ^18^ ^]^ catalase (CAT),^[^ ^19^ ^]^ laccase,^[^ ^20^ ^]^ and superoxide dismutase.^[^ ^21^ ^]^ These advantages arise from atomically dispersed active sites, tunable coordination environments, and maximal metal utilization.^[^ ^22^ ^]^ Consequently, SACs show broad application prospects in biosensing, disease therapy, environmental remediation, and energy conversion,^[^ ^23^, ^24^, ^25^, ^26^ ^]^ establishing them as a rapidly growing research hotspot in catalysis.

However, there remains significant room for improving the performance of SACs. Various strategies have been explored, including constructing porous structures to enhance substrate mass transfer,^[^ ^27^, ^28^ ^]^ introducing heteroatoms (such as N,^[^ ^29^ ^]^ S,^[^ ^30^, ^31^ ^]^ P,^[^ ^7^ ^]^ B^[^ ^32^ ^]^) to modulate the electronic structure of metal centers, and improving the stability of active sites through optimized carriers and metal–carrier interactions.^[^ ^23^, ^33^, ^34^ ^]^ While these approaches have achieved notable improvements in catalytic activity, they also present challenges, such as reduced structural stability, disruption of active site homogeneity, agglomeration at high metal loadings,^[^ ^35^, ^36^, ^37^ ^]^ and restriction to single enzyme‐like activity.^[^ ^29^ ^]^ In particular, previous heteroatom‐doping strategies have mainly relied on heteroatom‐containing polymers^[^ ^7^, ^38^, ^39^ ^]^ or small molecules^[^ ^32^ ^]^ that lack specific anchoring sites for metal ions, and depending on host–guest self‐adjustment during pyrolysis,^[^ ^40^ ^]^ This often leads to poor coordination between metal and nonmetal atoms or excessive heteroatom loading without deliberate design. Moreover, although recent comprehensive reviews have highlighted both the remarkable potential and ongoing challenges in developing selective single‐atom nanozymes,^[^ ^41^, ^42^, ^43^, ^44^, ^45^ ^]^ the single enzyme‐like activity of most SACs limits their effectiveness in complex environmental and biological processes that often require multiple catalytic functions.^[^ ^46^ ^]^ Recently, Ir–N_5_ SAC was developed, demonstrating multi‐enzyme activities, including CAT‐, OXD‐, POD‐, and NADH oxidase (NOX)‐like activities.^[^ ^47^ ^]^ Nevertheless, the catalytic efficiency of Ir–N_5_ SAC remains limited, and the use of cerulenin was necessary to achieve effective cancer therapy.^[^ ^47^ ^]^ Therefore, the development of SACs with precisely designed heteroatom doping capable of mimicking multiple enzyme activities while maintaining high catalytic efficiency remains a critical challenge in this field.

**Scheme 1:**
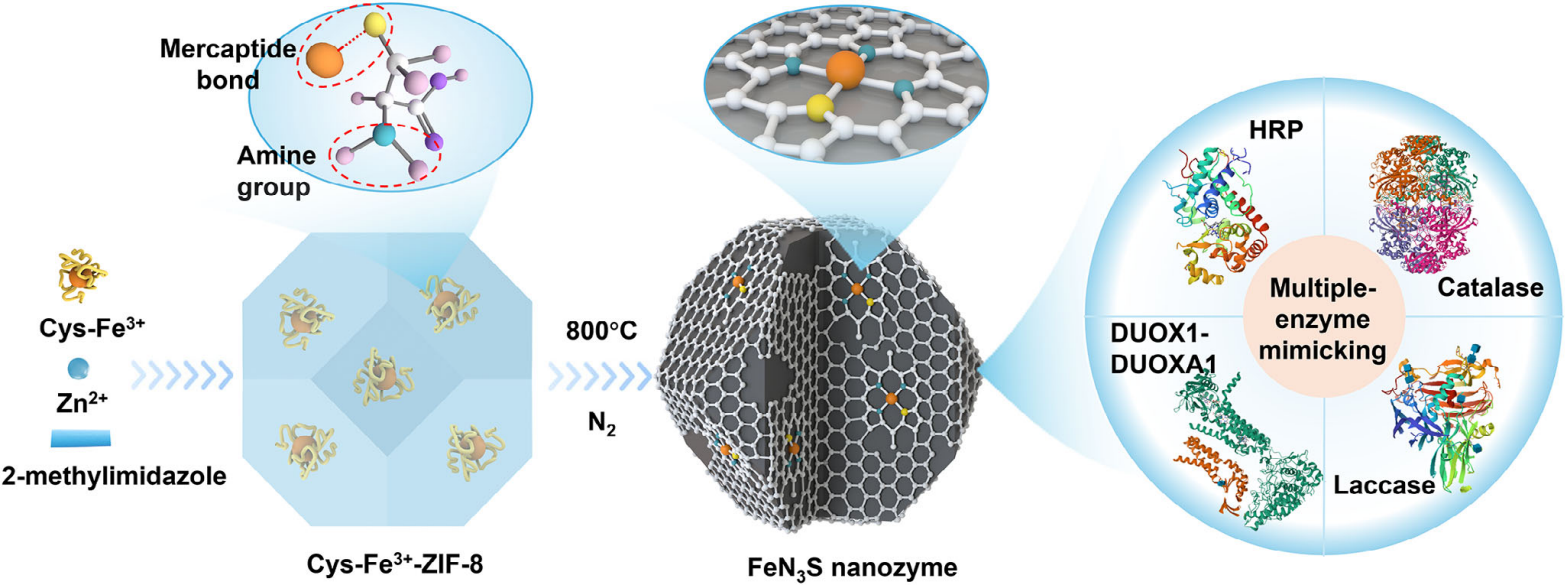
Illustration of the preparation process of FeN_3_S nanozyme for multiple‐enzyme mimicking. Cys was coordinated with Fe^3^⁺ through strong mercaptide bonds, while the amine group in Cys promoted the biomineralization of MOFs. After pyrolysis, the obtained FeN_3_S nanozyme exhibits multi‐enzyme mimetic activities, including NOX, OXD‐, POD‐, and CAT‐like activities.

To develop highly efficient Fe‐SACs with multi‐enzyme activity for effective biocatalysis, a metallothionein‐inspired heteroatom doping strategy was employed to engineer asymmetric Fe SACs (**Scheme** **1**). This approach was inspired by the metal cation accumulation mechanism of metallothionein.^[^ ^48^ ^]^ Compared with other S‐containing ligands (e.g., 1,2‐ethanedithiol, 1‐butanethiol, and thioglycolic acid), L‐cysteine methyl ester hydrochloride (Cys) not only coordinated with Fe^3^⁺ ions through strong mercaptide bonds, but its amine group also promoted the assembly of prenucleation clusters and facilitated the subsequent formation of MOFs around the Cys molecules.^[^ ^49^ ^]^ This strategy enables highly dispersed and effective immobilization of Cys‐Fe^3^⁺ within the MOF framework. Following pyrolysis, asymmetric FeN_3_S SACs were obtained, exhibiting remarkable multi‐enzyme mimetic activities, including NOX, OXD‐, POD‐, and CAT‐like activities (Scheme 1), with 1.35–4.60‐fold higher activity compared to FeN_3_ SACs without S doping. The highly efficient multi‐enzyme activity of FeN_3_S can be attributed to i) the highly dispersed single atoms originating from the biomineralization; ii) the large surface area and pore volume retained from the original MOF; and iii) the S‐doping achieved through the strong mercaptide coordination between Fe and S. The S doping not only tunes electronic structure of Fe single atom to reduce activation barriers and enhances substrate interaction, but also facilitates charge transfer. Finally, FeN_3_S with multi‐enzyme‐like activity was applied to suppress tumor cell growth, achieving an ≈90% cell death rate within one day. This study provides a promising strategy for developing highly efficient, multi‐enzyme‐like SACs for complex environmental and biomedical applications.

## 2 Results and Discussion

### 2.1 Synthesis and Structural Characterization of the Fe‐SACs

Zeolitic imidazolate framework‐8 (ZIF‐8), rich in nitrogen and carbon and featuring abundant micropores, has been widely employed for the construction of Fe‐SACs, as its structure effectively hosts Fe–Nx active sites.^[^ ^7^, ^29^ ^]^ Cys, an amino acid, contains a thiolate group that serves as the primary binding site for metal ions, while the histidyl and aspartyl side chains contribute to the stability of the resulting complexes.^[^ ^50^ ^]^ Compared to N, S has a lower electronegativity, making it a highly effective nonmetal dopant.^[^ ^40^ ^]^ To introduce S atoms into the Fe SAC structure, Cys was first mixed with Fe^3+^ ions. A distinct blue color developed upon introducing Fe^3^⁺ solution into Cys solution (Figure S1, Supporting Information), attributable to the [Fe–Cys]⁺ complex, which shows an absorption maximum at 614 nm (ε = 1030 dm^3^mol^−1^cm^−1^).^[^ ^51^ ^]^ The subsequent addition of 2‐methylimidazole (HmIm) and Zn^2^⁺ did not immediately alter the [Fe–Cys]⁺ complex (Figure S1, Supporting Information). Although this [Fe–Cys]⁺ complex is transient, our previous work and other groups revealed that MOFs can rapidly lock such short‐lived biomolecular conformations through rapid nucleation around biomolecules,^[^ ^52^, ^53^ ^]^ with the amine group of Cys facilitating MOF biomineralization.^[^ ^49^, ^54^, ^55^ ^]^ The interaction between Fe^3+^ and Cys was confirmed by Fourier transform infrared (FTIR) spectra (Figure S2, Supporting Information), where the peak at 2550 cm^−1^, corresponding to the S–H stretching vibration of Cys, disappeared after self‐assembly with Fe^3+^, indicating coordination via strong mercaptide bonds.^[^ ^56^ ^]^ The resulting biomineralized MOF was then pyrolyzed at 800 °C under a nitrogen atmosphere to yield SAC, denoted FeN_3_S. Controlled samples lacking Fe^3^⁺, Cys, or both were also prepared and denoted as SNC, FeN_3_, and NC, respectively. For comparison, the higher pyrolysis temperature of 900 °C was also examined, which are designated FeN_3_S‐900, SNC‐900, FeN_3_‐900, and NC‐900, while the corresponding unpyrolyzed MOF samples were denoted Cys‐ZIF‐8, Fe^3+^‐ZIF‐8, and Fe^3+^‐Cys‐ZIF‐8.

**Figure 1:**
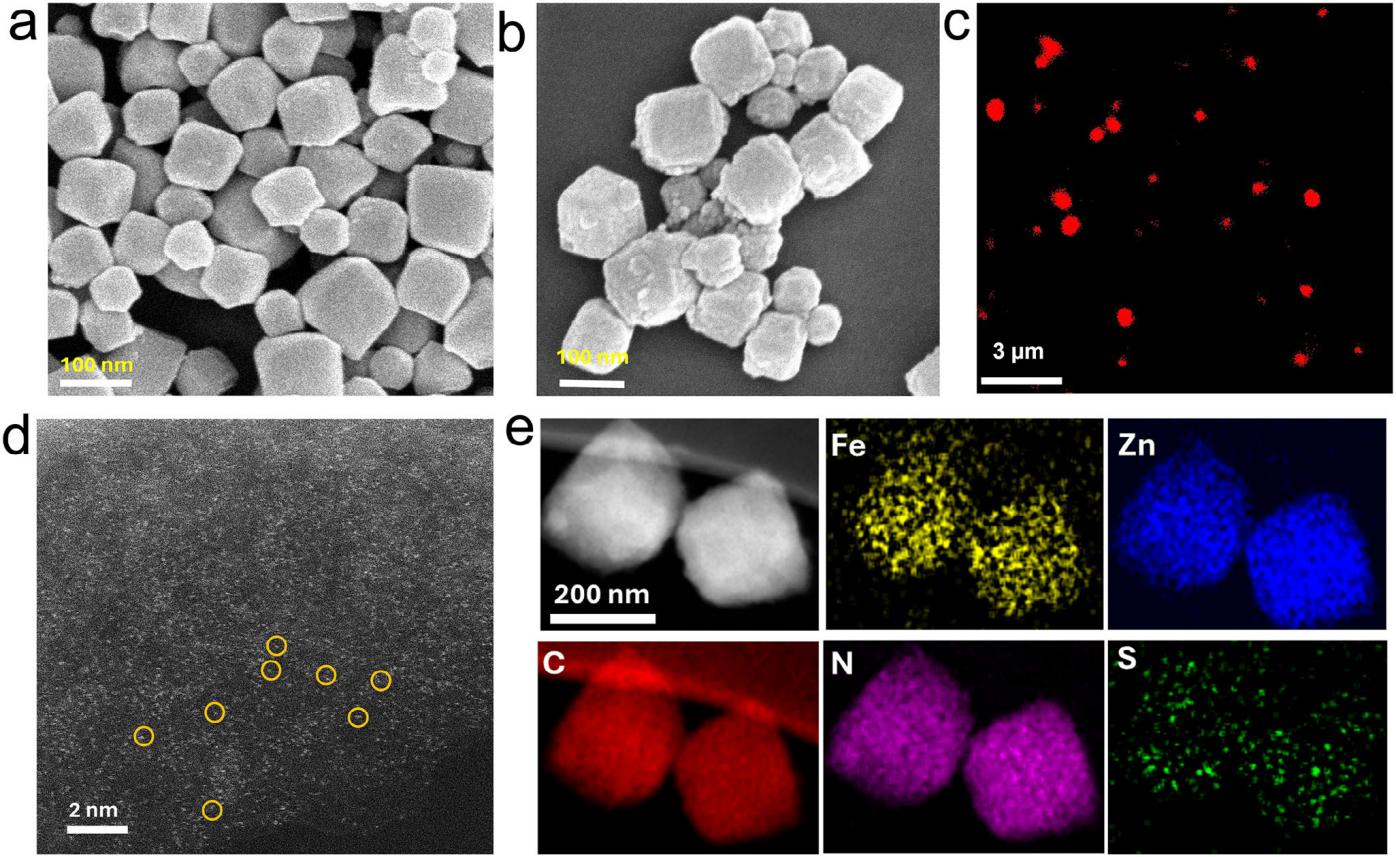
Structural characterization of Fe‐SAC. SEM images of a) Cys–Fe^3^⁺–ZIF and b) FeN_3_S, c) CLSM image of Cys–Fe^3^⁺–ZIF, NHS 633 easter was used to labeled Cys. d) Aberration‐corrected HAADF‐STEM image of FeN_3_S, showing atomically dispersed metal single‐atom sites as bright dots (yellow circles indicate single atoms). e) HAADF‐STEM image and corresponding EDS mapping of FeN_3_S, showing uniform distributions of Fe, Zn, C, N, and S.

Scanning electron microscopy (SEM) images revealed that all unpyrolyzed MOF samples displayed a uniform rhombic dodecahedral morphology^[^ ^57^ ^]^ with an average particle diameter ranging from 75 to 95 nm (Figure S3, Supporting Information). In contrast to Fe^3+^‐Cys‐ZIF‐8, which has a smooth surface, FeN_3_S displays a rough surface morphology due to high‐temperature pyrolysis (**Figure** **1**a,b). Cys was labeled with Atto 633 NHS ester. Confocal laser scanning microscopy (CLSM) images show that Cys (red) is homogeneously distributed within the ZIF‐8 nanocrystals (Figure 1c), indicating that the Fe–Cys complex is encapsulated inside the ZIF‐8 framework rather than attached to its external surface. The powder X‐ray diffraction (PXRD) patterns of Cys‐ZIF‐8, Fe^3+^ZIF‐8, and Fe^3+^‐Cys‐ZIF‐8 exhibited the same characteristic peaks as those of pure ZIF‐8, confirming the successful synthesis and preservation of the crystalline structure after encapsulation of Fe^3+^ and Cys (Figure S4, Supporting Information). Moreover, FeN_3_S, SNC, FeN_3,_ and NC, all samples exhibited consistent morphology and particle size (Figure S5, Supporting Information), indicating the well preservation of structure after pyrolysis treatment. However, the average particle size of SEM images of FeN_3_‐900 and FeN_3_S‐900 decreased slightly (Figure S6, Supporting Information), which may be attributed to structural shrinkage resulting from the decomposition of organic ligands and Zn volatilization during higher temperature pyrolysis.^[^ ^58^ ^]^ These results are also in agreement with TEM images (Figure S7, Supporting Information). After pyrolysis at 800 and 900 °C, the characteristic peaks of ZIF‐8 in PXRD patterns disappeared, and two broad reflections corresponding to (002) and (101) planes emerged at ≈2θ = 24° and 43°, indicating the formation of amorphous graphitic carbon structures (Figure S8, Supporting Information). The Raman spectra of pyrolyzed samples further confirmed the formation of graphitic carbon, as only two characteristic peaks corresponding to the D and G bands of carbon materials were observed (Figure S9, Supporting Information). The I_D_/I_G_ values of NC, SNC, FeN_3,_ and FeN_3_S were estimated as 1.057, 1.035, 1.028, and 1.040, with similar values observed for the samples pyrolyzed at 900 °C. These results imply abundant structural imperfections and a significant degree of graphitization,^[^ ^29^ ^]^ consistent with the PXRD patterns (Figure S8, Supporting Information). Moreover, it is observed that the I_D_/I_G_ values all decrease after the introduction of S, Fe, or both of them, reflecting that they facilitate MOF graphitization. This enhanced graphitic structure might be beneficial for accelerating interfacial electron transfer between the catalyst and reactants, thereby improving the potential catalytic activity of the SACs.^[^ ^34^ ^]^

The X‐ray photoelectron spectroscopy (XPS) measurements revealed that all samples contained C, O, N, and Zn elements (Table S1, Supporting Information). In the N 1s XPS spectra of ZIF‐8, Cys‐ZIF‐8, Fe^3^⁺‐ZIF‐8, and Fe^3^⁺‐Cys‐ZIF‐8, a major peak at 398.5 eV is clearly observed, corresponding to the C═N bond in HmIm (Figure S10, Supporting Information). After pyrolysis, three main peaks at 398.4, 400.5, and 401.2 eV, assigned to pyridinic N, pyrrolic N, and graphitic N appeared, respectively^[^ ^29^ ^]^ (Figures S11 and S12, Supporting Information). Pyridinic N serves as a primary anchoring site for single metal atoms, while pyrrolic N and graphitic N modulate the electronic structure of the carbon materials, thereby enhancing the catalytic activity of the SACs,^[^ ^59^, ^60^, ^61^ ^]^ The presence of single metal (Zn and Fe) atoms was confirmed by high‐angle aberration‐corrected annular dark‐field scanning transmission electron microscopy (HAADF‐STEM), presented as the bright spots marked with yellow circles in the images (Figure 1d and Figure S13, Supporting Information), indicating highly dispersed single atoms derived from MOF biomineralization^[^ ^55^ ^]^ followed by pyrolysis. Meanwhile, the energy‐dispersive spectrometry (EDS) exhibited uniform distribution of Fe, Zn, C, N, and S elements in FeN_3_S and FeN_3_S‐900 (Figure 1e; Figures S14 and S15, Supporting Information), confirming the incorporation of Fe atoms and S doping. This is also consistent with the electron energy‐loss spectrum (EELS) mappings (Figure S16a, Supporting Information), Fe shows a pronounced Fe L_3_ edge at ≈708 eV (Figure S16b,c, Supporting Information), providing further evidence of the Fe in FeN_3_S. However, inductively coupled plasma (ICP) results show that the Zn content is over ten times higher than that of Fe (Table S2, Supporting Information), making it challenging to clearly distinguish Fe single atoms from Zn in Figure 1d. Importantly, no aggregation of either Zn or Fe is observed. EDS and EELS mapping (Figures S14 and S16, Supporting Information) confirm that Fe exists as isolated single atoms rather than aggregated clusters.

### 2.2 Atomic Structure Analysis of Fe‐SACs by X‐ray Absorption Fine Structure Spectroscopy

**Figure 2:**
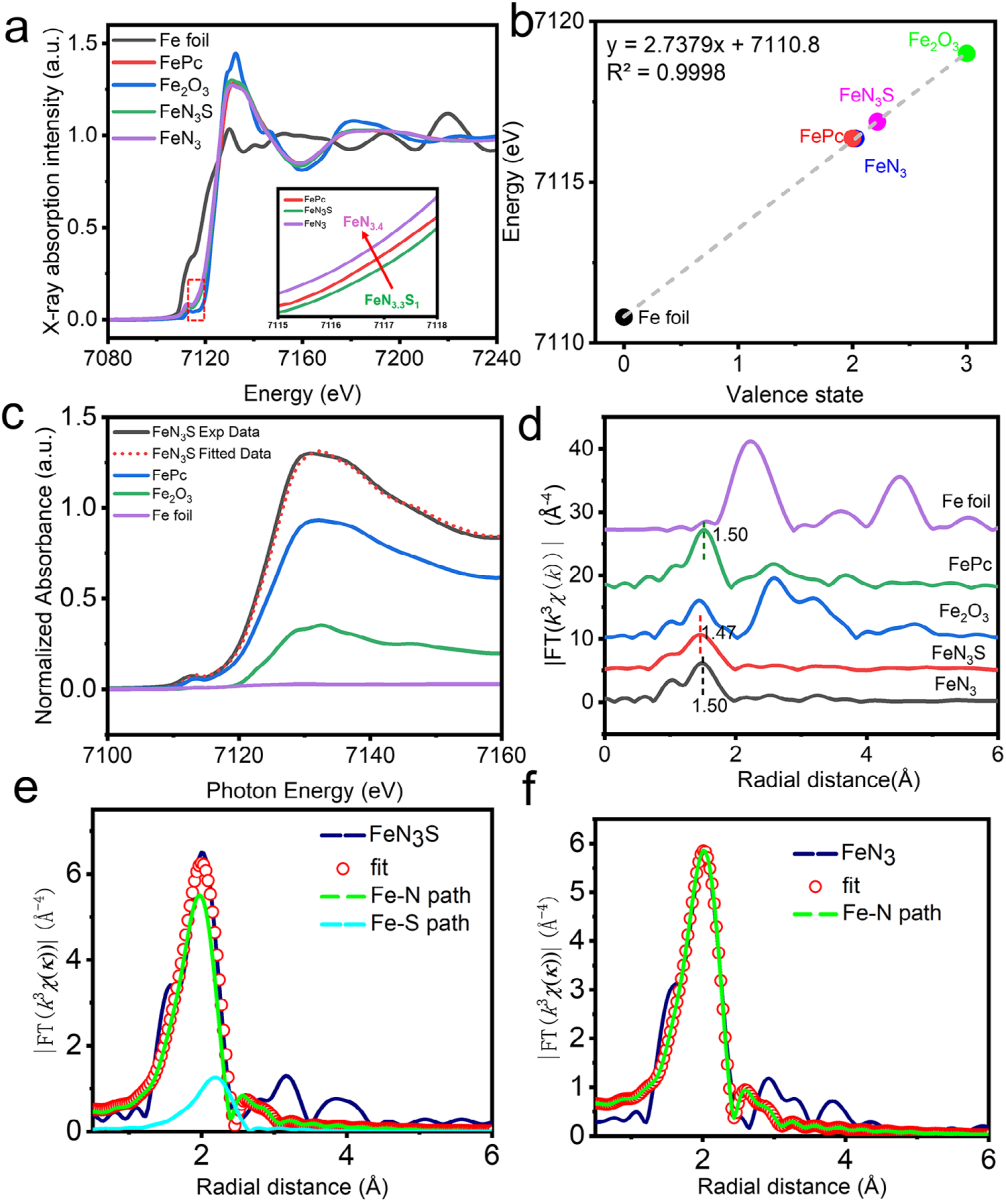
X‐ray absorption spectra of Fe‐SACs. a) Fe K‐edge XANES spectra of Fe foil, Fe_2_O_3_, FePc, FeN_3_S, and FeN_3_. The inset shows the enlarged energy of FeN_3_S and FeN_3_ range from 7116 to 7118 eV. b) The position of the near‐edge as a function of the valence state of Fe in different samples. c) Linear combination fitting of the XANES data of FeN_3_S collected at the Fe K‐edge. d) Fe K‐edge FT‐EXAFS spectra of Fe foil, Fe_2_O_3_, FePc, FeN_3_S, and FeN_3_. e,f) Phase‐corrected Fe K‐edge EXAFS fitting results in R‐space for e) FeN_3_S and f) FeN_3_.

The chemical state and coordination environment of the Fe‐SACs were examined using Fe K‐edge X‐ray absorption spectroscopy (XAS). In the Fe K‐edge XANES spectra, the near‐edge adsorption energy of the Fe K‐edge in the FeN_3_S is located between to that of FePc and Fe_2_O_3_, which manifests the dominant valence state of between +2 and +3. Specifically, the near‐edge adsorption energy of Fe decreases from FeN_3_S to FeN_3_, whose definite oxidation states could be estimated by comparison with Fe foil, FePc, and Fe_2_O_3_ references (**Figure** **2**a). After enlarging the energy range from 7116 to 7118 eV, a slight shift toward lower energy from FeN_3_S to FePc and to FeN_3_ was observed (Figure 2a, inset), suggesting a slightly decreased oxidation state of Fe.^[^ ^62^, ^63^ ^]^ The oxidation states are +2.22 in FeN_3_S and +2.03 in FeN_3_ (Figure 2b). The linear combination fitting of the Fe K‐edge XANES spectra demonstrates the calculated percentages of the redox states suggest the predominance of Fe(II) (≈72.6%), with smaller contributions from Fe (0) (≈2.9%) and Fe(III) (≈24.5%) for FeN_3_S (Figure 2c; Figure S17, Supporting Information). The reduced chi‐square value (0.0004348) indicates a robust fit, further validating the results (Table S3, Supporting Information).

The formation of asymmetric coordination configurations is confirmed by the Fourier‐transform (FT) of Fe K‐edge EXAFS spectra (Figure 2d). The position of the main peaks corresponding to Fe─N scattering paths shifts slightly and negatively from FeN_3_ to FeN_3_S, which is in accordance with both the average Fe─N bond length by the quantitative EXAFS curve‐fittings. FeN_3_S exhibits a shorter average Fe─N distance (1.997 vs 2.008) (Table S4, Supporting Information). These results demonstrate the presence of local asymmetric configurations upon doping of heteroatoms.^[^ ^64^ ^]^ The quantitative EXAFS curve‐fitting analysis was conducted using least‐squares FT‐EXAFS fitting to investigate the coordination structure (Figure 2e,f; Figures S18 and S19, Supporting Information). The best fitting results for FeN_3_S and FeN_3_ indicate Fe─N with coordination numbers of 3.3 and 3.4 at 2.008 and 1.997 Å, respectively, along with Fe─S coordination number of 1.1 at 2.185 Å for FeN_3_S (Figure 2e,f and Table S4, Supporting Information), consisting with the previous report.^[^ ^65^ ^]^ Hence, the most likely coordination configuration of Fe single atoms is FeN_3_S with S doping and FeN_3_ without S doping (Figure 2e,f). Additionally, experimental 2D wavelet transform extended X‐ray absorption fine structure (2D WT EXAFS) plots also further convince the existence and length of Fe─N bonds (Figures S20 and S21, Supporting Information). In contrast to the Fe foil standard, the Fe─Fe scattering path at ≈2.15 Å disappeared in both curves of FeN_3_S and FeN_3_, suggesting neligible iron aggregates on FeN_3_S and FeN_3_ (Figure 2d; Figures S20 and S22, Supporting Information). Furthermore, hematite Fe_2_O_3_ was also used as a control to exclude the formation of aggregated nanoparticles after pyrolysis (Figure 2d and Figure S21, Supporting Information). Thereby, these results confirm that Fe species exist exclusively as single atoms.^[^ ^66^ ^]^

### 2.3 Multi‐Enzyme Activity Performance of Fe‐SACs

**Figure 3:**
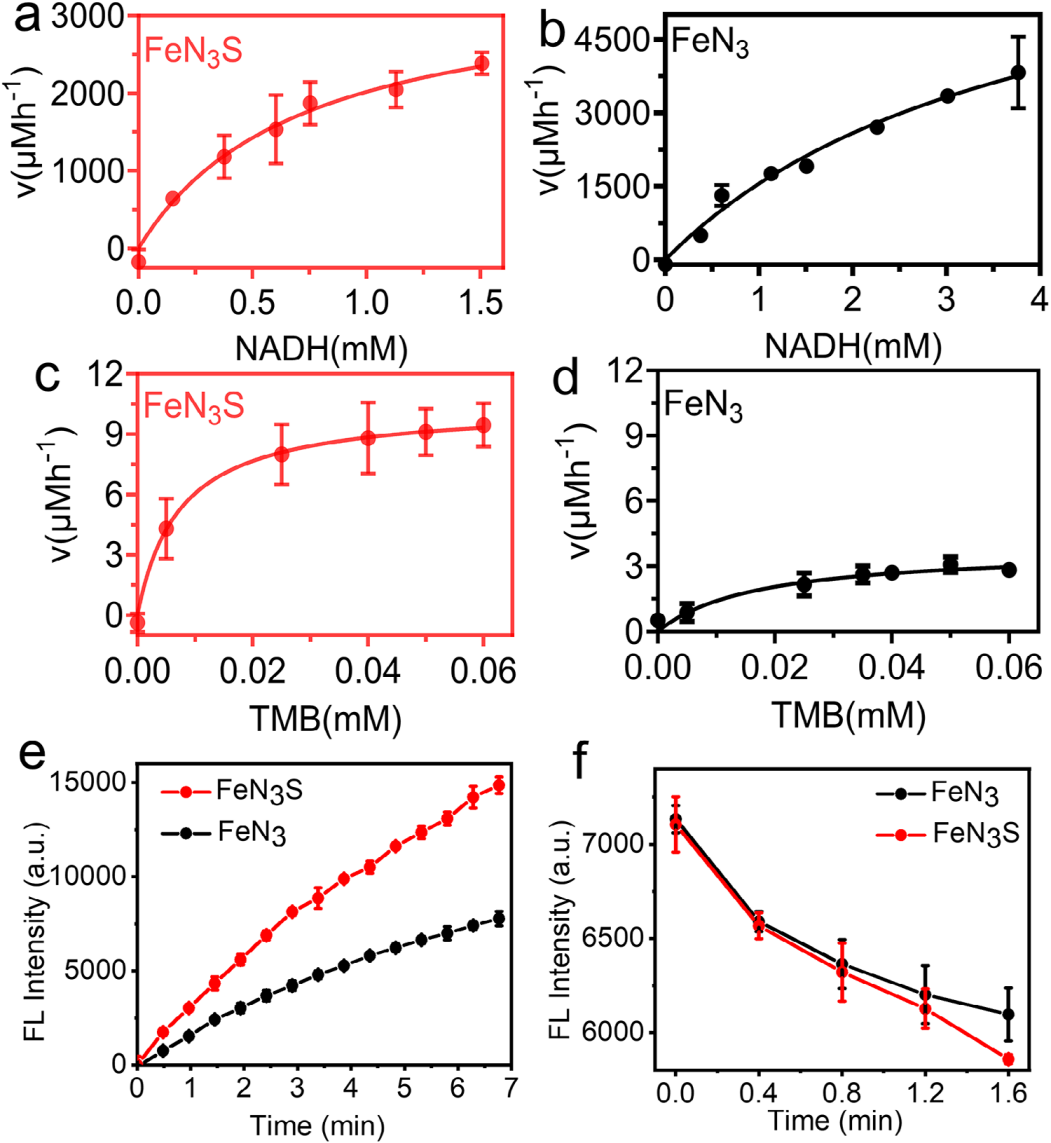
a,b) Michaelis–Menten kinetic analysis of NOX‐like activity of FeN_3_S and FeN_3_. Data are shown as mean ± standard deviation (SD) (*n* = 3). c,d) Michaelis–Menten kinetic analysis of OXD‐like activity of FeN_3_S and FeN_3_. Data are shown as mean±SD (*n* = 3). e) ·OH generation detected by TA (excitation at 320 nm, emission at 425 nm) with each sample containing equal Fe single‐atom loading. Data are shown as mean ± SD (*n* = 3). f) O_2_ generation detected by [Ru(bpy)_3_]^2+^ (excitation at 450 nm, emission at 625 nm) with each sample containing equal Fe single‐atom loading. All experiments were conducted in NaAc/HAc buffer pH 4.5. Data are shown as mean ± SD (*n* = 3).

NOX‐like activity was first evaluated. In this reaction, NADH is catalytically oxidized to NAD^+^ by Fe SAC, and the progress of the reaction was monitored at 340 nm using a plate reader (Figure S23, Supporting Information). All NADH oxidation reactions followed typical Michaelis–Menten kinetics. The catalytic efficiency (K_cat_/K_m_) of FeN_3_S was determined to be 267.42 µm ^−1^h^−1^, whereas that of FeN_3_ was 65.99 µm ^−1^h^−1^, corresponding to a 4.1‐fold enhancement for FeN_3_S (**Figure** **3**a,b). However, increasing the pyrolysis temperature to 900 °C led to a significant decrease in the catalytic efficiency of both FeN_3_S‐900 and FeN_3_‐900, with FeN_3_S‐900 even exhibiting lower activity than FeN_3_‐900, highlighting the critical influence of pyrolysis temperature on Fe SAC performance (Figure S24, Supporting Information). In contrast, the control samples NC, NC‐900, SNC, and SNC‐900, which lack Fe single atoms, displayed very low catalytic efficiencies of only ≈ 4–6 µm ^−1^h^−1^ (Figure S24, Supporting Information), without considering the possible coupling effect between Zn and Fe, indicating the indispensable role of Fe single atoms in NOX‐like activity. Next, OXD‐like activity was assessed using a 3,3′,5,5′‐tetramethylbenzidine (TMB) colorimetric assay. In this process, the Fe‐SAC catalyzes the oxidation of TMB to a blue‐colored product in the presence of O_2_, which exhibits a characteristic absorbance peak at ≈652 nm. FeN_3_, FeN_3_‐900, FeN_3_S, and FeN_3_S‐900 all displayed typical Michaelis–Menton behavior in their TMB oxidation, and the catalytic efficiency of FeN_3_S reached 65.71 µm ^−1^h^−1^, corresponding 4.6‐fold improvement over FeN_3_. However, after pyrolysis at 900 °C, the OXD‐like activity of FeN_3_S‐900 and FeN_3_‐900 decreased, with FeN_3_S‐900 showing only a 1.35‐fold higher than that of FeN_3_‐900 (Figure 3c,d; Figure S25a,b, Supporting Information). In contrast, NC, NC‐900, SNC, and SNC‐900 showed negligible catalytic activity, further confirming the indispensable role of Fe single atoms in OXD‐like activity Figure S25c–f, Supporting Information).

Furthermore, POD‐like activity of Fe SAC was evaluated. Terephthalic acid (TA) was employed to semi‐quantify ·OH in the catalytic system, as it reacts with ·OH to form the fluorescent compound 2‐hydroxyterephthalic acid.^[^ ^67^, ^68^ ^]^ The POD‐like activity of FeN_3_S was approximately twofold higher than that of FeN_3_ with each sample containing equal Fe single‐atom loading (Figure 3e). However, further increasing the pyrolysis temperature to 900 °C reduced the POD‐like activity (Figure S26a,b, Supporting Information). A gradual increase in fluorescence intensity at 420–460 nm was observed for FeN_3_S with increasing H_2_O_2_ concentration (Figure S26c, Supporting Information). In contrast, no such fluorescence signal was detected for NC, NC‐900, SNC, and SNC‐900 (Figure S26d, Supporting Information), confirming that the POD‐like activity was induced by Fe single atoms. The enzymatic reaction mechanism of Fe‐SACs was further investigated by electron spin resonance (ESR) spectroscopy. ·OH generation was detected during the POD‐like catalytic process in the presence of FeN_3_S, FeN_3_, FeN_3_S‐900, or FeN_3_ ‐900 (Figures S27 and S28, Supporting Information). More importantly, the additional H_2_O_2_ generated by the NOX‐like activity also induced the POD‐like activity of FeN_3_S (Figure S27, Supporting Information), indicating a cascade reaction between the NOX‐ and POD‐like activities of FeN_3_S. Finally, [Ru(bpy)_3_]^2+^, a strongly luminescent complex that emits intense orange luminescence under UV excitation (*λ*_exc_ = 450 nm), was employed to probe CAT‐like activity. Since the luminescence of [Ru(bpy)_3_]^2+^ can be quenched by O_2_, it serves as an effective probe for monitoring O_2_ generation.^[^ ^69^ ^]^ FeN_3_S exhibited a 1.35‐fold higher O_2_ generation capacity than FeN_3_, with each sample containing equal Fe single‐atom loading (Figure 3f). A slight increase in O_2_ generation was observed for FeN_3_S‐900 (Figure S29a,b, Supporting Information). Moreover, a decrease in fluorescence intensity at 550–700 nm was observed for FeN_3_S with increasing H_2_O_2_ concentration (Figure S29c, Supporting Information), indicating enhanced O_2_ generation. In contrast, no fluorescence signal was detected for NC, NC‐900, SNC, and SNC‐900 (Figure S29d, Supporting Information), confirming that the CAT‐like activity originated from Fe single atoms. Although a significant Zn content (290–1800 mm kg^−1^) in the catalyst was found (Table S2, Supporting Information), Figures S25, S26 and S29 (Supporting Information) show that NC, SNC, SNC‐900, and NC‐900 exhibit negligible OXD‐, POD‐, and CAT‐like activities, while Figure S24 (Supporting Information) indicates that Zn SAC display some NOX‐like activity. To eliminate the potential contribution of Zn to NADH catalysis, the catalytic efficiency of Zn was subtracted from that of Fe‐SACs based on ICP results (Figure S24, Supporting Information). Our previous work demonstrated that there is no synergistic effect between Zn and Fe single atoms, as confirmed by pyrolyzing pristine ZIF‐8 at 1000 °C to completely remove Zn, followed by re‐doping with Zn and Fe.^[^ ^29^, ^63^, ^70^ ^]^ This observation is consistent with earlier reports, which also found no synergistic effect between Zn and Fe in Zn/Fe dual‐atom systems.^[^ ^71^ ^]^ Therefore, the possibility that the enhanced activity originates from Fe–Zn dual‐atom sites was ruled out. Since the same amount of Fe was added during MOF synthesis, both FeN_3_ and FeN_3_S exhibited similar Fe contents after pyrolysis (161.25 vs 165.73 mm kg^−1^). Although FeN_3_S‐900 showed the highest Fe content (176.11 mm kg^−1^) (Table S2, Supporting Information), this was likely due to Zn evaporation at 900 °C. However, as the multi‐enzyme activity is determined by the activity of a single Fe atom, the influence of overall Fe loading on multi‐enzyme performance can be considered negligible. In addition, other S‐containing ligands (1,2‐ethanedithiol, 1‐butanethiol, and thioglycolic acid) serve as alternatives; however, no NOX‐, OXD‐, POD‐, or CAT‐like activities were observed despite Fe loading (Figure S30; Table S2, Supporting Information). This indicates that the choice of S‐ligand is critical for enhancing multi‐enzyme‐like activity, otherwise, it may hinder or completely inactivate the catalytic functions. In summary, the multi‐enzyme activity of FeN_3_S primarily arises from the synergistic effects between the MOF‐derived carbon structures and Fe single atoms, while the S from Cys induced asymmetric coordination of Fe further amplifies these activities.

The temperature‐dependent and pH‐dependent multi‐enzyme‐like catalytic activities of FeN_3_S were also investigated (Figure S31a,b). The NOX‐like and POD‐like activities remained relatively stable and retained considerable activity within the temperature range of 20 to 50 °C, indicating good thermal tolerance (Figure S31a). In contrast, both OXD‐like and CAT‐like activities exhibited significantly reduced performance at lower temperatures but reached their optimal catalytic activity at 40 °C (Figure S31a). These results suggest that the temperature sensitivity of the nanozyme varies depending on the type of enzymatic function it mimics, with certain activities (e.g., OXD‐like and CAT‐like) being more reliant on thermal activation to achieve effective catalysis. Overall, 40 °C is most suitable for all enzymatic functions. In terms of pH dependence, POD‐like activity increased and then decreased with pH, showing a maximum at pH 4.5. OXD‐like activity remained nearly the same at pH 3 and pH 4.5 but decreased at higher pH values. NOX‐like activity decreased with increasing pH, whereas CAT‐like activity increased with pH (Figure S31b). Finally, the stability of FeN_3_S has also been investigated. The FeN_3_S demonstrates significant stability at various high temperature (20–100 °C) and pH (2–9) environments (Figure S31c,d), implying their potential for industrial applications.

### 2.4 Density Functional Theory (DFT) Study on Multi‐enzyme‐like Activity of Fe‐SACs

**Figure 4:**
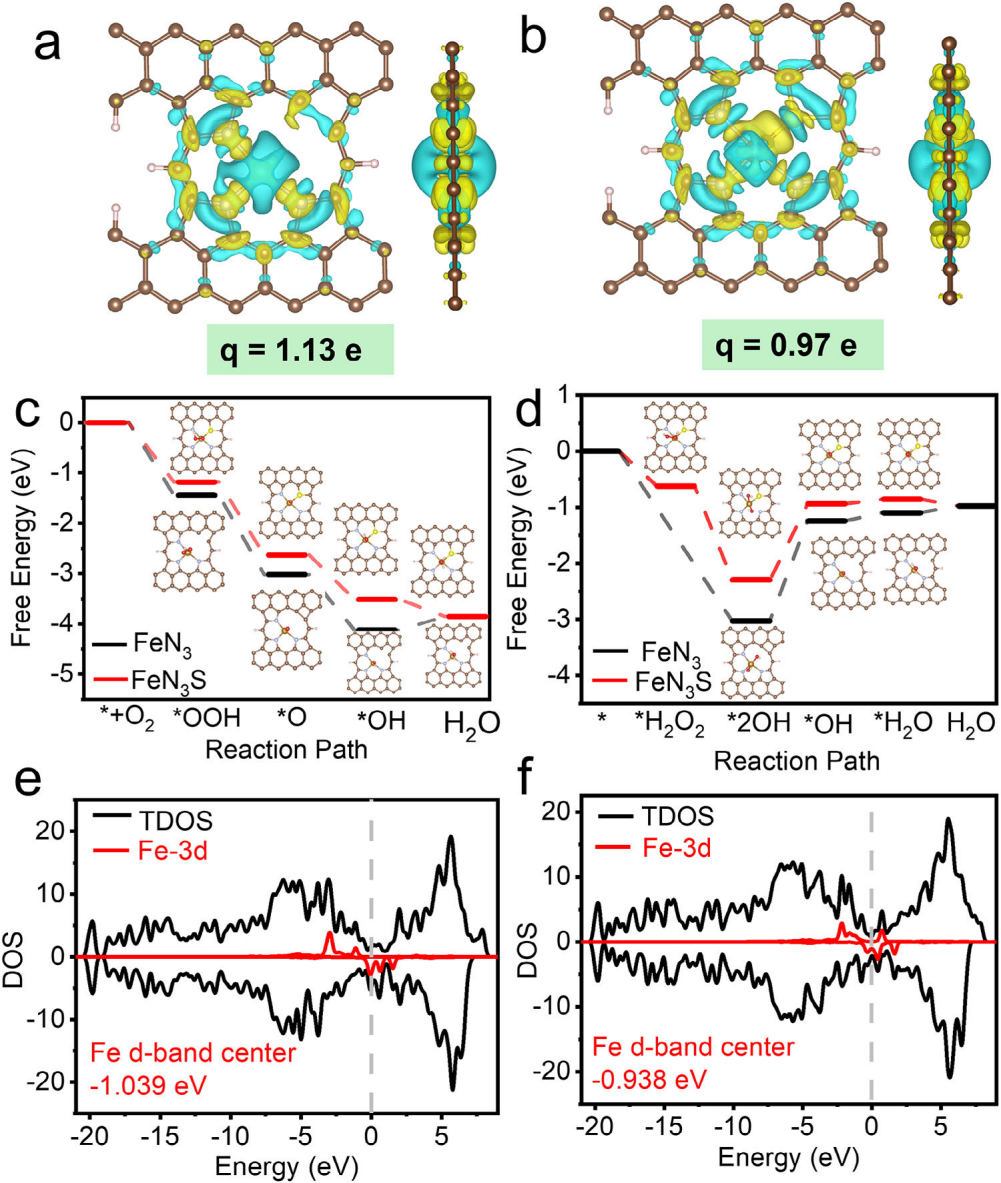
DFT calculation for FeN_3_ and FeN_3_S. a,b) The charge density difference and Bader charges of a) FeN_3_ and b) FeN_3_S. c) Free energy diagram for OXD‐like catalytic reaction on FeN_3_ and FeN_3_S, and their schematic illustration of the mechanism. d) Free energy diagram for POD‐like catalytic reaction of FeN_3_ and FeN_3_S, and their schematic illustration of the mechanism. e,f) DOS and Fe d‐band center of FeN_3_ and FeN_3_S. Color code: Fe, dark yellow; C, brown; N, blue; O, red; H, light pink; S, bright yellow.

To further elucidate the origin of the enhanced multi‐enzyme catalytic activities of FeN_3_S, the potential catalytic mechanism of FeN_3_ and FeN_3_S was investigated using DFT calculations. Although pyridinic N is generally very stable, Raman spectra indicate that numerous defects are generated during pyrolysis (Figure S9, Supporting Information). XPS analysis further shows that, in FeN_3_, 20.13% of the N species are pyridinic, 14.67% are graphitic, and 61.55% are pyridine‐N (Figure S11, Supporting Information). Previous studies have also reported that structural rearrangements of carbon precursors during pyrolysis (800–1000 °C) can generate carbon vacancies (C_v_).^[^ ^72^, ^73^ ^]^ Such defect formation likely accounts for the absence of a pyrrole ring in FeN_3_. Consistent with this, similar FeN_3_ vacancies have been described in the literature,^[^ ^74^, ^75^ ^]^ which exhibit well‐balanced free energy barriers for reaction intermediates. Bader charge analysis demonstrates electron accumulation between the central Fe and S atoms.^[^ ^76^, ^77^ ^]^ Fe atom carries charges of 1.13 |e| and 0.97 |e| in FeN_3_ and FeN_3_S, respectively (**Figure** **4**a,b), indicating that the electron localization around the Fe─S and Fe─N bonds (green regions between Fe and S, and Fe and N) in FeN_3_S is less pronounced than that around the Fe─N bonds in FeN_3_ (Figure 4a,b). This can be attributed to the weaker electron‐withdrawing nature of S compared to N.^[^ ^76^ ^]^ In principle, a higher Bader charge corresponds to a higher oxidation state. However, it has been reported that Bader charges are not always reliable indicators of oxidation states.^[^ ^78^, ^79^ ^]^ In this case, the Bader charge trend appears opposite to the formal oxidation state of FeN_3_S, which is more positively charged (Figure 2b). This discrepancy may arise from charge delocalization within the framework, resulting in an uneven redistribution of electron density,^[^ ^80^ ^]^ which in turn significantly alters the catalytic activity.

For the case of the OXD‐like process, O_2_ first adsorbs onto the Fe single‐atom site to form an adsorbed intermediate (Figure 4c).^[^ ^81^ ^]^ Although FeN_3_ shows stronger O_2_ adsorption capacity, its higher ^*^O free energy leads to a large energy barrier in the final step, which requires a substantial barrier of 0.27 eV in the final step, whereas this barrier vanishes in the FeN_3_S pathway. This suggests a more favorable thermodynamic and kinetic profile of FeN_3_S for OXD‐like catalysis (Figure 4c). In the POD‐like catalytic process, adsorption of H_2_O_2_ molecules on the Fe single atom site occurs first (Figure 4d). FeN_3_ exhibits stronger H_2_O_2_ adsorption, after which the adsorbed H_2_O_2_ undergoes homolytic dissociation to form two ^*^OH groups. The subsequent desorption of ·OH radicals from the Fe single atom site is the rate‐limiting step^[^ ^82^ ^]^ (Figure 4d). Here, FeN_3_S demonstrates a lower energy barrier (1.78 eV) compared with FeN_3_ (2.21 eV), making ·OH formation more favorable (Figure 4d).^[^ ^83^ ^]^ Meanwhile, the synergistic coordination of Fe with N and S effectively optimizes the transition‐state free energy. Thus, the central Fe atom and the asymmetric spatial configuration of FeN_3_S are the key factors underlying its superior enzyme‐like activity.

Furthermore, density of states (DOS) analysis offered additional insights into the catalytic mechanism underlying the enhanced NOX‐, OXD‐, POD‐, and CAT‐like activities. The DFT‐calculated DOS results provide clear evidence that S coordination profoundly tunes the electronic structure of the Fe center. The introduction of S in FeN_3_S leads to a noticeable increase in the DOS near the Fermi level (*E*_f_), accompanied by the formation of new hybrid orbitals (Figure 4e,f). Furthermore, the activation of Fe by S doping is confirmed by the upward shift of the Fe d‐band center (*ε*_d_) in Fe‐N_3_S (−0.938 eV) compared with that in FeN_3_ (−1.309 eV) (Figure 4e,f), implying that the Fe 3d orbitals in FeN_3_S approach the Fermi level and become more chemically active. Such an upshift indicates stronger electronic interactions and more efficient charge transfer between FeN_3_S and the reaction substrates than in FeN_3,_^[^ ^47^, ^84^ ^]^ consistent with the frontier orbital theory, thereby enhancing its catalytic activity.

EPR spectroscopy was employed to probe the spin states of the Fe active site. As shown in Figure S32 (Supporting Information), a notable difference is observed between the EPR spectra of FeN_3_ and FeN_3_S. The FeN_3_S sample displays a broader peak, whereas FeN_3_ exhibits a sharp resonance signal, reflecting the increased electron density around Fe due to the formation of an asymmetric, low‐coordinated FeN_3_ structure.^[^ ^85^ ^]^ The reduced signal intensity in FeN_3_S supports a transition from a high‐spin (↑ ↑ ↑) to a low‐spin (↑) state of the Fe atoms.^[^ ^86^ ^]^ These changes in signal intensity, along with the g‐value shift from 2.002 (FeN_3_) to 2.088 (FeN_3_S), indicate a spin‐state transition upon S doping, suggesting the pivotal role of S incorporation in modulating the electronic structure and magnetic properties of the Fe centers. Unlike previous reports that aim to develop high‐spin Fe sites, which possess more unpaired electrons in the e_g_ orbitals and facilitate substrate penetration to enhance adsorption,^[^ ^87^ ^]^ our results show that excessively strong substrate adsorption on FeN_3_ is detrimental to catalytic activity, as it slows the desorption of intermediates, the rate‐limiting step (Figure 4c,d). Consequently, FeN_3_S achieves overall improved multi‐enzyme activity.

### 2.5 Pore Size Analysis

The N_2_ adsorption–desorption isotherms, the corresponding Brunauer–Emmett–Teller (BET) surface area, and pore size distribution were further analyzed to support the improved multi‐enzyme activity (Figures S33 and S34; Table S5, Supporting Information). For adsorption characterization at low P/P_0_, FeN_3_S and FeN_3_ exhibit features typical of microporous carbon materials. Nitrogen uptake begins at very low relative pressures (≈5 × 10^−6^ P/P_0_) (Figure S33a,b, Supporting Information), indicating the presence of slit‐shaped pores with intra‐wall ultramicropores (< 1.2 nm) accessible to N_2_ molecules.^[^ ^88^ ^]^ In the case of FeN_3_S‐900 and FeN_3_‐900 (Figure S33c,d, Supporting Information), a small hysteresis loop appears in the adsorption–desorption isotherm, characteristic of adsorption in interconnected mesopores.^[^ ^89^ ^]^ The open‐ended isotherms observed for samples pyrolyzed at 800 and 900 °C at low relative pressures can be attributed to the formation of abundant ultramicropores at 800 °C and structural defects‐induced mesopores at 900 °C during high‐temperature pyrolysis. These features enhance adsorbate–adsorbent interactions and hinder complete desorption,^[^ ^90^ ^]^ resulting in nonclosure of the isotherm at low P/P_0_. The results are consistent with the pore size analysis (Figure S34; Table S5, Supporting Information). It was found that all samples pyrolyzed at 800 °C retained the original MOF pore size (≈1 nm), whereas those pyrolyzed at 900 °C exhibited enlarged pores of 3–10 nm. Moreover, the 900 °C samples showed a dramatic reduction in BET surface area (139–764 m^2^ g^−1^) compared with the 800 °C samples (831–1627 m^2^ g^−1^), along with a decrease in total pore volume from 0.5–0.7 to 0.2–0.4 cm^3^ g^−1^. This can be ascribed to the Zn evaporation, structural collapse, and enhanced graphitization induced by the elevated pyrolysis temperature, as indicated by the reduction of I_D_/I_G_ values in Raman spectra (Figure S9, Supporting Information). This graphitization process promotes the formation of a denser carbon framework, resulting in the partial elimination of micropores and consequently diminishing the specific surface area.^[^ ^91^ ^]^ It is found that the high pore volumes and surface areas of support remarkably promoted the exposure of active sites and mass transport of intermediates, thus significantly improving catalytic performance.^[^ ^92^, ^93^ ^]^ Therefore, FeN_3_S demonstrates higher NOX, OXD, CAT and POD‐like activity than FeN_3_S‐900.

Compared with previous reports (Table S6, Supporting Information), asymmetric SACs were typically developed by mixing supports with heteroatom‐containing chemicals and metal precursors, without precise control over heteroatom doping. For example, glucosamine and dicyandiamide were employed as carbon sources to anchor single atoms,^[^ ^76^ ^]^; thiourea,^[^ ^76^ ^]^ trithiocyanuric acid^[^ ^77^ ^]^ and bis(4‐hydroxyphenyl) sulfone,^[^ ^94^ ^]^ were used to introduce S; while poly‐(cyclotriphospazene‐co‐4,4′‐diaminodiphenylether)^[^ ^7^ ^]^ and phosphonitrilic chloride trimer^[^ ^94^ ^]^ were used to introduce P. However, these approaches typically yielded catalysts with only single or bi‐enzyme activity. In some cases, additional natural enzymes had to be loaded to achieve better performance,^[^ ^95^ ^]^ which limits their broader applications. In contrast, our work employs a precisely designed heteroatom‐doping strategy to develop asymmetric FeN_3_S SACs with enhanced catalytic functionality: i) the highly dispersed Fe single atoms originating from biomineralization. The thiolate group of Cys serves as the primary binding site for Fe, while the amine group of Cys promotes MOF biomineralization, enabling the formation of highly dispersed Fe single atoms; ii) the large surface area and pore volume retained from the original MOF. After pyrolysis at 800 °C, the MOF retains its original pore size, surface area, and pore volume; iii) S‐doping tunes the electronic structure of the Fe single atom, reducing activation barriers, enhancing substrate interactions, and facilitating charge transfer. Collectively, these effects contribute to the improved multi‐enzyme activity of Fe SAC. In addition, this strategy could potentially be applied to other metal single atoms owing to the metal cation accumulation ability of Cys.^[^ ^50^ ^]^

### 2.6 The Role of Fe‐SACs in Suppressing Tumor Cell Growth

**Figure 5:**
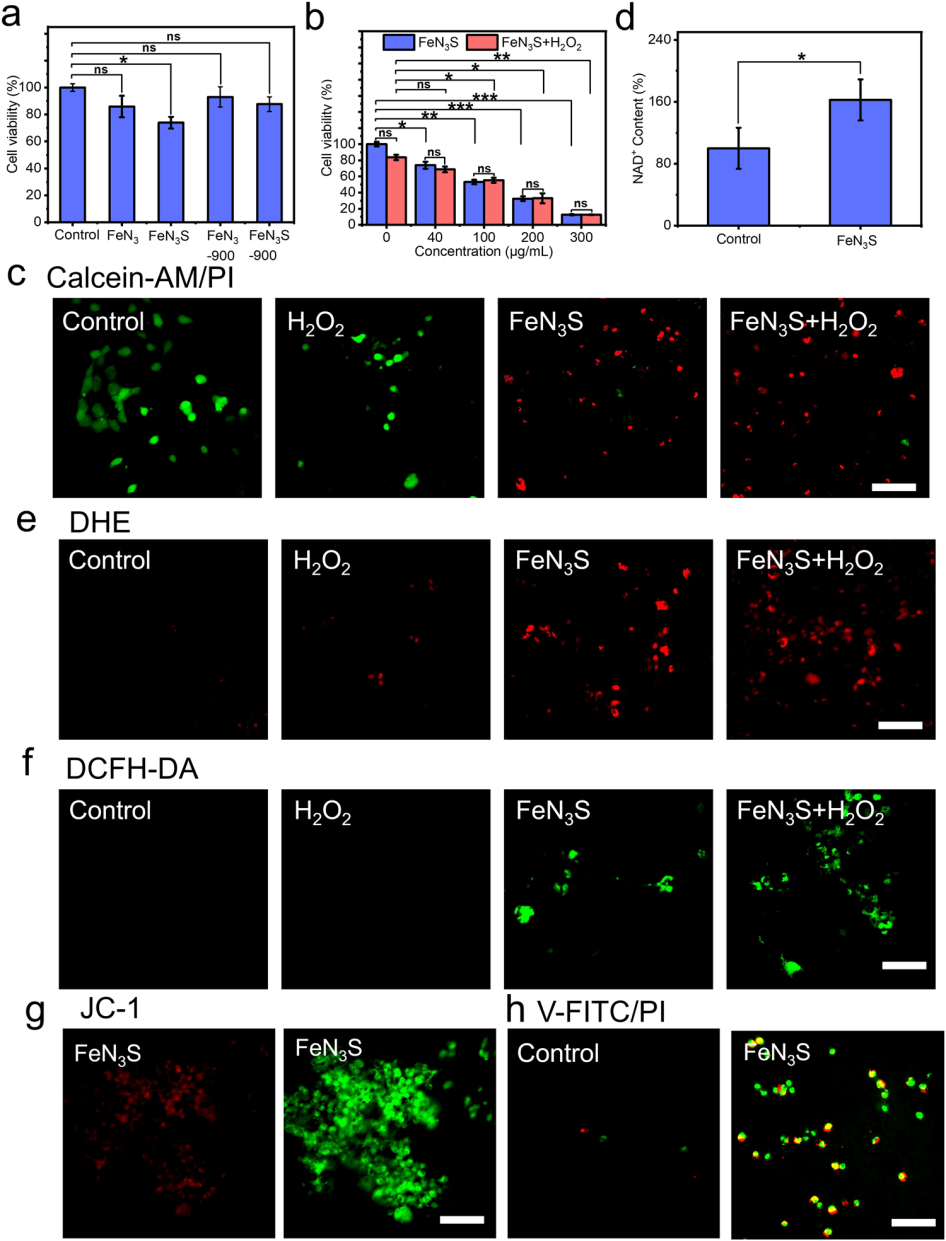
Cytotoxicity assessment of 4T1 cells after being treated with a), FeN_3_, FeN_3_S‐900, and FeN_3_‐900; Quantitative data are shown as mean ± SD (*n* = 3). One‐way ANOVA and Sidak's multiple comparisons test were performed. b) different concentrations of FeN_3_S with or without 100 µm H_2_O_2_ addition. Quantitative data are shown as mean ± SD (*n* = 3). Two‐way ANOVA (mixed model) and Sidak's multiple comparisons test were performed. c) Live/dead cell fluorescence microscopy images of 4T1 cells stained with Calcein‐AM and propidium iodide (PI) after treatment with H_2_O_2_, FeN_3_S, and FeN_3_S+H_2_O_2_. Scale bar, 100 µm. d) The NAD^+^ content of cancer cells with or without FeN_3_S treatment using the NAD^+^ /NADH Assay Kit. Quantitative data are shown as mean ± SD (*n* = 3). Two‐tailed unpaired *t*‐test was performed for the comparison between the two groups. e) Intracellular ·O_2_^−^ generation probed by DHE after treatment with H_2_O_2_, FeN_3_S, and FeN_3_S+H_2_O_2_. f) Intracellular total ROS generation probed by DCFH‐DA after treatment with H_2_O_2_, FeN_3_S, and FeN_3_S+H_2_O_2_. The scale bar is 50 µm in c, e‐f. g) Fluorescence microscopy images showing mitochondrial depolarization in 4T1 cells cultured with FeN_3_S (200 µg mL^−1^) for 24 h. Scale bar, 100 µm. Representative fluorescence images from n = 3 independent experiments in c, e‐g (each experiment: 3 wells per condition, ≥ 5 random fields per well). Acquisition settings were identical across groups. h) Fluorescence microscopy images of 4T1 cells cultured without and with FeN_3_S (200 µg mL^−1^) for 24 h and stained with Annexin V‐FITC/PI. Scale bar, 20 µm. Two‐tailed unpaired *t*‐test was performed for the comparison between the two groups. Statistical significance was denoted as ns (*p* ≥ 0.05), ^*^ (*p* < 0.05), ^**^ (*p* < 0.01), ^***^ (*p* < 0.001), and ^****^ (*p* < 0.0001).

Given the outstanding multi‐enzyme activity of FeN_3_S, its potential in suppressing tumor cell growth was further investigated through a series of in vitro cytotoxicity assays on 4T1 cells. As shown in **Figure** **5**a, FeN_3_S exhibited the most effective suppression of tumor cell growth among FeN_3_, FeN_3_S‐900, and FeN_3_‐900 at 40 µg mL^−1^. Furthermore, the cytotoxicity of FeN_3_S was dose‐dependent, with cell viability decreasing as its concentration increased. At 300 µg mL^−1^, FeN_3_S induced ≈90% cell death within 24 h. However, no significant difference was observed between FeN_3_S with or without H_2_O_2_ addition (Figure 5b), suggesting that intracellular H_2_O_2_ generated from cell metabolism was sufficient to drive FeN_3_S‐activated catalytic reactions.^[^ ^7^ ^]^ A control experiment using normal 3T3 fibroblasts was also conducted. The results showed that over 80% of cells remained viable at concentrations below 100 µg mL^−1^, while ≈60% remained viable at 200–300 µg mL^−1^ (Figure S35, Supporting Information), indicating a certain degree of selectivity of FeN_3_S toward tumor cells. Fluorescence imaging confirmed extensive cell death (Figure 5c). The long‐term stability and degradation behavior of FeN_3_S in biological environments were systematically evaluated. FeN_3_S was incubated with cells in an intracellular‐mimicking medium containing 10% serum and 90% culture medium for 24 and 72 h. After 24 h, 12.2% of Fe and 19.7% of Zn were released, whereas after 72 h, 12.7% of Fe and 36% of Zn were released (Table S7, Supporting Information). In addition, 4000 ng/mL of S was detected after 72 h. The potential cytotoxicity of the released Fe, S, and Zn species toward healthy 3T3 fibroblasts was subsequently assessed. The results indicated that Fe had no appreciable effect on cell viability, while S induced a slight decrease. Notably, co‐doping Fe and Zn with S did not cause any additional reduction in cell viability (Figure S36, Supporting Information).

It is found that FeN_3_S can effectively increase NAD^+^ content (60%) in cancer cells by mimicking NOX‐like activity (Figure 5d), indicating the disruption of the NADH/NAD^+^ cycle among cancer cells. Then, the ·O^2−^ at the cellular level was detected by specific fluorescent probes dihydroethidium (DHE). Enhanced red fluorescence was measured in 4T1 cells after being treated with FeN_3_S (Figure 5e), indicating that FeN_3_S can generate ·O_2_^−^ in cells through OXD‐like activity. However, there is no noticeable signal in the presence of the H_2_O_2_ (Figure 5e). Furthermore, ·O_2_^−^ production of FeN_3_S could be further enhanced after adding H_2_O_2_ due to its intrinsic CAT‐like activity. Meantime, a significantly increased green fluorescence was observed after treatment with FeN_3_S, indicating total intracellular ROS generation (including ·OH and ·O_2_^−^) (Figure 5f). Similar to the ·O_2_^−^, there is no signal detected in the presence of the H_2_O_2_ alone, indicating the ROS was produced by FeN_3_S rather than by intracellular H_2_O_2_.

To further investigate mitochondrial effects and apoptotic/necrotic pathways, additional experiments were performed. The mitochondrial membrane potential (MMP) is crucial for mitochondrial homeostasis and serves as a key indicator of cell apoptosis. A sustained decrease in MMP from normal levels signals cell death and associated pathologies.^[^ ^84^ ^]^ Therefore, JC‐1 staining (5,5‘,6,6‘‐tetrachloro‐1,1′,3,3′‐tetraethylbenzimidazolyl‐carbocyanine iodide) was employed to assess MMP. Compared with the control (Figure S37, Supporting Information), FeN_3_S treatment decreased red fluorescence and increased green fluorescence (Figure 4g), indicating MMP reduction and cell apoptosis, consistent with elevated ROS levels (Figure 5f). Additionally, ATP production in FeN_3_S‐treated cells was significantly inhibited, with ATP levels reduced by ≈24.7% relative to controls (Figure S38, Supporting Information). Thus, the inhibition of energy production in cancer cells may result from disruption of the NADH/NAD⁺ cycle, as endogenous NADH is essential for transmembrane proton translocation and for maintaining the proton motive force required for ATP synthesis.^[^ ^47^ ^]^ Apoptosis analysis using annexin V‐FITC/PI staining revealed that 41.5% of 4T1 cells were positive for both V‐FITC and PI, indicating late apoptosis or necrosis, while 25% were V‐FITC positive but PI negative, reflecting early apoptosis with intact membranes. Only 1% of cells were PI positive but V‐FITC negative, suggesting minimal direct necrotic death (Figure 5h; Table S8, Supporting Information). These results demonstrate that FeN_3_S predominantly induces apoptosis in 4T1 cells.

In comparison, the single‐enzyme catalysts Fe‐N_4_,^[^ ^96^ ^]^ noble‐metal (e.g., Pt/Pd)‐N_4_,^[^ ^97^ ^]^ and bi‐enzyme Cu‐N_4_^[^ ^98^ ^]^ achieved only limited therapeutic effects, inducing 56% mortality of 4T1 cells within 10 h, 70% mortality of CNE1^LMP1^ cells after 24 h, and 63% mortality of 4T1 cells after 24 h, respectively (Table S9, Supporting Information). Although other reported systems such as BSA‐Cu, Fe‐N_5_, and Cu‐N_4_ exhibited multi‐enzyme activities (POD, OXD, glutathione oxidase (GSHOx) for BSA‐Cu,^[^ ^99^ ^]^ OXD, POD, GSHOx for Fe‐N_5_,^[^ ^100^ ^]^ and POD, CAT, OXD, GSHOx for Cu‐N_4_^[^ ^101^ ^]^), their therapeutic efficacy remained moderate, with only 66.2% mortality of HCT116 cells after 24 h, 59% mortality of 4T1 cells after 24 h, and ≈78% mortality of 4T1 cells after 12 h, respectively. Moreover, the multi‐enzyme Ir–N_5_ SAC induced merely ≈60% 4T1 cell death under comparable conditions.^[^ ^47^ ^]^ Therefore, FeN_3_S catalyst exhibited stronger tumor cell suppression than other reported SACs (Table S9, Supporting Information), highlighting the superior therapeutic efficiency of FeN_3_S. In summary, the developed FeN_3_S integrates the advantages of multi‐enzyme properties (NOX, OXD, POD, and CAT) induce cell apoptosis (Figure S39, Supporting Information): i) The NOX‐like property of FeN_3_S perturbs intracellular redox equilibrium and disrupts metabolic stability in cancer cells, thereby sensitizing them to oxidative stress. ii) The OXD‐ and POD‐like activities further accelerate ROS generation, leading to oxidative damage that inhibits cell growth and promotes apoptotic signaling pathways. iii) The CAT‐like activity of FeN_3_S unexpectedly enhances ·O_2_^−^ production, amplifying oxidative stress. Collectively, these synergistic redox activities shift the intracellular balance toward excessive ROS accumulation. This oxidative overload compromises mitochondrial integrity, decreases ATP production, and triggers apoptosis as the dominant mode of cell death. These characteristics highlight its strong potential as a therapeutic agent in cancer treatment. However, the biological evaluation in this study is limited to 4T1 cells and 3T3 fibroblasts in vitro. To further advance the translation of FeN_3_S for cancer therapy, in vivo studies will be required in the future. To adapt FeN_3_S for in vivo applications, surface modification strategies such as PEGylation^[102]^ or coating with zwitterionic polymers^[103]^ can be employed to form hydrophilic barriers that minimize protein adsorption and reduce immune recognition. To enhance targeted delivery, FeN_3_S can be further functionalized with macrophage membranes^[104]^ or site‐specific antibodies^[105]^, enabling selective accumulation at inflammatory sites.

## 3 Conclusion

A metallothionein‐inspired heteroatom doping strategy was employed to engineer an asymmetric FeN_3_S nanozyme. FeN_3_S exhibited 1.35‐4.60‐fold higher catalytic efficiency in NOX‐, OXD‐, POD‐, and CAT‐like activities compared with FeN_3_ without S doping. The highly efficient multi‐enzyme activity of FeN_3_S can be attributed to the highly dispersed single atoms originating from the biomineralization, the large surface area and pore volume retained from the original MOF, and the S‐doping achieved through the strong mercaptide coordination between Fe and S. The S doping not only tunes electronic structure of Fe single atom to reduce activation barriers and enhances substrate interaction, but also facilitates charge transfer. Owing to its highly efficient multi‐enzyme activity, FeN_3_S achieved ≈90% cancer cell death within 24 h. This work highlights a promising strategy for developing advanced SACs with multi‐enzyme activity for complex environmental and biomedical applications.

## 4 Experimental Section

### Reagent and Materials

Zinc nitrate hexahydrate (98.0%), 2‐methylimidazole (HmIm, 99.0%), L‐cysteine methyl ester hydrochloride (Cys), 1,2‐echanedithiol, 1‐butanethiol and thioglycolic acid, iron(III) chloride (FeCl_3_), hydrogen peroxide (H_2_O_2_), 3,3′,5,5′‐tetramethylbenzidine (TMB), nicotinamide adenine dinucleotide (NADH), terephthalic acid (TA), tris(2,2′‐bipyridine)ruthenium(II) hexafluorophosphate (Tris II), NAD⁺/NADH Assay Kit (MAK460), Annexin V‐FITC Apoptosis Detection Kit, fluorescent probes dihydroethidium (DHE), acetoxymethyl ester form (Calcein‐AM), propidium Iodide (PI), 2′,7′‐Dichlorodihydrofluorescein diacetate (DCFH‐DA), NAD^+^ /NADH Assay Kit, dimethyl sulfoxide (DMSO), thiazolyl blue tetrazolium bromide and phosphate‐Buffered Saline (PBS) were purchased from Sigma Aldrich (Australia). DyLight^TM^ 633 NHS Ester, Zeba^TM^ Dye and biotin removal columns (0.5 mL), ATP Determination Kit (A22066), JC‐1 Dye (Mitochondrial Membrane Potential Probe) were purchased from Thermo Scientific (Australia). All other reagents were purchased from Sigma–Aldrich (Australia) and used without further purification.

### Catalyst Preparation

For the synthesis of Fe SACs, 40 mL of an aqueous solution containing 9.020 g of HmIm was first mixed with 0.704 mL of FeCl_3_ (0.023 m), 0.704 mL of Cys (0.023 m), or a combination of both (rapidly mix). To investigate the impact of different S precursors to the synthesis of Fe SACs, 1,2‐Echanedithiol, 1‐Butanethiol, and Thioglycolic acid were used to replace cysteine in equivalent molar amounts. After rapid mixing, immediately add 4 mL of an aqueous solution containing 327.4 mg of Zn (NO_3_)_2_·6H_2_O was added, and the mixture was stirred at room temperature for 30 min. The resulting products were then centrifuged at 12500 rpm, washed three times with DI water, and dried at 80 °C overnight. The dried samples were placed in a quartz boat and pyrolyzed at 800 or 900 °C for 2 h in a tube furnace under a flowing nitrogen atmosphere (10–20 mL min^−1^) with a heating rate of 5 °C min^−1^. After cooling to room temperature, the pyrolyzed samples were collected and ground in a mortar for further use.

### Characterizations

Scanning electron microscopy (SEM) images were taken on a FEI Nova NanoSEM 230 under 10 kV acceleration voltage with a secondary electron detector. The samples were coated with Pt using Emitech K575x evaporative premium coater before imaging. The crystallinity of enzyme‐ZIF‐8 and SAC was also measured by an Empyrean XRD Xpert Materials Research diffractometer (MRD) system with a Cu Kα anode (λ = 0.15406 nm) at 40 kV and 40 mA. Nitrogen adsorption‐desorption measurements were performed using a MicroActive for TriStar II Plus 2.02, samples were degassed by heating at 120 °C for 12 h under vacuum. An X‐ray photoelectron spectrometer (Thermo ESCALAB250Xi) was deployed to record the X‐ray photoelectron spectra (XPS). Raman spectra were obtained by a Renishaw Raman system at a 514 nm excitation. The metal ion content was determined by ICP‐OES (Optima7000 and Avio from PerkinElmer, USA). JEOL JEM‐F200 with electron acceleration energy of 200 kV was used to record bright field TEM and HRTEM images, and XEDS elemental mappings. Samples were prepared by dispersing in ethanol using a sonication bath and drop‐casting 10 µL of solution onto a holey carbon Cu film. A JEOL JEM‐GrandARM300F2 double aberration corrected scanning transmission electron microscope (STEM) operated at 300 kV was used to obtain annular dark field scanning (ADF) STEM images and electron energy loss spectroscopy (EELS) spectra of the pyrolyzed samples. ADF‐STEM images were acquired with a camera length of 200 mm with corresponding inner and outer acceptance angles of 32 and 175 mrad, respectively. Under these conditions, it was not possible to distinguish between the Fe and Zn species directly from the images due to the similar Z numbers of the two species and the varying background intensity due to thickness. To confirm the presence of Fe and Zn, EELS spectra were collected using a Gatan Image Filter (GIF) Continuum spectrometer equipped with a single electron detector, K3. The EELS data presented have been background‐subtracted. All nanozyme activity measurements were recorded by a CLARIOstar microplate reader (BMG LABTECH Germany) at room temperature.

### XAS Measurements and Data Processing

The X‐ray absorption fine structure spectra (Fe K‐edge) were collected at XAS Beamline (Australian Synchrotron, VIC, Australia), the data collection was carried out in transmission mode using an ionization chamber for Fe foil, and in fluorescence excitation mode using a Lytle detector for FeN_3_, FeN_3_, FeN_3_‐900, FeN_3_S‐900. All spectra were collected in ambient conditions. The XAS data were processed according to the standard procedures using the Athena module implemented in the IFEFFIT software packages. The EXAFS spectra were obtained by subtracting the post‐edge background from the overall absorption and then normalizing with respect to the edge‐jump step. Subsequently, the *χ(k)* data were Fourier transformed into real (R) space using a Hanning windows (*dk* = 1.0 Å^−1^) to separate the EXAFS contributions from different coordination shells. To obtain the quantitative structural parameters around central atoms, least‐squares curve parameter fitting was performed using the ARTEMIS module of IFEFFIT software packages.^[^ ^106^, ^107^ ^]^ Fitting of the magnitude of the Fourier transform of the *k3*‐weighted EXAFS (data‐blue and fit‐red) for the sample and standards, and the corresponding EXAFS fitting curves of Sample and Standards at *k* space. For Wavelet Transform analysis, the *χ(k)* exported from Athena was imported into the Hama Fortran code.^[^ ^108^ ^]^ The parameters were listed as follows: *R‐*range, 0.0–6.0 Å, *k‐*range, 0–14.0 Å^−1^ for sample and standards; *k* weight, 2; and Morlet function with *κ *= 8, *σ *= 1 was used as the mother wavelet to provide the overall distribution.

### Preparation of Dye‐Labeled Cys

Cys were tagged with NHS‐633 ester. The mixtures of Cys and NHS‐633 ester were allowed to stand for 20 min to ensure thorough interaction. Excess dyes were then removed using Zeba™ Dye and Biotin Removal Spin Columns and Filter Plates, which cleared unreacted dyes and other residues via centrifugation at 1000 rcf for 2 min. The obtained dye‐labeled Cys followed the same synthesis of Fe^3+^‐Cys‐ZIF‐8.

### Detection of thNOX‐like and OXD‐like Activity

Michaelis–Menten kinetic analysis of NOX‐like and OXD‐like activities was performed using HAc–NaAc buffer (0.2 m, pH 4.5) with 2 mg mL^−1^ samples. For NOX‐like activity, 50 µL of varying concentrations of NADH and 5 µL of samples were added, and the absorbance at 340 nm was measured over time using a CLARIOstar microplate reader (BMG LABTECH, Germany). A plot of absorbance at 340 nm versus time was generated to create a reaction‐time curve. For OXD‐like activity, the absorbance was monitored at 652 nm (ε = 39000 m ^−1^ cm^−1^). The initial rate of absorbance change was determined from the slope of the linear portion at the beginning of the curve. A Michaelis plot was then constructed by plotting the initial rate against substrate concentration, and the Michaelis–Menten equation was fitted using nonlinear least‐squares regression in GraphPad Prism (version 9) to calculate K_m_ and V_max_. The turnover number (k_cat_) was obtained by dividing V_max_ by the molar concentration of the catalyst. The Fe content in the samples was determined by inductively coupled plasma optical emission spectrometry (Optima 7000 and Avio, PerkinElmer, USA).

### Detection of the POD‐like and CAT‐like Activity

Michaelis–Menten kinetic analysis of POD‐like and CAT‐like activities was performed using HAc–NaAc buffer (0.2 m, pH 4.5) with 2 mg mL^−1^ SACs. To evaluate the catalytic production of hydroxyl radicals from H_2_O_2_ using terephthalic acid (TA), 50 µL of H_2_O_2_ (0.0979 m) and SACs containing the same amount of Fe single atoms were added, and the change in fluorescence intensity over time was measured (excitation at 320 nm, emission at 425 nm) using a CLARIOstar microplate reader (BMG LABTECH, Germany). For assessing the catalytic production of oxygen from H_2_O_2_ using Tris(2,2′‐bipyridine)ruthenium(II) hexafluorophosphate ([Ru(bpy)_3_]^2+^), the excitation and emission wavelengths were 450 and 625 nm, respectively.

### MTT Assay to Evaluate Cytotoxicity

To evaluate the ability of the prepared Fe‐SACs to suppress cancer cell growth and simulate the in vivo environment, 4T1 cells were selected for live/dead staining after treatment. The cells were cultured in 24‐well plates for 24 h, followed by the addition of different samples at varying concentrations, with or without 100 µm H_2_O_2_, for another 24 h. Cytotoxicity was assessed using the MTT assay. Specifically, 100 µL of thiazolyl blue tetrazolium bromide (0.5 mg mL^−1^) was added to each well. After incubation for 2 h at 37 °C, the supernatants were removed, 1 mL of DMSO was added, and the absorbance at 550 nm was measured using a Benchmark Plus microplate spectrophotometer (BMG LABTECH, Germany). Data are presented as the mean of three independent experiments. In parallel, a non‐tumorigenic control was conducted using 3T3 fibroblast cells following the identical protocols to assess off‐target cytotoxicity and comparative biocompatibility relative to 4T1 cells.

### Detection of Live and Dead Cells

Live and dead cells were stained with Calcein‐AM and PI, respectively. 4T1 cells were seeded into 24‐well plates and cultured for 24 h. The cells were then treated with different groups: i) Control, ii) 100 µm H_2_O_2_, iii) FeN_3_S, and iv) FeN_3_S + 100 µm H_2_O_2_. After 24 h of treatment, the cells were incubated with Calcein‐AM (2 µm) and PI (5 µg mL^−1^) for 20 min, and the fluorescence images of live and dead cells were captured using a fluorescence microscope.

### Detection of Cell Death States

Early apoptotic and late apoptotic cells were stained with V‐FITC, and necrotic cells were stained with PI. 4T1 cells were seeded into 24‐well plates and cultured for 24 h. The cells were then treated with or without 200 µg mL^−1^ FeN_3_S for 24 h. Then, cells were incubated with V‐FITC (5 µL) and PI (5 µg mL^−1^) for 10 min in the dark. The fluorescence images of apoptotic and necrotic cells were captured using a fluorescence microscope. The proportions of cells in different death states were quantified based on analysis of more than 10 random fields.

### Intracellular ROS Groups and ─O_2_O_2_^−^ Production

Intracellular ROS and ─O_2_^−^ generation were probed using DCFH‐DA and DHE, respectively. 4T1 cells were seeded into 18‐well plates and cultured for 24 h, followed by treatment with different groups: i) Control, ii) 100 µm H_2_O_2_, iii) FeN_3_S, and iv) FeN_3_S + 100 µm H_2_O_2_. After 24 h, the treated cells were incubated with DCFH‐DA (10 µm) or DHE (30 µm) for 20 min, and the ROS and ─O_2_^−^ production were visualized using a fluorescence microscope.

### Detection of Intracellular ATP Content

Intracellular ATP levels were determined using an ATP Determination Kit (A22066, Thermo Scientific). 4T1 cells were seeded in 24‐well plates and cultured for 24 h, followed by treatment with or without 200 µg mL^−1^ FeN_3_S for an additional 24 h. After treatment, 200 µL of CelLytic™ M reagent was added to each well to lyse the cells. The resulting lysates were centrifuged, and the supernatants were collected for ATP quantification using the ATP assay kit.

### Detection of Mitochondrial Membrane Potential (MMP)

The mitochondrial membrane potential was assessed using JC‐1 dye (Thermo Scientific). 4T1 cells were seeded in 24‐well plates and cultured for 24 h, followed by treatment with or without 200 µg mL^−1^ FeN_3_S for another 24 h. After treatment, the cells were incubated with JC‐1 dye (2 µm) for 20 min in the dark. Fluorescence images of green JC‐1 monomers (indicating low MMP) and red JC‐1 aggregates (indicating high MMP) were captured using a fluorescence microscope.

### Detection of Intracellular NADH Content

Intracellular NADH levels were measured using a NAD⁺/NADH Assay Kit (Sigma). 4T1 cells were seeded into culture bottles and cultured for 24 h, then treated with FeN_3_S for 24 h. After treatment, the cells were rinsed with PBS and collected by centrifugation at 3000 rpm. The cell pellets were suspended in 1 mL of NAD⁺/NADH extraction buffer, and the supernatants were collected for NADH quantification using the assay kit.

### Computational Details

All the DFT calculations were carried out in the Vienna ab initio simulation (VASP5.4.4) code.^[^ ^109^ ^]^ The exchange‐correlation was simulated with the PBE functional, and the ion‐electron interactions were described by the PAW method.^[^ ^110^, ^111^ ^]^ The vdWs interaction was included by using the empirical DFT‐D3 method.^[^ ^112^ ^]^ The Monkhorst‐Pack‐grid‐mesh‐based Brillouin zone k‐points were set as 2 × 2 × 1 for all periodic structures with the cutoff energy of 450 eV. The convergence criteria were set as ‐0.03 eV A^−1^ and 10^−5^ eV in force and energy, respectively.

### Statistical Analysis

All experiments were conducted in at least three independent biological replicates. Quantitative data were presented as mean ± SD. The exact sample size (n) for each analysis refers to the number of independent biological replicates and is provided in the figure legends. For two‐group comparisons, a two‐tailed unpaired Welch's *t*‐test (equal variances not assumed) was used. For multiple‐group (one‐factor) analyses, one‐way ANOVA followed by Šidák's multiple comparisons test was applied. For two‐factor datasets, two‐way ANOVA (mixed‐effects model with REML when appropriate) followed by Šidák's multiple comparisons test was used; interaction *P*‐values were reported where relevant. All tests were two‐sided with α = 0.05. Statistical significance was denoted as ns (p ≥ 0.05), ^*^ (p < 0.05), ^**^ (p < 0.01), ^***^ (p < 0.001), and ^****^ (p < 0.0001). For imaging‐based assays, each replicate used three wells per condition and ≥ 5 randomly selected fields per well. Field‐level measurements were background‐subtracted using identical thresholds across groups and averaged within wells; well means were then averaged to yield one value per biological replicate (statistical unit). Analyses were performed in GraphPad Prism 10 and Origin 2024. Data failing predefined quality‐control criteria (e.g., out‐of‐focus or saturated images) were excluded a priori before analysis. Where indicated, viability or ratio‐type readouts were normalized to the control condition (set to 100%).

## Conflict of Interest

The authors declare no conflict of interest.

## Supporting information



Supporting Information

## Data Availability

The data that support the findings of this study are available from the corresponding author upon reasonable request.

## References

[1] Y. Cao , X. Li , J. Ge , Trends Biotechnol. 2021, 39, 1173. 33551176 10.1016/j.tibtech.2021.01.002

[2] J. M. Choi , S. S. Han , H. S. Kim , Biotechnol. Adv. 2015, 33, 1443. 25747291 10.1016/j.biotechadv.2015.02.014

[3] S. Kim , S. Ga , H. Bae , R. Sluyter , K. Konstantinov , L. K. Shrestha , Y. H. Kim , J. H. Kim , K. Ariga , EES Catal. 2024, 2, 14.

[4] A. M. Ashrafi , Z. Bytesnikova , J. Barek , L. Richtera , V. Adam , Biosens. Bioelectron. 2021, 192, 113494. 34303137 10.1016/j.bios.2021.113494

[5] P. Sujitha , C. Shanthi , J. Clean. Prod. 2023, 425, 138915.

[6] W. He , J. Wu , J. Liu , J. Li , Adv. Funct. Mater. 2024, 34, 2312116.

[7] S. Ji , B. Jiang , H. Hao , Y. Chen , J. Dong , Y. Mao , Z. Zhang , R. Gao , W. Chen , R. Zhang , Nat. Catal. 2021, 4, 407.

[8] L. Jiao , H. Yan , Y. Wu , W. Gu , C. Zhu , D. Du , Y. Lin , Angew. Chem., Int. Ed. 2020, 59, 2565. 10.1002/anie.201905645 31209985

[9] M. Liu , W. Xu , Y. Tang , Y. Wu , W. Gu , D. Du , Y. Lin , C. Zhu , Angew. Chem., Int. Ed. 2025, 64, 202424070. 10.1002/anie.202424070 39937141

[10] W. Yang , X. Yang , L. Zhu , H. Chu , X. Li , W. Xu , Coord. Chem. Rev. 2021, 448, 214170.

[11] I. Zare , D. Choi , J. Zhang , M. T. Yaraki , A. Ghaee , S. Z. Nasab , R. Taheri‐Ledari , A. Maleki , A. Rahi , K. Fan , Nano Today 2024, 56, 102276.

[12] C. Cao , N. Yang , X. Wang , J. Shao , X. Song , C. Liang , W. Wang , X. Dong , Coord. Chem. Rev. 2023, 491, 215245.

[13] M. Ghafori‐Gorab , A. Kashtiaray , M. Karimi , H. A. M. Aliabadi , F. Bakhtiyar , F. D. Ghadikolaei , M. Mohajeri , A. Maleki , Chem. Eng. J. 2025, 505, 159464.

[14] Z. Wang , R. Zhang , X. Yan , K. Fan , Mater. Today. 2020, 41, 81.

[15] W. Xuan , X. Li , H. Gao , L. Zhang , J. Hu , L. Sun , H. Kan , Sci. Rep. 2025, 15, 13305. 40247044 10.1038/s41598-025-96815-9 PMC12006436

[16] H. Lv , W. Guo , M. Chen , H. Zhou , Y. Wu , Chin. J. Catal. 2022, 43, 71.

[17] S. Ding , J. A. Barr , Z. Lyu , F. Zhang , M. Wang , P. Tieu , X. Li , M. H. Engelhard , Z. Feng , S. P. Beckman , X. Pan , J.‐C. Li , D. Du , Y. Lin , Adv. Mater. 2024, 36, 2209633. 10.1002/adma.202209633 36722360

[18] E. M. Hamed , L. He , V. Rai , S. Hu , S. F. Y. Li , Small 2024, 20, 2405986. 10.1002/smll.202405986 39248675

[19] B. Xu , Y. Cui , W. Wang , S. Li , C. Lyu , S. Wang , W. Bao , H. Wang , M. Qin , Z. Liu , W. Wei , H. Liu , Adv. Mater. 2020, 32, 2003563. 10.1002/adma.202003563 32627937

[20] L. Wang , B. Wang , K. Kang , X. Ji , Y. Niu , C. Li , Y. Wang , Microchem. J. 2025, 215, 114182.

[21] E. M. Hamed , F. M. Fung , S. F. Y. Li , Small 2025, 21, 03879. 10.1002/smll.202503879 PMC12423899 40685687

[22] G. Chellasamy , E. Varathan , K. Sekar , S. Venkateswarlu , S. Govindaraju , K. Yun , Coord. Chem. Rev. 2024, 502, 215606.

[23] H. Xiang , W. Feng , Y. Chen , Adv. Mater. 2020, 32, 1905994. 10.1002/adma.201905994 31930751

[24] D. B. Tripathy , Inorg. Chem. Commun. 2025, 115245.

[25] W. Zhang , Y. Zhao , W. Huang , T. Huang , B. Wu , Coord. Chem. Rev. 2024, 515, 215952.

[26] Y. Zhang , J. Yang , R. Ge , J. Zhang , J. M. Cairney , Y. Li , M. Zhu , S. Li , W. Li , Coord. Chem. Rev. 2022, 461, 214493.

[27] M. Liu , T. Sun , T. Peng , J. Wu , J. Li , S. Chen , L. Zhang , S. Li , J. Zhang , S. Sun , ACS Energy Lett. 2023, 8, 4531.

[28] Y. Luo , K. Li , Y. Chen , J. Feng , L. Wang , Y. Jiang , L. Li , G. Yu , J. Feng , Adv. Mater. 2023, 35, 2300624. 10.1002/adma.202300624 37038691

[29] J. Liang , B. Johannessen , Z. Wu , R. F. Webster , J. Yong , M. Y. B. Zulkifli , J. S. Harbort , Y. R. Cheok , H. Wen , Z. Ao , B. Kong , S. Chang , J. Scott , K. Liang , Adv. Mater. 2022, 34, 2205674. 10.1002/adma.202205674 36073657

[30] J. Yang , M. Wang , S. Gao , M. Zhou , X. Du , L. Zhang , Y. Wang , X. Dai , Y. Jiang , Y. Li , Angew. Chem., Int. Ed. 2025, 64, 202504575; 10.1002/anie.202504575 40387625

[31] J. Zhou , D. Xu , G. Tian , Q. He , X. Zhang , J. Liao , L. Mei , L. Chen , L. Gao , L. Zhao , J. Am. Chem. Soc. 2023, 145, 4279. 10.1021/jacs.2c13597 36744911

[32] L. Jiao , W. Xu , Y. Zhang , Y. Wu , W. Gu , X. Ge , B. Chen , C. Zhu , S. Guo , Nano Today 2020, 35, 100971.

[33] J. Li , Z. Yang , Y. Li , G. Zhang , J. Hazard. Mater. 2022, 429, 128285. 35093746 10.1016/j.jhazmat.2022.128285

[34] B. Xu , H. Wang , W. Wang , L. Gao , S. Li , X. Pan , H. Wang , H. Yang , X. Meng , Q. Wu , Angew. Chem. 2019, 131, 4965.

[35] Y. Chen , S. Ji , C. Chen , Q. Peng , D. Wang , Y. Li , Joule 2018, 2, 1242.

[36] S. Swain , A. Altaee , M. Saxena , A. K. Samal , Coord. Chem. Rev. 2022, 470, 214710.

[37] T. Zhang , Z. Chen , A. G. Walsh , Y. Li , P. Zhang , Adv. Mater. 2020, 32, 2002910. 10.1002/adma.202002910 32656812

[38] Q. Li , W. Chen , H. Xiao , Y. Gong , Z. Li , L. Zheng , X. Zheng , W. Yan , W.‐C. Cheong , R. Shen , N. Fu , L. Gu , Z. Zhuang , C. Chen , D. Wang , Q. Peng , J. Li , Y. Li , Adv. Mater. 2018, 30, 1800588. 10.1002/adma.201800588 29726038

[39] Y. Chen , S. Ji , S. Zhao , W. Chen , J. Dong , W.‐C. Cheong , R. Shen , X. Wen , L. Zheng , A. I. Rykov , S. Cai , H. Tang , Z. Zhuang , C. Chen , Q. Peng , D. Wang , Y. Li , Nat. Commun. 2018, 9, 5422. 30575726 10.1038/s41467-018-07850-2 PMC6303331

[40] Z. Chen , H. Niu , J. Ding , H. Liu , P.‐H. Chen , Y.‐H. Lu , Y.‐R. Lu , W. Zuo , L. Han , Y. Guo , S.‐F. Hung , Y. Zhai , Angew. Chem., Int. Ed. 2021, 60, 25404. 10.1002/anie.202110243 34550627

[41] P. Muhammad , A. Zada , J. Rashid , S. Hanif , Y. Gao , C. Li , Y. Li , K. Fan , Y. Wang , Adv. Funct. Mater. 2024, 34, 2314686.

[42] J. Li , X. Cai , P. Jiang , H. Wang , S. Zhang , T. Sun , C. Chen , K. Fan , Adv. Mater. 2024, 36, 2307337; 10.1002/adma.202307337 37724878

[43] E. M. Hamed , V. Rai , S. F. Y. Li , Chemosphere 2024, 346, 140557. 38303399 10.1016/j.chemosphere.2023.140557

[44] E. M. Hamed , F. M. Fung , S. F. Y. Li , ACS Sens. 2024, 9, 3840. 39083641 10.1021/acssensors.4c00630

[45] C. Peng , R. Pang , J. Li , E. Wang , Adv. Mater. 2024, 36, 2211724. 10.1002/adma.202211724 36773312

[46] B. Xu , R. Niu , R. Deng , Y. Tang , C. Wang , Y. Wang , Adv. Funct. Mater. 2024, 34, 2405265.

[47] Y. Liu , B. Wang , J. Zhu , X. Xu , B. Zhou , Y. Yang , Adv. Mater. 2023, 35, 2208512. 10.1002/adma.202208512 36373624

[48] E. A. Peroza , R. Schmucki , P. Güntert , E. Freisinger , O. Zerbe , J. Mol. Biol. 2009, 387, 207. 19361445 10.1016/j.jmb.2009.01.035

[49] G. Chen , S. Huang , X. Kou , S. Wei , S. Huang , S. Jiang , J. Shen , F. Zhu , G. Ouyang , Angew. Chem., Int. Ed. 2019, 58, 1463. 10.1002/anie.201813060 30536782

[50] M. Lukács , D. Csilla Pálinkás , G. Szunyog , K. Várnagy , ChemistryOpen 2021, 10, 451. 33830669 10.1002/open.202000304 PMC8028610

[51] R. F. Jameson , W. Linert , A. Tschinkowitz , V. Gutmann , J. Chem. Soc., Dalton Trans. 1988, 10.1039/DT9880000943.

[52] J. Liang , M. Y. Bin Zulkifli , J. Yong , Z. Du , Z. Ao , A. Rawal , J. A. Scott , J. R. Harmer , J. Wang , K. Liang , J. Am. Chem. Soc. 2022, 144, 17865. 36075889 10.1021/jacs.2c06471

[53] A. Huang , Z.‐W. Li , L. Guo , N. Zhong , L. Tong , Y. Xu , X. Ma , F. Zhu , G. Chen , S. Huang , G. Ouyang , Nat. Commun. 2025, 16, 4660. 40389411 10.1038/s41467-025-59824-w PMC12089486

[54] K. Liang , R. Ricco , C. M. Doherty , M. J. Styles , P. Falcaro , CrystEngComm 2016, 18, 4264.

[55] K. Liang , R. Ricco , C. M. Doherty , M. J. Styles , S. Bell , N. Kirby , S. Mudie , D. Haylock , A. J. Hill , C. J. Doonan , P. Falcaro , Nat. Commun. 2015, 6, 7240. 26041070 10.1038/ncomms8240 PMC4468859

[56] S. Devi , B. Singh , A. Paul , S. Tyagi , Anal. Methods. 2016, 8, 4398.

[57] C. Avci , I. Imaz , A. Carné‐Sánchez , J. A. Pariente , N. Tasios , J. Pérez‐Carvajal , M. I. Alonso , A. Blanco , M. Dijkstra , C. López , D. Maspoch , Nat. Chem. 2018, 10, 78. 10.1038/nchem.2875 29256498

[58] B. Liu , H. Shioyama , T. Akita , Q. Xu , J. Am. Chem. Soc. 2008, 130, 5390. 18376833 10.1021/ja7106146

[59] R. Boppella , M. A. P. Austeria , Y. Kim , E. Kim , I. Song , Y. Eom , D. P. Kumar , M. Balamurugan , E. Sim , D. H. Kim , Adv. Funct. Mater. 2022, 32, 2202351.

[60] M. Chang , Z. Hou , M. Wang , C. Yang , R. Wang , F. Li , D. Liu , T. Peng , C. Li , J. Lin , Angew. Chem., Int. Ed. 2021, 60, 12971. 10.1002/anie.202101924 33772996

[61] H. Zhang , S. Hwang , M. Wang , Z. Feng , S. Karakalos , L. Luo , Z. Qiao , X. Xie , C. Wang , D. Su , J. Am. Chem. Soc. 2017, 139, 14143. 28901758 10.1021/jacs.7b06514

[62] Y. Wang , G. Jia , X. Cui , X. Zhao , Q. Zhang , L. Gu , L. Zheng , L. H. Li , Q. Wu , D. J. Singh , Chem 2021, 7, 436.

[63] Y. Pan , Y. Chen , K. Wu , Z. Chen , S. Liu , X. Cao , W.‐C. Cheong , T. Meng , J. Luo , L. Zheng , Nat. Commun. 2019, 10, 4290. 31537799 10.1038/s41467-019-12362-8 PMC6753116

[64] Y. Cao , Y. Liu , X. Zheng , J. Yang , H. Wang , J. Zhang , X. Han , Y. Deng , G. Rupprechter , W. Hu , Angew. Chem., Int. Ed. 2025, 64, 202423556. 10.1002/anie.202423556 39844730

[65] P. Chen , N. Zhang , T. Zhou , Y. Tong , W. Yan , W. Chu , C. Wu , Y. Xie , ACS Mater. Lett. 2019, 1, 139.

[66] Y. Long , Z. Cao , W. Wu , W. Liu , P. Yang , X. Zhan , R. Chen , D. Liu , W. Huang , Appl. Catal. B‐Environ. 2024, 344, 123643.

[67] L. Zhang , J. Feng , L. Wu , X. Ma , X. Song , S. Jia , X. Tan , X. Jin , Q. Zhu , X. Kang , J. Am. Chem. Soc. 2023, 145, 21945. 37751566 10.1021/jacs.3c06697

[68] W. Wang , W. Zhou , Y. Tang , W. Cao , S. R. Docherty , F. Wu , K. Cheng , Q. Zhang , C. Copéret , Y. Wang , J. Am. Chem. Soc. 2023, 145, 12928. 37267262 10.1021/jacs.3c04260

[69] P. Zhang , J. Guo , Y. Wang , W. Pang , Mater. Lett. 2002, 53, 400.

[70] L. Jiao , J. Wu , H. Zhong , Y. Zhang , W. Xu , Y. Wu , Y. Chen , H. Yan , Q. Zhang , W. Gu , ACS Catal. 2020, 10, 6422.

[71] T. Wang , X. Sang , W. Zheng , B. Yang , S. Yao , C. Lei , Z. Li , Q. He , J. Lu , L. Lei , Adv. Mater. 2020, 32, 2002430. 10.1002/adma.202002430 32538500

[72] C. Zhu , F. Cun , Y. Nie , Q. Du , F. Lao , C. Yue , F. Liu , A. Li , Appl. Catal. B. 2024, 358, 124409.

[73] Y. Gao , S. Liang , B. Liu , C. Jiang , C. Xu , X. Zhang , P. Liang , M. Elimelech , X. Huang , Nat. Commun. 2023, 14, 2059. 37045829 10.1038/s41467-023-37676-6 PMC10097648

[74] T. Wang , X. Sang , W. Zheng , B. Yang , S. Yao , C. Lei , Z. Li , Q. He , J. Lu , L. Lei , L. Dai , Y. Hou , Adv. Mater. 2020, 32, 2002430. 10.1002/adma.202002430 32538500

[75] S. Gao , T. Wei , J. Sun , Q. Liu , D. Ma , W. Liu , S. Zhang , J. Luo , X. Liu , Small Struct. 2022, 3, 2200086.

[76] L. Jiao , Y. Kang , Y. Chen , N. Wu , Y. Wu , W. Xu , X. Wei , H. Wang , W. Gu , L. Zheng , W. Song , C. Zhu , Nano Today 2021, 40, 101261.

[77] W. Liu , Q. Chen , J. Wu , F. Zhang , L. Han , J. Liu , H. Zhang , Z. Hao , E. Shi , Y. Sun , R. Zhang , Y. Wang , L. Zhang , Adv. Funct. Mater. 2024, 34, 2312308.

[78] A. Walsh , A. A. Sokol , J. Buckeridge , D. O. Scanlon , C. R. A. Catlow , Nat. Mater. 2018, 17, 958; 30275565 10.1038/s41563-018-0165-7

[79] N. Daelman , M. Capdevila‐Cortada , N. López , Nat. Mater. 2019, 18, 1215. 31384029 10.1038/s41563-019-0444-y

[80] L. Yang , R. Liu , L. Jiao , Adv. Funct. Mater. 2020, 30, 1909618.

[81] L. Huang , J. Chen , L. Gan , J. Wang , S. Dong , Sci. Adv. 2019, 5, aav5490. 10.1126/sciadv.aav5490 PMC6499548 31058221

[82] B. Xu , S. Li , L. Zheng , Y. Liu , A. Han , J. Zhang , Z. Huang , H. Xie , K. Fan , L. Gao , Adv. Mater. 2022, 34, 2107088. 10.1002/adma.202107088 35102632

[83] L. Liu , X. Sun , Y. Li , X.‐D. Zhang , ACS Omega 2024, 9, 35144. 39157134 10.1021/acsomega.4c04990 PMC11325499

[84] X. Lu , S. Gao , H. Lin , L. Yu , Y. Han , P. Zhu , W. Bao , H. Yao , Y. Chen , J. Shi , Adv. Mater. 2020, 32, 2002246. 10.1002/adma.202002246 32705751

[85] W. Qu , S. Tang , Z. Tang , T. Zhong , H. Zhao , S. Tian , D. Shu , C. He , Adv. Funct. Mater. 2024, 34, 2314187.

[86] X. Luo , W. Wei , Y. Xu , D. Liu , Z. Wei , J. Liu , Z. Li , L. Wang , S. Ouyang , H. Yuan , Z. Liu , T. Zhang , Angew. Chem., Int. Ed. 2025, 64, 202502430. 10.1002/anie.202502430 40214211

[87] N. Wang , C. Meng , B. Wang , X. Tan , Y. Wan , Y. Yang , D. Kong , W. Wang , F. Cao , A. J. Fielding , L. Li , M. Wu , H. Hu , Natl. Sci. Rev. 2025, 12, nwaf061. 40511371 10.1093/nsr/nwaf061 PMC12153717

[88] P. Kowalczyk , P. A. Gauden , S. Furmaniak , A. P. Terzyk , M. Wiśniewski , A. Ilnicka , J. Łukaszewicz , A. Burian , J. Włoch , A. V. Neimark , Carbon 2017, 111, 358.

[89] J. Liang , S. Gao , J. Liu , M. Y. B. Zulkifli , J. Xu , J. Scott , V. Chen , J. Shi , A. Rawal , K. Liang , Angew. Chem., Int. Ed. 2021, 60, 5421. 10.1002/anie.202014002 33258208

[90] R. Chenitz , U. I. Kramm , M. Lefèvre , V. Glibin , G. Zhang , S. Sun , J.‐P. Dodelet , Energy. Environ. Sci. 2018, 11, 365.

[91] W. Zhang , X. Jiang , X. Wang , Y. V. Kaneti , Y. Chen , J. Liu , J. S. Jiang , Y. Yamauchi , M. Hu , Angew. Chem., Int. Ed. 2017, 56, 8435. 10.1002/anie.201701252 28382724

[92] J. Sui , H. Liu , S. Hu , K. Sun , G. Wan , H. Zhou , X. Zheng , H.‐L. Jiang , Adv. Mater. 2022, 34, 2109203. 10.1002/adma.202109203 34883530

[93] L. Yang , N. Huang , C. Luo , H. Yu , P. Sun , X. Lv , X. Sun , Chem. Eng. J. 2021, 404, 127112.

[94] Y. Chen , B. Jiang , H. Hao , H. Li , C. Qiu , X. Liang , Q. Qu , Z. Zhang , R. Gao , D. Duan , S. Ji , D. Wang , M. Liang , Angew. Chem. 2023, 62, 202301879. 10.1002/anie.202301879 36872618

[95] R. Niu , Y. Liu , B. Xu , R. Deng , S. Zhou , Y. Cao , W. Li , H. Zhang , H. Zheng , S. Song , Y. Wang , H. Zhang , Adv. Mater. 2024, 36, 2312124. 10.1002/adma.202312124 38314930

[96] R. Zhang , B. Xue , Y. Tao , H. Zhao , Z. Zhang , X. Wang , X. Zhou , B. Jiang , Z. Yang , X. Yan , K. Fan , Adv. Mater. 2022, 34, 2205324. 10.1002/adma.202205324 35953446

[97] D. Wang , J. Wang , X. J. Gao , H. Ding , M. Yang , Z. He , J. Xie , Z. Zhang , H. Huang , G. Nie , Adv. Mater. 2024, 36, 2310033. 10.1002/adma.202310033 37994246

[98] X. Lu , S. Gao , H. Lin , L. Yu , Y. Han , P. Zhu , W. Bao , H. Yao , Y. Chen , J. Shi , Adv. Mater. 2020, 32, 2002246. 10.1002/adma.202002246 32705751

[99] X. Wang , Q. Chen , Y. Zhu , K. Wang , Y. Chang , X. Wu , W. Bao , T. Cao , H. Chen , Y. Zhang , Signal Transduct. Target. Ther. 2023, 8, 277. 37474504 10.1038/s41392-023-01491-8 PMC10359331

[100] M. Wang , C. Yang , M. Chang , Y. Xie , G. Zhu , Y. Qian , P. Zheng , Q. Sun , J. Lin , C. Li , Nano Today 2023, 52, 101981.

[101] S. Zhong , C. Xiong , Y. Zhao , S. Yao , Q. Hu , S. Wang , Q. Zhao , L. Li , Adv. Funct. Mater. 2023, 33, 2305625.

[102] M. Huo , L. Wang , Y. Wang , Y. Chen , J. Shi , ACS Nano 2019, 13, 2643. 30753056 10.1021/acsnano.9b00457

[103] M. Debayle , E. Balloul , F. Dembele , X. Xu , M. Hanafi , F. Ribot , C. Monzel , M. Coppey , A. Fragola , M. Dahan , T. Pons , N. Lequeux , Biomater 2019, 219, 119357. 10.1016/j.biomaterials.2019.119357 31351245

[104] Q. He , R. Li , J. Liu , Z. Wu , L. Liu , B. Xu , L. Zhang , Adv. Healthcare Mater. 2025, 14, 2500207.

[105] Q. Zhang , J. Liang , A. Bongers , J. J. Richardson , K. Liang , Z. Gu , Adv. Sci. 2023, 10, 2206546. 10.1002/advs.202206546 PMC10037962 36698301

[106] B. Ravel , M. Newville , J. Synchrotron. Radiat. 2005, 12, 537. 15968136 10.1107/S0909049505012719

[107] S. I. Zabinsky , J. J. Rehr , A. Ankudinov , R. C. Albers , M. J. Eller , Phys. Rev. B 1995, 52, 2995. 10.1103/physrevb.52.2995 9981373

[108] H. Funke , A. C. Scheinost , M. Chukalina , Phys. Rev. 2005, 71, 094110.

[109] G. Kresse , J. Furthmuller , Phys. Rev. B 1996, 54, 11169. 10.1103/physrevb.54.11169 9984901

[110] J. P. Perdew , K. Burke , M. Ernzerhof , Phys. Rev. Lett. 1996, 77, 3865. 10062328 10.1103/PhysRevLett.77.3865

[111] B. Hammer , L. B. Hansen , J. K. Norskov , Phys. Rev. B 1999, 59, 7413.

[112] S. Grimme , J. Comput. Chem. 2006, 27, 1787. 16955487 10.1002/jcc.20495

