# Supplementary material for: Metallothionein‐Inspired Asymmetric Heteroatom Doping of Single‐Atom Nanozymes for Multi‐Enzyme Biocatalysis
Source: Adv Sci (Weinh). 2025 Nov 3;13(5):e17502. doi: 10.1002/advs.202517502 (PMC12850297; doi:10.1002/advs.202517502)
Supplement: Supplementary file 1 — Supporting Information [file ADVS-13-e17502-s001.docx]

Supporting Information

Metallothionein-inspired Asymmetric Heteroatom Doping of Single-Atom Nanozymes for Multi-Enzyme Biocatalysis

Kaijuan Chen^a^, Qianfan Chen^b^, Bernt Johannessen^c^, Chun-Ho Lin^d^, Long Hu^d^, Kang Liang^a,b^*, Jieying Liang^a^*

K. Chen, K. Liang, J. Liang

School of Chemical Engineering, Australian Centre for NanoMedicine, The University of New South Wales, Sydney, NSW 2052, Australia

E-mail: kang.liang@unsw.edu.au, jieying.liang@unsw.edu.au

Q. Chen, K. Liang

Graduate School of Biomedical Engineering, The University of New South Wales, Sydney, NSW 2052, Australia

B. Johannessen

Australian Synchrotron, ANSTO, Clayton, Victoria, 3168 Australia

C. Lin, L. Hu

School of Materials Science and Engineering, University of New South Wales (UNSW), 2052, Sydney, NSW, Australia

**Table S1.** Elemental quantification of different samples determined by XPS.

| Samples | C / at% | O / at% | N / at% | Zn / at% |
| --- | --- | --- | --- | --- |
| ZIF-8 | 63.76 | 2.94 | 26.4 | 6.89 |
| Cys-ZIF-8 | 62.76 | 2.45 | 27.54 | 7.26 |
| Fe^3+^-ZIF-8 | 67.05 | 2.86 | 23.92 | 6.17 |
| Fe^3+^-Cys-ZIF-8 | 64.51 | 3.06 | 25.92 | 6.52 |
| NC | 81.2 | 4.65 | 12.18 | 1.96 |
| SNC | 80.6 | 5.00 | 12.41 | 1.98 |
| FeN_3_ | 73.45 | 6.74 | 16.68 | 3.04 |
| FeN_3_S | 81.06 | 4.35 | 12.67 | 1.91 |
| NC-900 | 85.5 | 5.96 | 7.35 | 1.18 |
| SNC-900 | 86.59 | 5.52 | 6.71 | 1.18 |
| FeN_3_-900 | 85.37 | 4.11 | 9.36 | 1.06 |
| FeN_3_S -900 | 87.31 | 3.95 | 7.84 | 0.84 |

**Table S2.** Metal elements concentration of each sample analyzed by ICP.

| Samples | Zn (mM/kg) | Fe (mM/kg) |
| --- | --- | --- |
| NC | 1747.40 | 2.20 |
| NC-900 | 1525.27 | 0.00 |
| SNC | 1536.68 | 0.90 |
| SNC-900 | 1510.45 | 0.00 |
| FeN_3_ | 1729.63 | 161.25 |
| FeN_3_-900 | 636.17 | 165.73 |
| FeN_3_S | 1795.12 | 164.13 |
| FeN_3_S-900 | 590.50 | 176.11 |
| Fe SAC (1,2-echanedithiol) | 3586.72 | 26.86 |
| Fe SAC (1-butanethiol) | 3211.99 | 26.86 |
| Fe SAC (thioglycolic acid) | 3455.19 | 26.86 |

**Table S3.** Linear combination fitting result of the XANES data.

| Samples | Fe_2_O_3_ oxidation state (%) | FePc oxidation state (%) | Fe foil oxidation state (%) | R-factor | Reduced chi-square value |
| --- | --- | --- | --- | --- | --- |
| FeN_3_ | 19.6±3.4 | 70.4±3.6 | 10±1 | 0.0024614 | 0.0005439 |
| FeN_3_S | 24.5±3.1 | 72.6±3.2 | 2.9±0.9 | 0.0019098 | 0.0004348 |
| FeN_3_-900 | 16.1±3.8 | 76.3 ± 4 | 7.6 ±1.1 | 0.0030001 | 0.0006762 |
| FeN_3_S-900 | 15±4.1 | 77.2 ± 4.3 | 7.8 ± 1.2 | 0.0037363 | 0.0007983 |

**Table S4.** EXAFS data fitting results of Samples.

| Sample | Path | *CN^a^* | *R*(Å)*^b^* | *σ*^2^ (Å^2^)*^c^* | Δ*E*_0_(eV)*^d^* | *R* factor |
| --- | --- | --- | --- | --- | --- | --- |
| Fe K-edge (*Ѕ*_0_^2^=0.774) | | | | | | |
| Fe foil | Fe-Fe | 8.0* | 2.465±0.010 | 0.0047 | 5.7±1.6 | 0.0026 |
|  | Fe-Fe | 6.0* | 2.848±0.009 | 0.0052 |  |  |
| FeN_3_ | Fe-N | 3.4±0.2 | 2.008±0.011 | 0.0113 | 0.1±1.7 | 0.0105 |
| FeN_3_S | Fe-N | 3.3±0.4 | 1.997±0.030 | 0.0123 | -4.2±4.5 | 0.0188 |
|  | Fe-S | 1.0±0.3 | 2.185±0.023 |  |  |  |
| FeN_3_-900 | Fe-N | 2.9±0.3 | 1.998±0.017 | 0.0074 | -1.9±3.5 | 0.0193 |
| FeN_3_S-900 | Fe-N | 2.9±0.3 | 1.960±0.021 | 0.0112 | -7.7±3.4 | 0.0081 |
|  | Fe-S | 1.1±0.5 | 2.217±0.027 |  |  |  |

*^a^CN*, coordination number; *^b^R*, the distance between absorber and backscatter atoms; *^c^σ*^2^, the Debye Waller factor value; *^d^ΔE*_0_, inner potential correction to account for the difference in the inner potential between the sample and the reference compound; *R* factor indicates the goodness of the fit. *S*0^2^ was fixed to 0.774, according to the experimental EXAFS fit of Fe foil by fixing *CN* as the known crystallographic value. * This value was fixed during EXAFS fitting, based on the known structure of Fe. Fitting conditions: *k* range：2.6 - 12.0; *R* range: 1.0-2.5; fitting space: R space; *k*-weight = 3. A reasonable range of EXAFS fitting parameters: 0.800 < *Ѕ*_0_^2^ < 1.000; *CN >* 0; *σ*^2^ > 0 Å^2^; |Δ*E*_0_| < 15 eV; *R* factor < 0.02.

**Table S5.** BET surface area, pore size and total pore volume of samples analyzed from N_2_ adsorption–desorption isotherms.

| Samples | BET surface (m^2^/g) | Pore size (nm) | Total pore volume (cm^3^/g) |
| --- | --- | --- | --- |
| NC | 1627.9 | 0.93 | 0.63 |
| NC-900 | 139.1 | 3~10 | 0.20 |
| SNC | 1447.6 | 0.86 | 0.71 |
| SNC-900 | 764.7 | 1.00, 1.09 | 0.38 |
| FeN_3_ | 1117.7 | 1.00; | 0.58 |
| FeN_3_S-900 | 387.91 | 1.27; 3~10 | 0.29 |
| FeN_3_S | 831.6 | 1.18 | 0.53 |
| FeN_3_S-900 | 355.1 | 1.36; 3~10 | 0.37 |

| Asymmetric SAC | Support | Hetero atom-containing chemical | Metal precursors | Enzyme-like activity | Unique | Ref |
| --- | --- | --- | --- | --- | --- | --- |
| FeN_3_S | MOF-based carbon | Cysteine (S) | FeCl_3_ | NOX, OXD, POD, CAT | (i) atomically dispersed Fe from biomineralization; (ii) the large surface area and pore volume retained from the original MOF, and (iii) the S-doping achieved through the strong mercaptide coordination between Fe and S. | This work |
| FeN_3_S | glucosamine, dicyandiamide | Thiourea (S) | FeCl_2_ | POD | Colloidal silica was used as a template to construct hierarchically porous nanostructures. | ^[1]^ |
| FeN_3_S | MOF-based carbon | trithiocyanuric acid (TCA) (S) | Hemin | POD, glutathione oxidase (GSHOx) | Strong coordination interaction between sulfhydryl in TCA and Zn^2+^/Fe^2+^ | ^[2]^ |
| Fe-N_3_P | MOF-based carbon | poly-(cyclotriphospazene-co-4,4’-diaminodiphenylether) (P) | Fe(NO_3_)_3_·9H_2_O | POD | Modulating the single-atom Fe through the precise coordination of P and N | ^[3]^ |
| Co-N_3_PS | MOF-based hollow carbon | phosphonitrilic chloride trimer (P), bis(4-hydroxyphenyl) sulfone (S) | Co(NO_3_)_2_·6H_2_O | CAT | Developed an ordered structure-oriented coordination design strategy via DFT screening. | ^[4]^ |
| Cu-N_3_S | ZnS | Glutathione | CuCl_2_·2H_2_O | POD, NOx, L-cysteine oxidase (LCO), GSHOx | Cu-N_3_S_1_ loaded with pyruvate oxidase and the mitochondrial pyruvate carrier inhibitor UK5099 as highly efficient initiators. | ^[5]^ |

**Table S6**. Comparison of reported asymmetric SAC designs with the present work.

**Table S7.** The release of Fe, Zn and S of FeN_3_S at different time points.

| Time (h) | The release of Fe ions (ng/ml) | The release of Zn ions (ng/ml) | The release of S (ng/ml) |
| --- | --- | --- | --- |
| 24 | 224 (12.2%) | 4620 (19.7%) | / |
| 72 | 234 (12.7%) | 8450 (36%) | 4000 |

**Table S8.** Percentage of Annexin V-FITC and PI-stained 4T1 cells after 24 h incubation with FeN_3_S (200 µg/mL), determined from 10 fluorescence microscopy images and corroborated by MTT assay results.

| **V-FITC** | **PI** | **%** |
| --- | --- | --- |
| + | + | 41.5 |
| + | - | 25 |
| - | + | 1 |
| - | - | 32.5 |

**Table S9.** Reported SACs for tumor cell suppression.

| SACs | Synthesis | Enzymatic activity type | Performance | Reference |
| --- | --- | --- | --- | --- |
| FeN_3_S | Metallothionein-inspired asymmetric heteroatom doping of Fe SAC | OXD, NOX, POD, CAT | ~ 90% mortality of 4T1 cells after 24 h with 300 μg/mL FeN_3_S. | This work |
| Cu-N_4_ | One-step fabricated and then pyrolysis at 900 °C for 3 h under nitrogen | POD, CAT, OXD and GSHOx | ~ 78% mortality of 4T1 cells after 12 h with 200 μg/mL SAC. | ^[6]^ |
| Noble‑metal (e.g., Pt/Pd)-N_4_ | Metal–porphyrin coordination strategy | POD | ~70% mortality of CNE1^LMP1^ cells after 24 h with 200 μg/mL SAC. | ^[7]^ |
| Cu-N_4_ | Using a silica hard template, a Cu–ligand polymer shell is assembled via chelation and quaternization. Pyrolyzed at 800 °C followe by NH_4_HF_2_ etching | OXD, POD | ~ 63% mortality of 4T1 cells after 24 h with addition of 100 μM H_2_O_2_ with 200 μg/mL SAC. | ^[8]^ |
| Fe-N_5_ | Fe-doped ZIF-8 was SiO₂-coated, calcined at 900 °C follow by NaOH-etching | OXD, POD, GSHOx | ~ 59% mortality of 4T1 cells after 24 h with 150 μg/mL SAC. | ^[9]^ |
| Ir-N_5_ | In situ capture Ir(acac)_3_ during ZIF-8 crystallization, calcined at 900 °C under N₂ flow for 3 h with addition of melamine. | CAT, POD. OXD, NOX | ~ 60% and 87% mortality of 4T1 cells after 24 h without/with addition of 25 μg/mL cerulenin and 100 μg/mL SAC. | ^[10]^ |
| FeN_4_ | Ferritin was encapsulated in ZIF-8 via biomineralization and then pyrolyzed t 800 °C under N₂ flow for 2 h. | POD | ~ 56% mortality of 4T1 cells after 10 h with 100 μg/mL SAC. | ^[11]^ |
| BSA-Cu | BSA solution was complexed with CuCl₂, basified to pH 12, and heated at 78 °C for 30 min. Ascorbic acid was then added and the mixture held at 78 °C for 1 h. | POD, OXD, GSHOx | ~66.2% mortality of HCT116 cells after 24 h with addition of 100 μM H_2_O_2_ with 60 μg/mL SAC. | ^[12]^ |


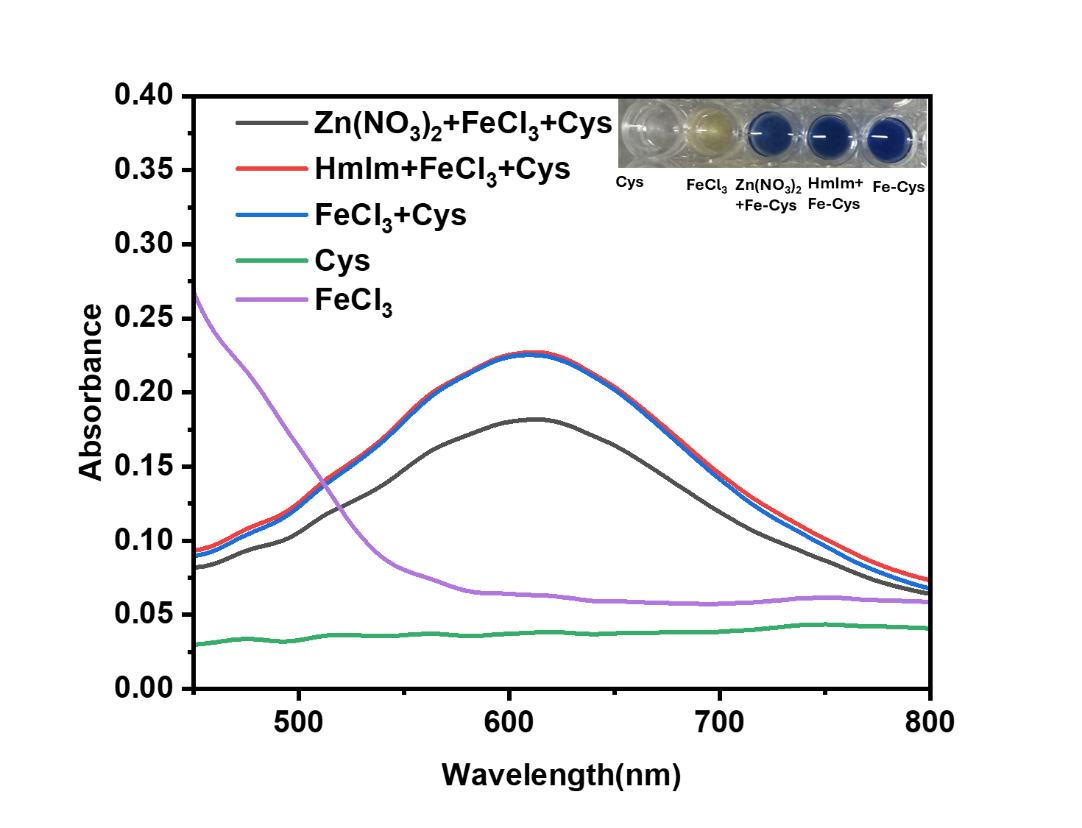


**Figure S1**. Comparison of UV–vis spectra of Cys, FeCl_3_, Fe–Cys complex, and Fe–Cys complex upon addition of HmIm and Zn^2^⁺, respectively.

**Figure S2**. FTIR spectra of Cys and Fe^3+^-Cys.


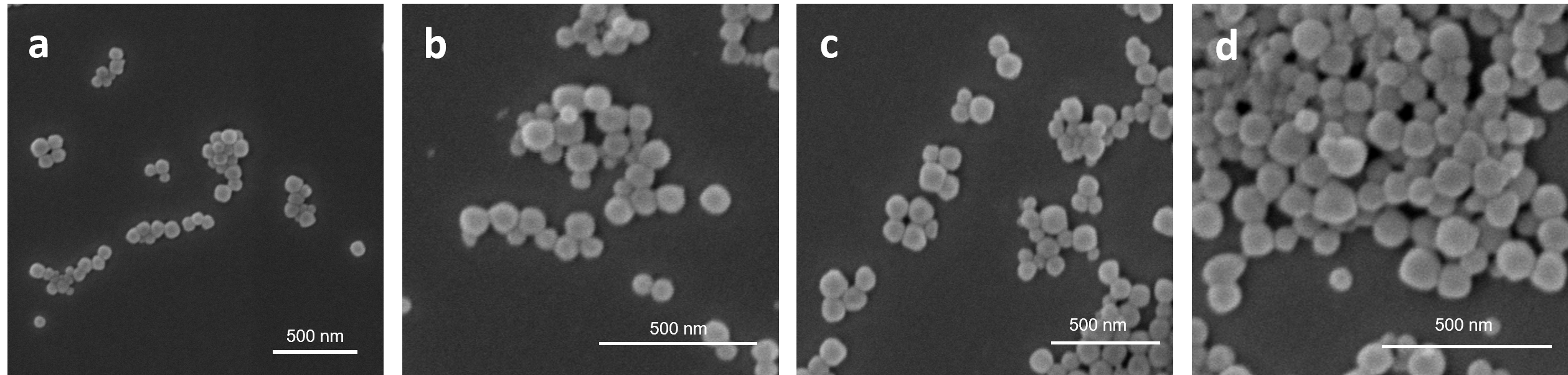


**Figure S3**. SEM images of (a) ZIF-8; (b) Cys-ZIF-8; (c) Fe^3+^-ZIF-8 and (d) Fe^3+^-Cys-ZIF-8.

**Figure S4**. PXRD patterns of ZIF-8, Cys-ZIF-8, Fe^3+^-ZIF-8 and Fe^3+^-Cys-ZIF-8.


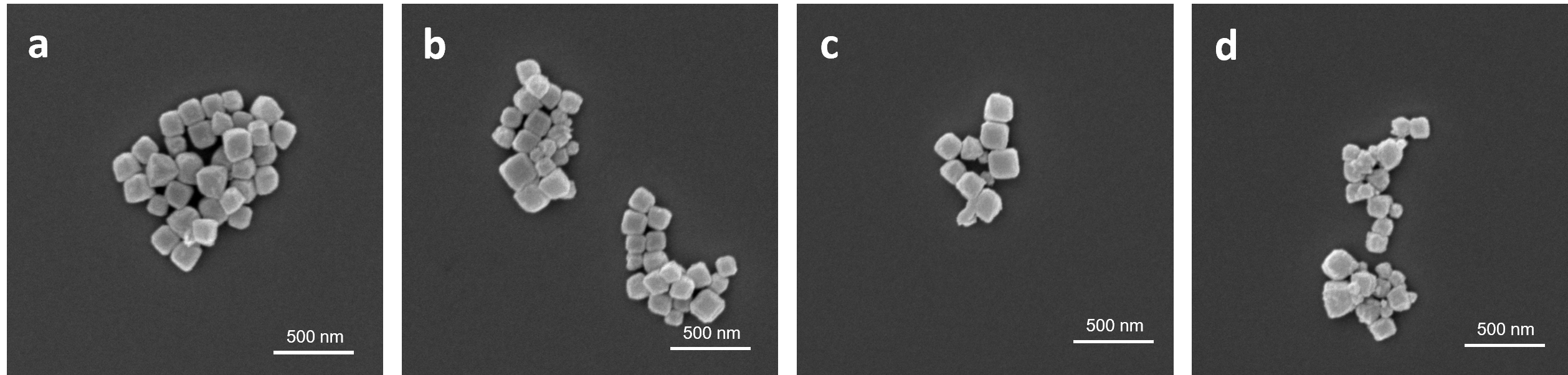


**Figure S5**. SEM images of (a) NC; (b) SNC; (c) FeN_3_ and (d) FeN_3_S.


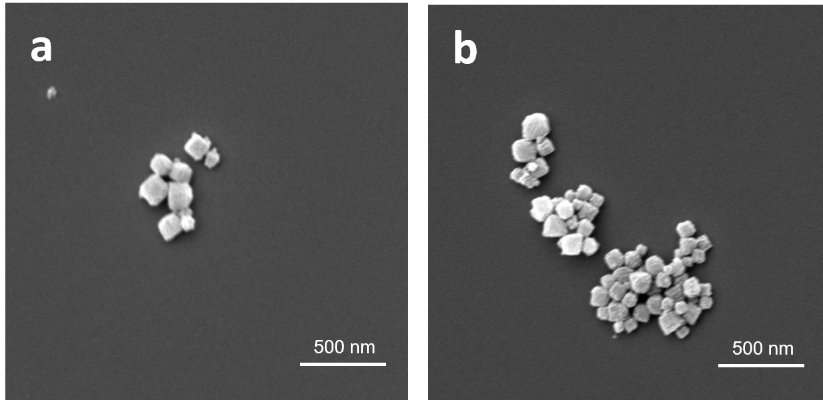


**Figure S6**. SEM images of (a) FeN_3_-900 and (b) FeN_3_S-900.


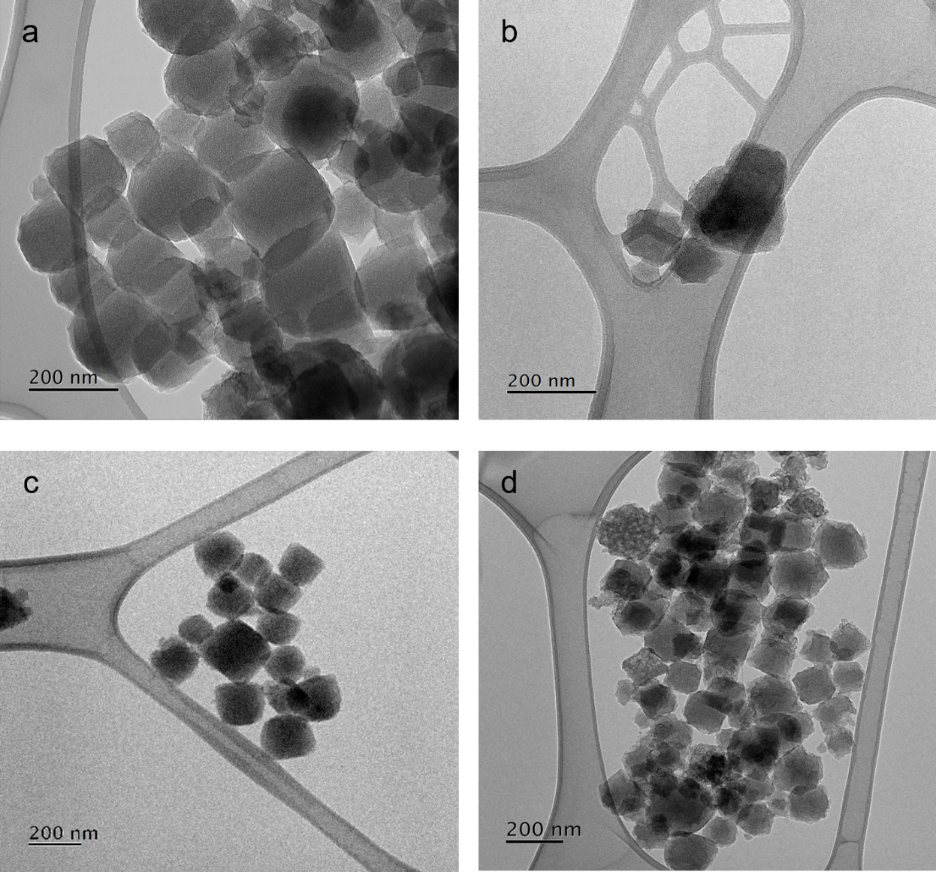


**Figure S7**. TEM images of (a) FeN_3_; (b) FeN_3_-900; (c) FeN_3_S and (d) FeN_3_S -900.


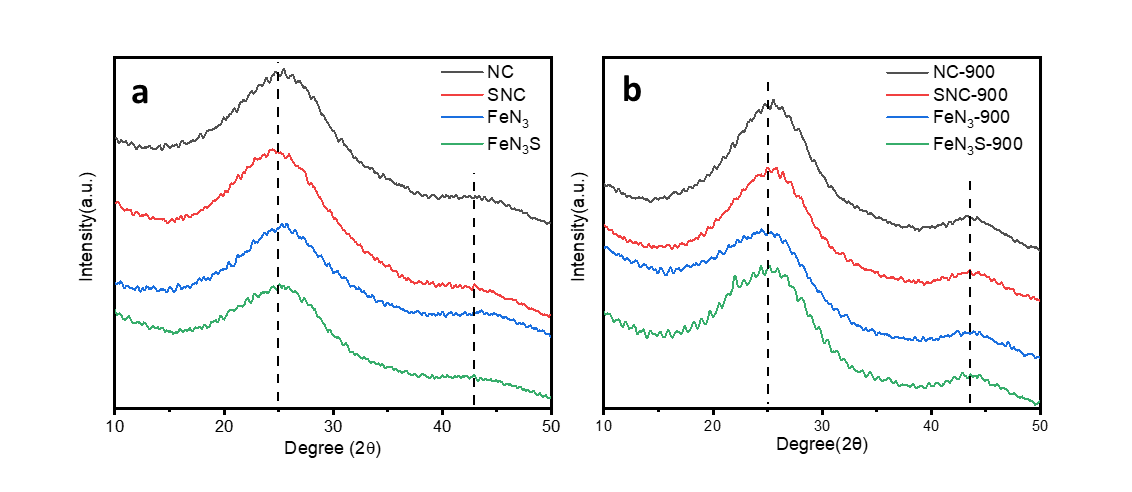


**Figure S8.** PXRD patterns of (a) NC, SNC, FeN_3_ and FeN_3_S; (b) NC-900, SNC-900, FeN_3_-900 and FeN_3_S-900.


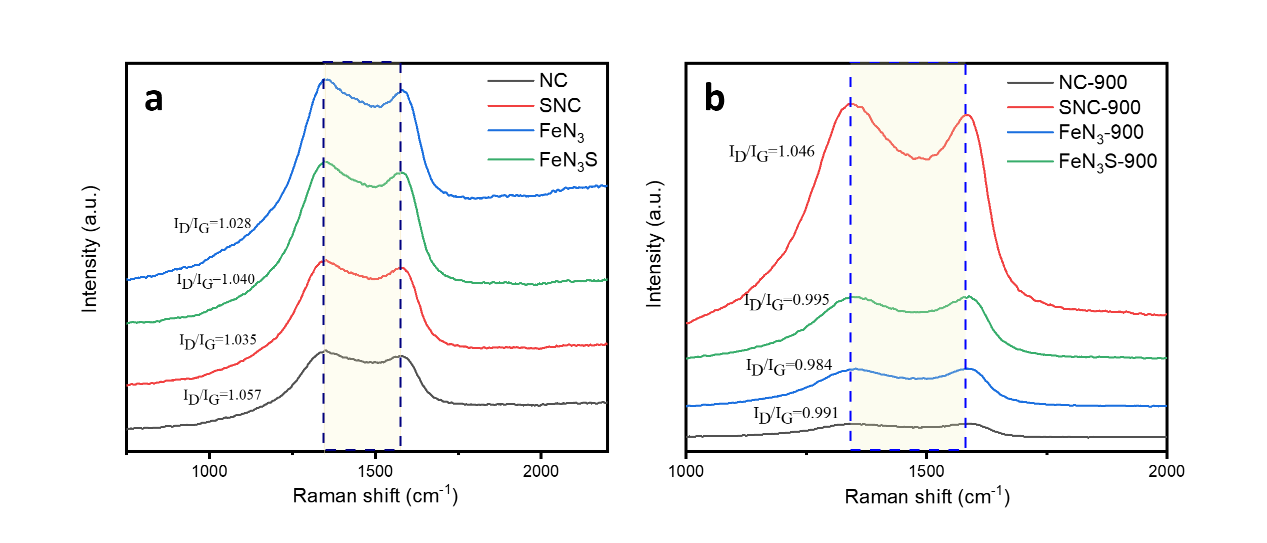


**Figure S9.** Raman spectra of (a) NC, SNC, FeN_3_ and FeN_3_S; (b) NC-900, SNC-900, FeN_3_-900 and FeN_3_S-900.


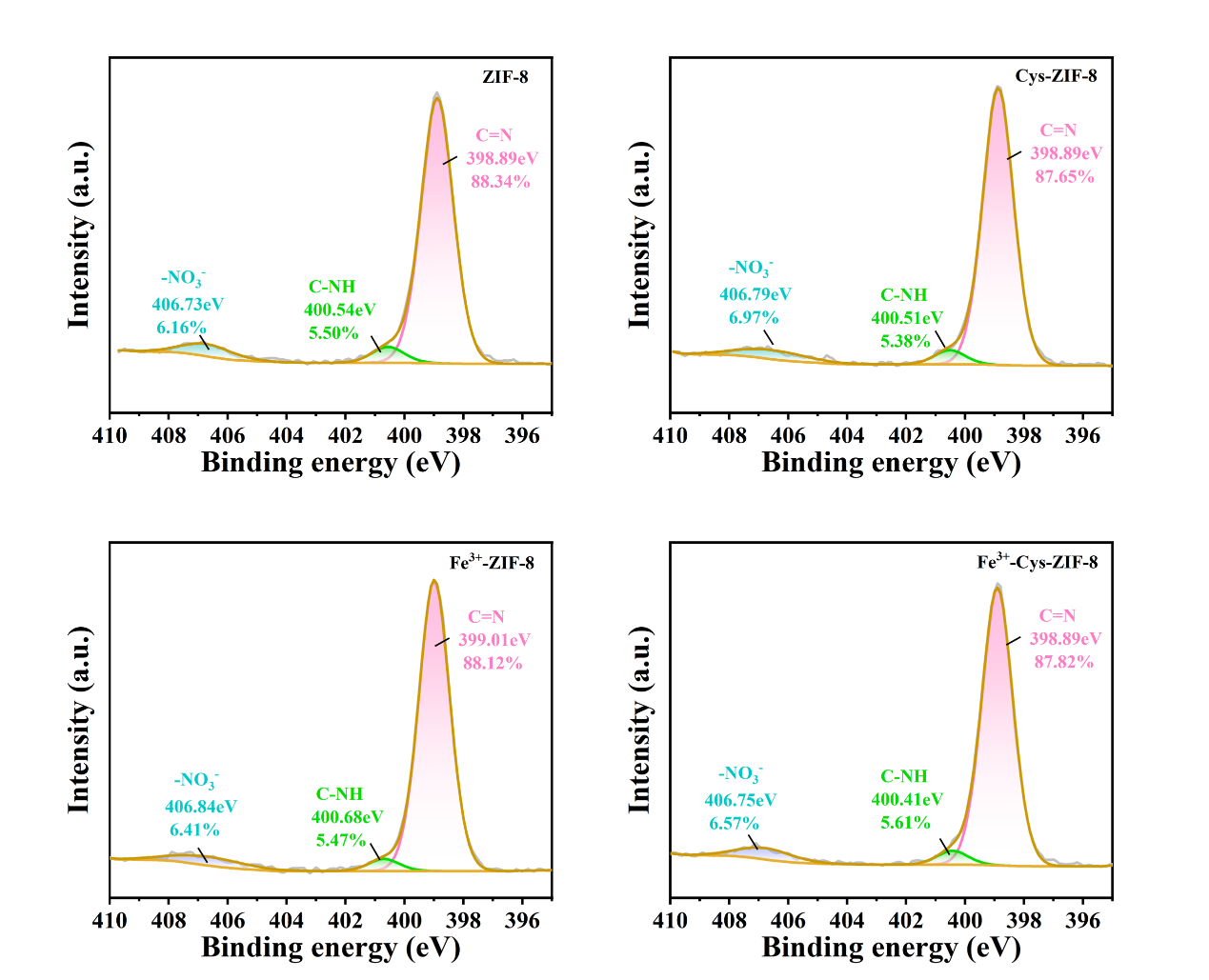


**Figure S10.** XPS spectra of N 1s of ZIF-8, Cys-ZIF-8, Fe^3+^-ZIF-8 and Fe^3+^-Cys-ZIF


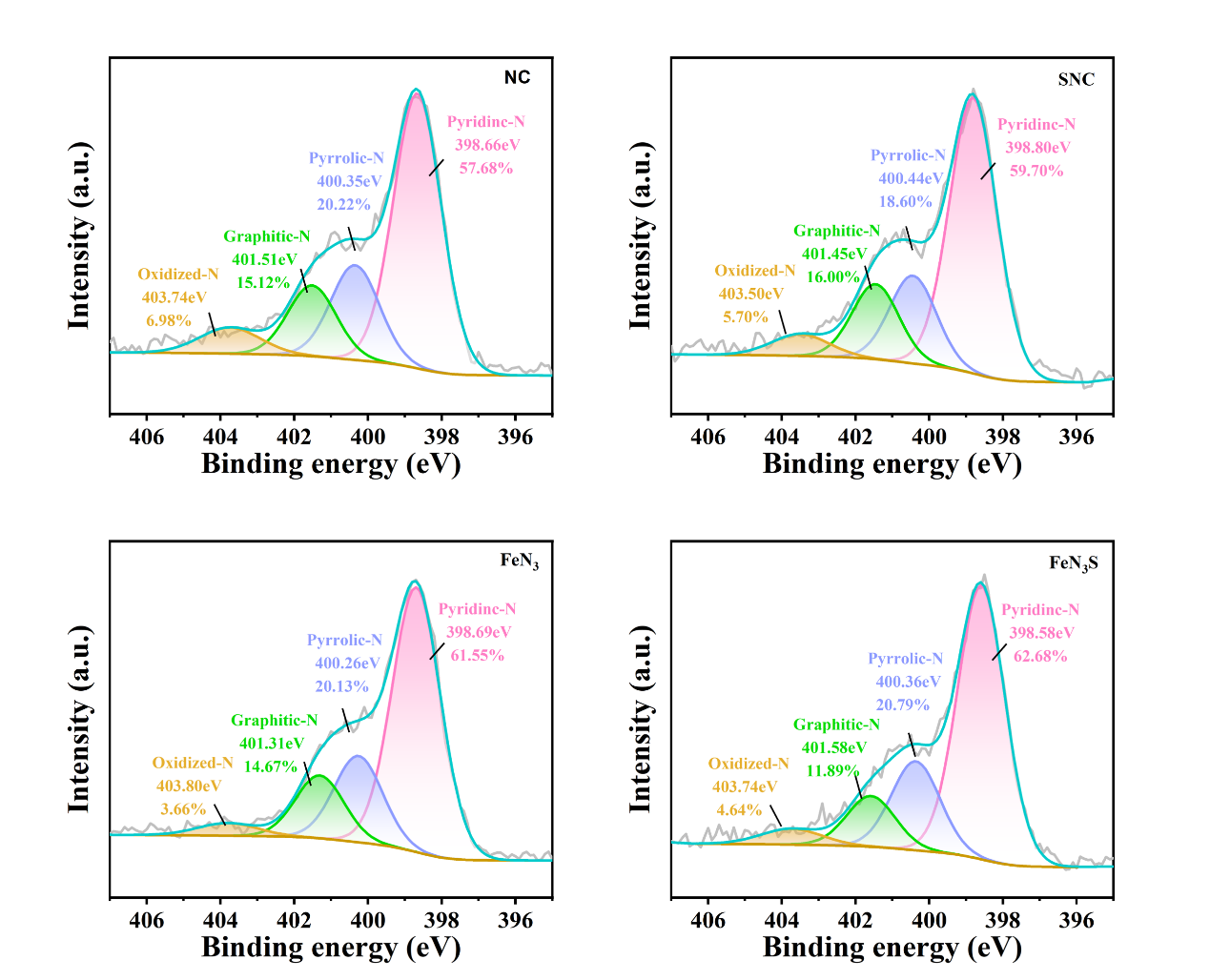


**Figure S11.** XPS spectra of N 1s of NC, SNC, FeN_3_ and FeN_3_S.


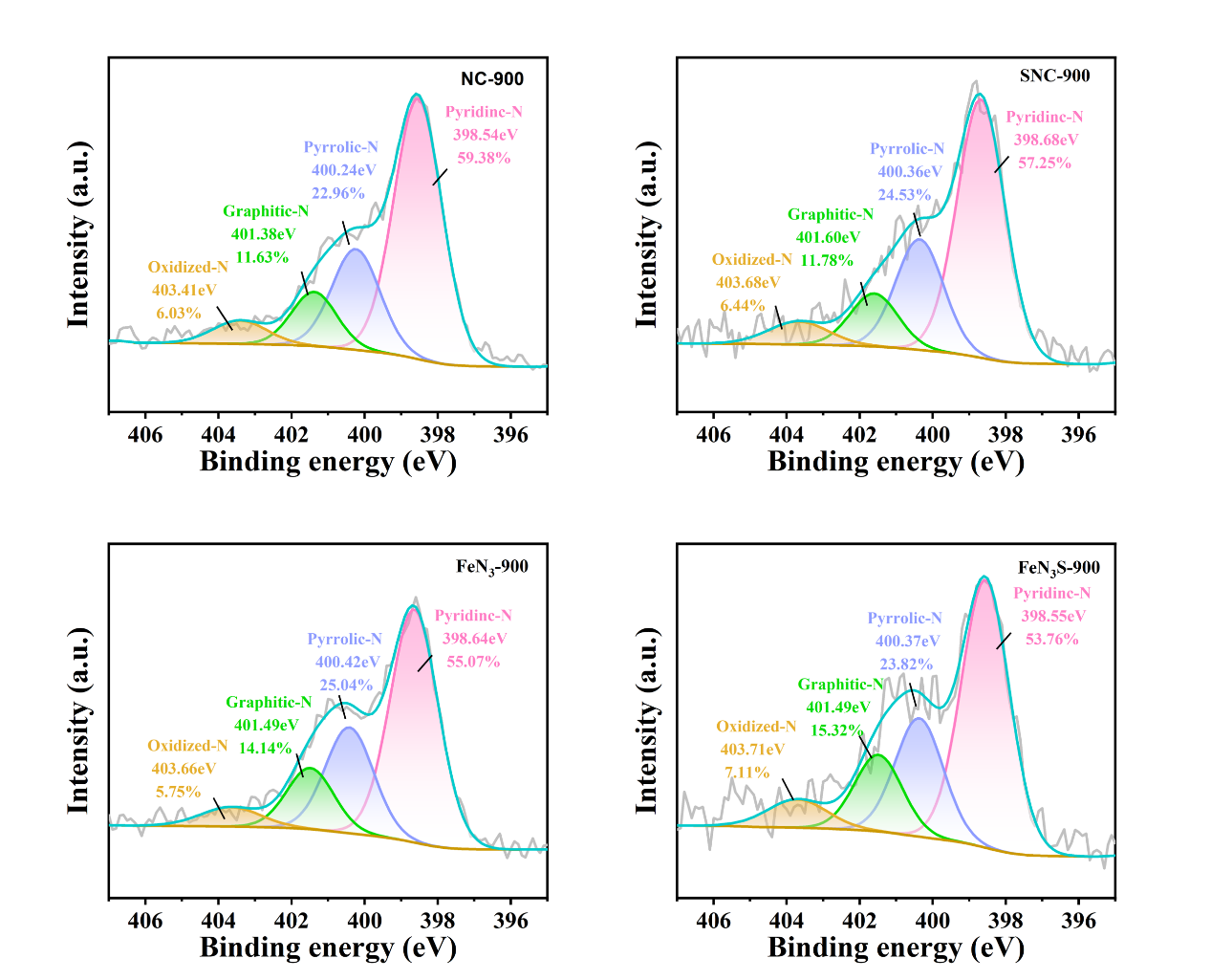


**Figure S12.** XPS spectra of N 1s of NC-900, SNC-900, FeN_3_-900 and FeN_3_S-900.


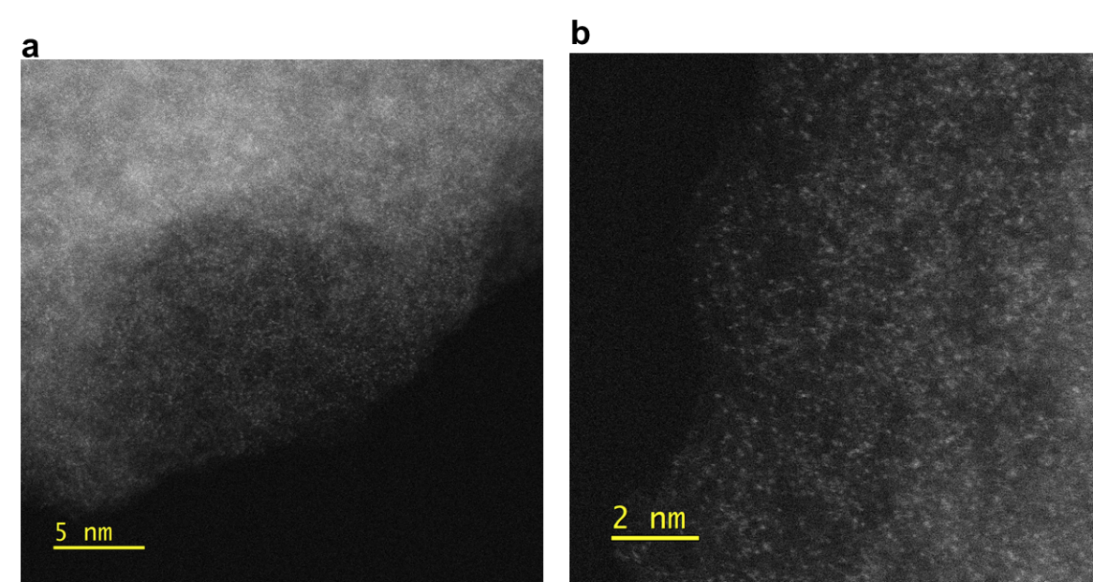


**Figure S13**. HAADF-STEM image of FeN_3_S showing the atomically dispersed Fe or Zn single-atom sites as bright dots.


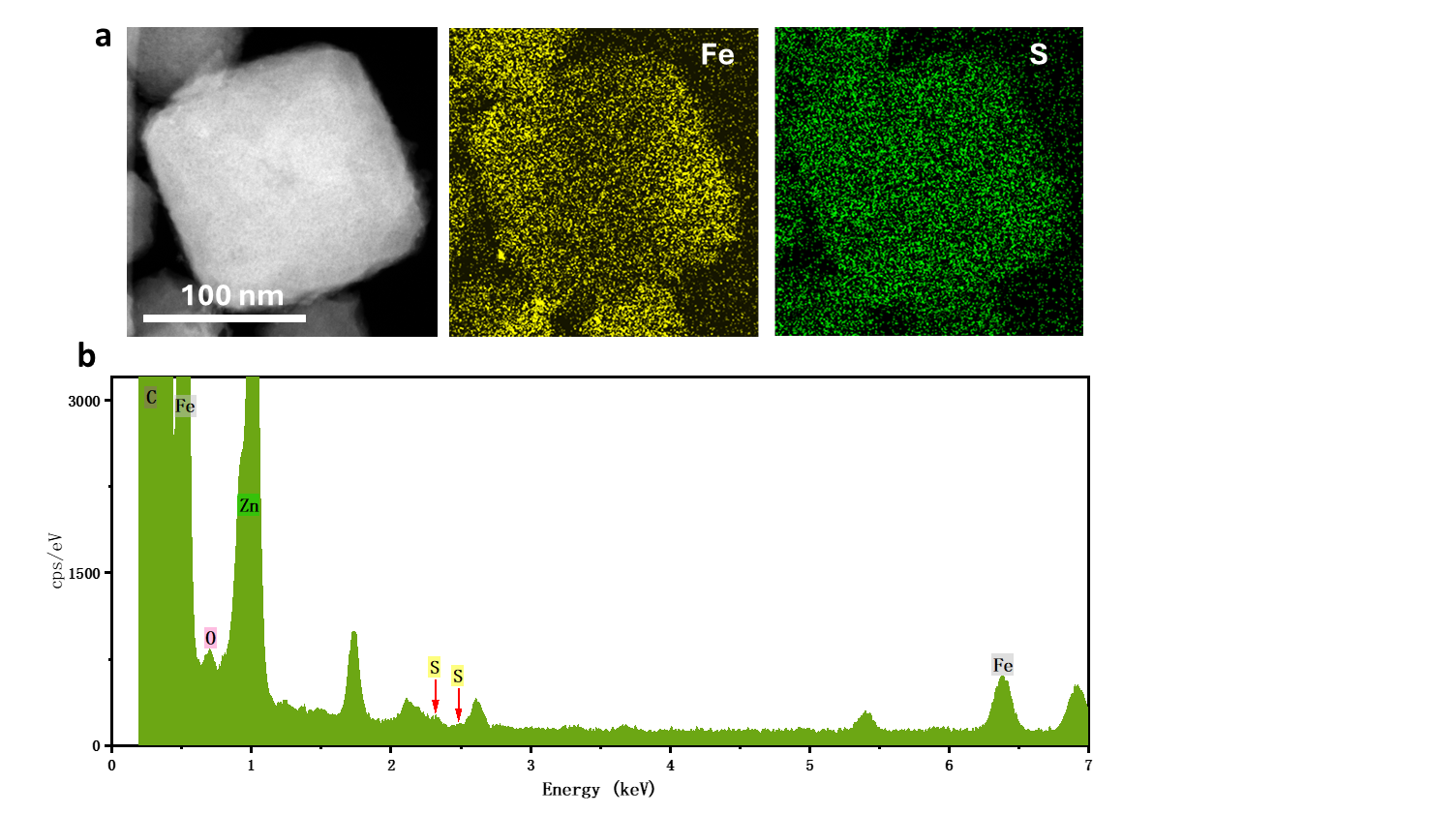


**Figure S14.** (a) HAADF-STEM image and corresponding EDS mapping of FeN_3_S, showing the uniform distribution of Fe and S. (b) Elemental analysis confirming the presence of Fe and S in FeN_3_S


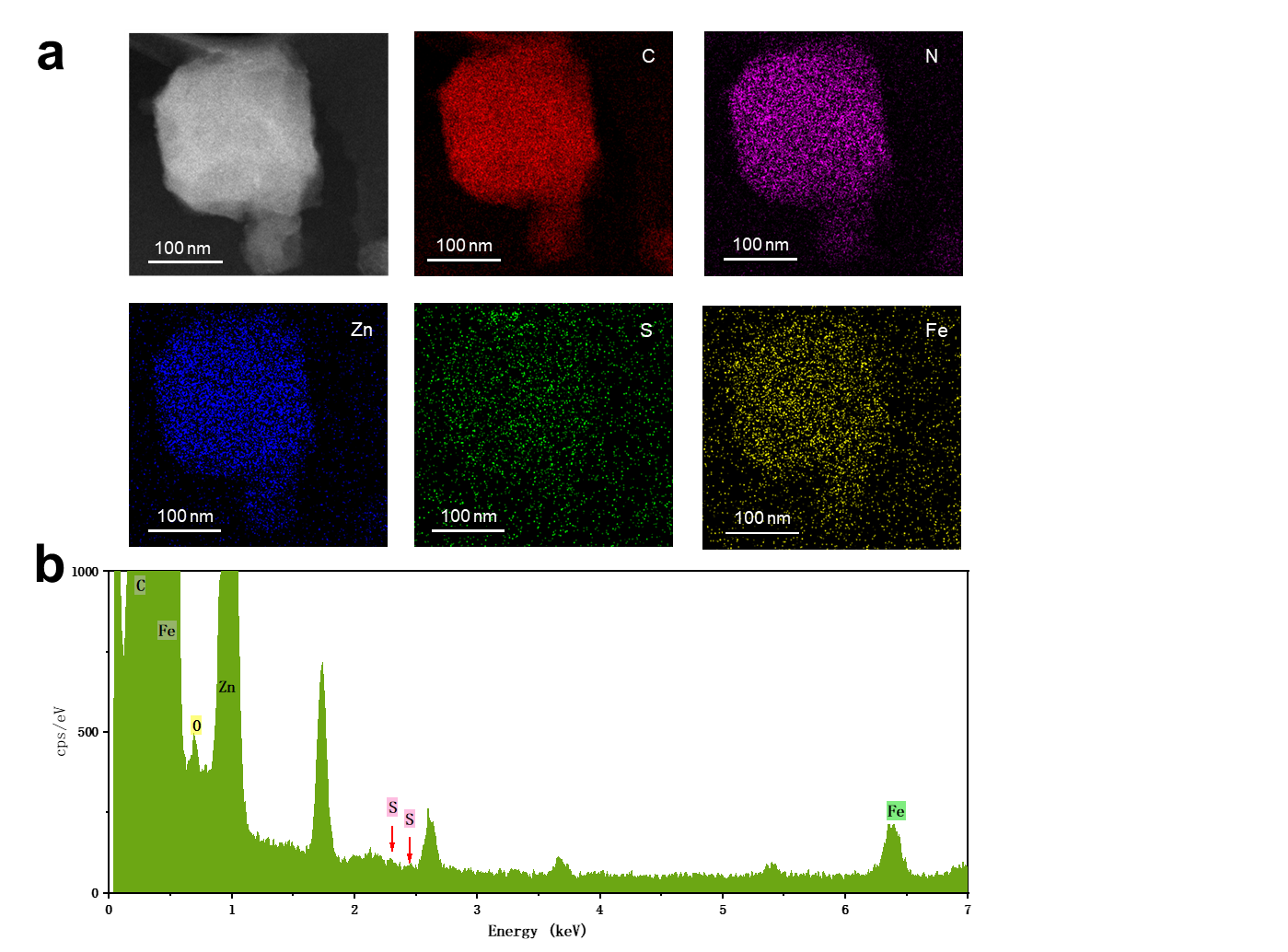


**Figure S15.** (a) HAADF-STEM image and corresponding EDS mapping of FeN_3_S-900, showing the uniform distribution of Fe, Zn, N, C and S. (b) Elemental analysis confirming the presence of Fe and S in FeN_3_S


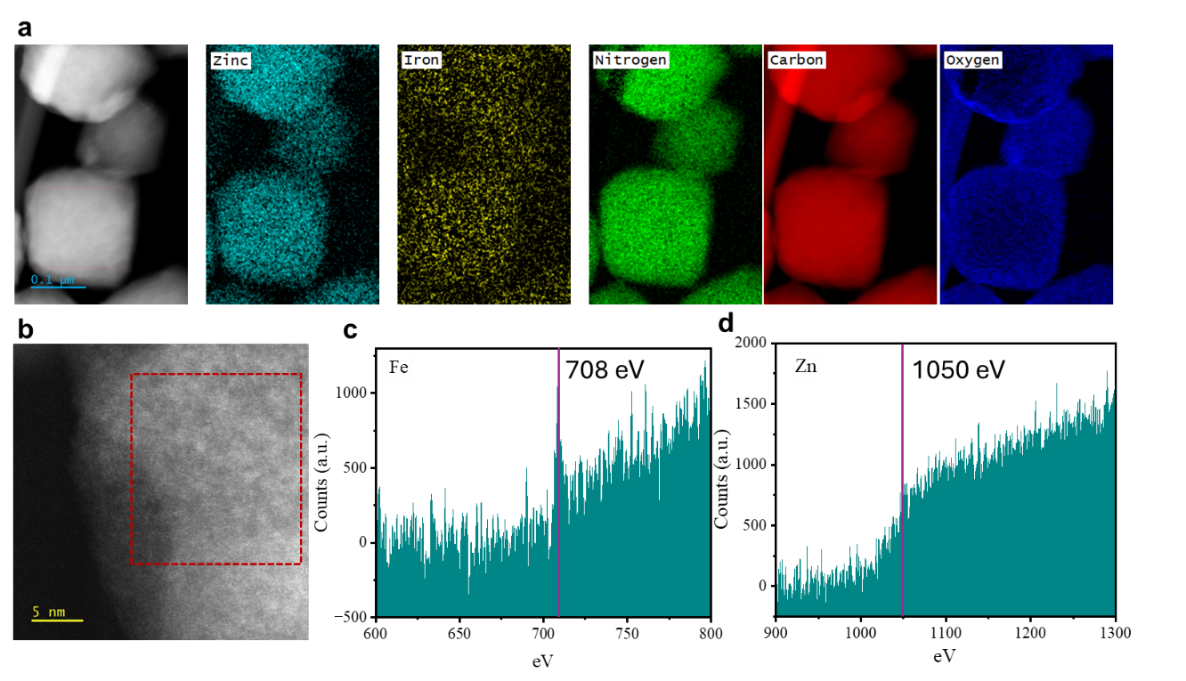


**Figure S16**. (a) HAADF-STEM image and EELS mapping; (b) High-resolution STEM image with marked region for EELS acquisition; EELS spectrum collected from the selected region showing the (c) Fe L-edge and (d) Zn L-edge of FeN_3_S. Sulphur was not observed in the EELS spectra due to the thick nature of the sample and high inelastic scattering from the carbon matrix which screens the EELS signal of the other elements – however for the same sample Sulphur was detected with EDS see Figure S15.


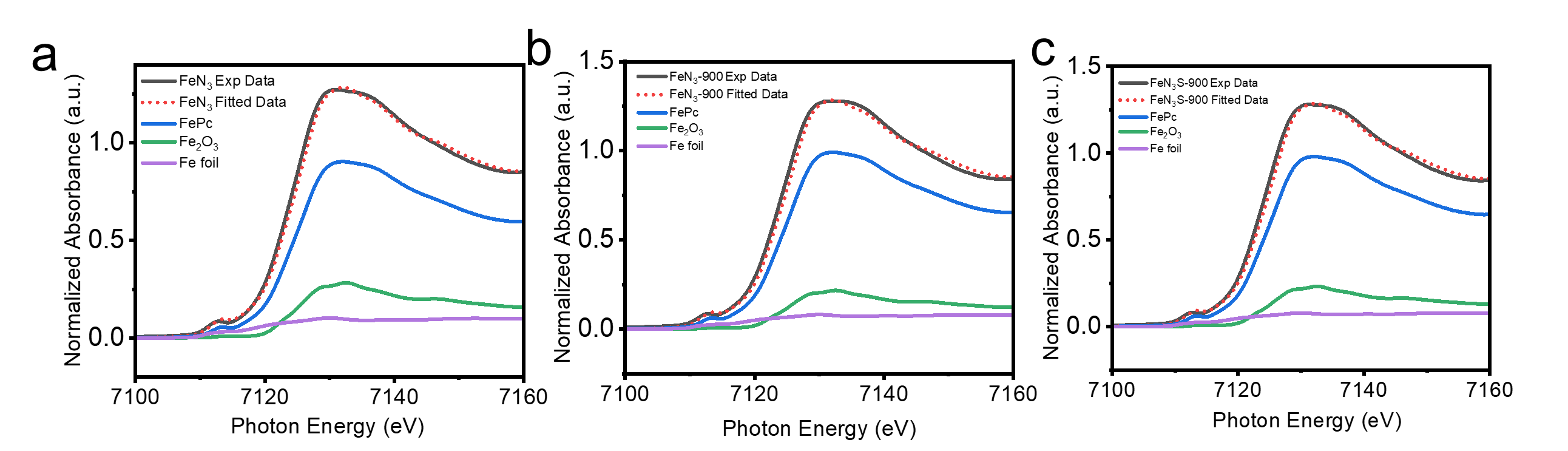


**Figure S17**. Linear combination fitting of the XANES data of FeN_3_, FeN_3_S-900, FeN_3_S-900 collected at the Fe K-edge.


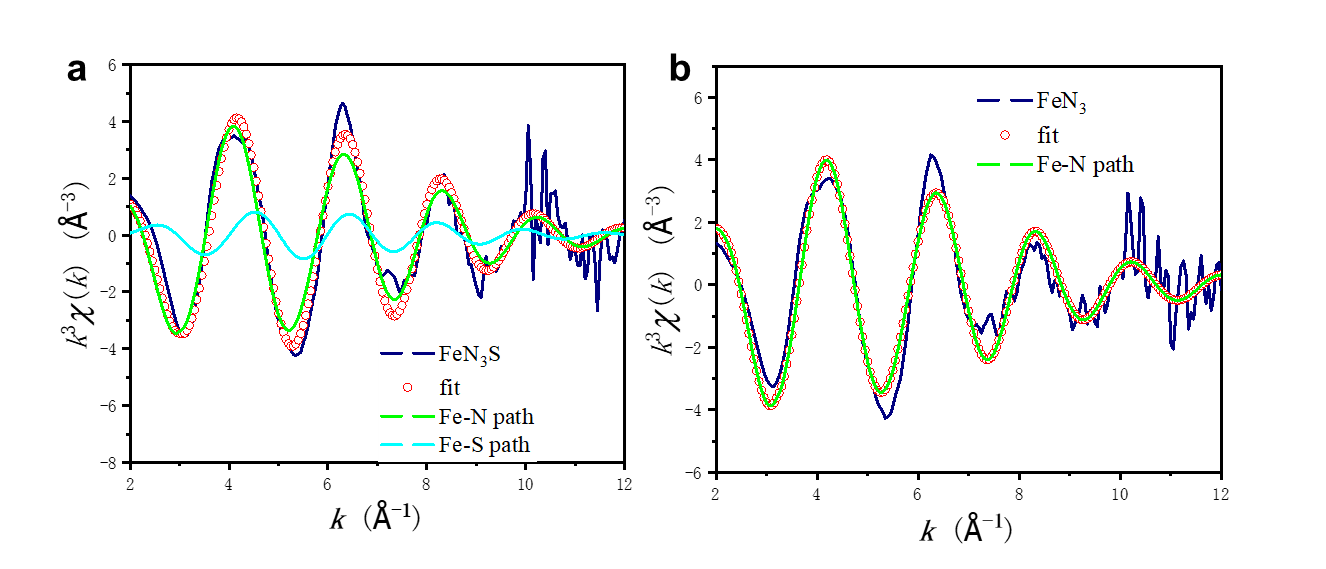


**Figure S18.** Fe K-edge EXAFS analysis of (a) FeN_3_S and (b) FeN_3_ in k spaces. The measured and calculated spectra are in good agreement. The best fit parameters are shown in Table S3.


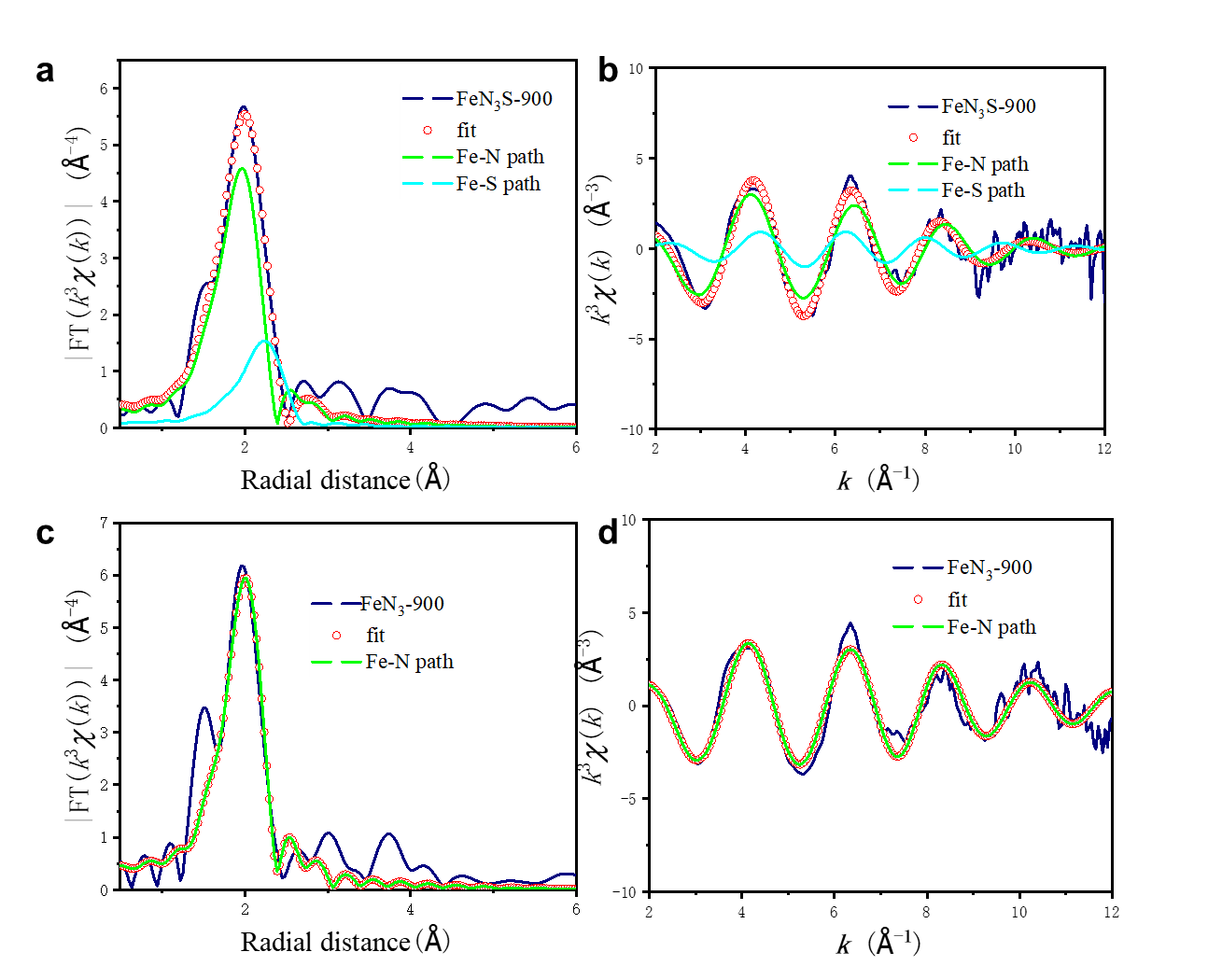


**Figure S19.** Phase-corrected Fe K-edge EXAFS analysis of (a, b) FeN_3_S-900 and (c, d) FeN_3_-900 in (a, c) R space and (b, d) k spaces. The measured and calculated spectra are in good agreement. The best fit parameters are shown in Table S3.


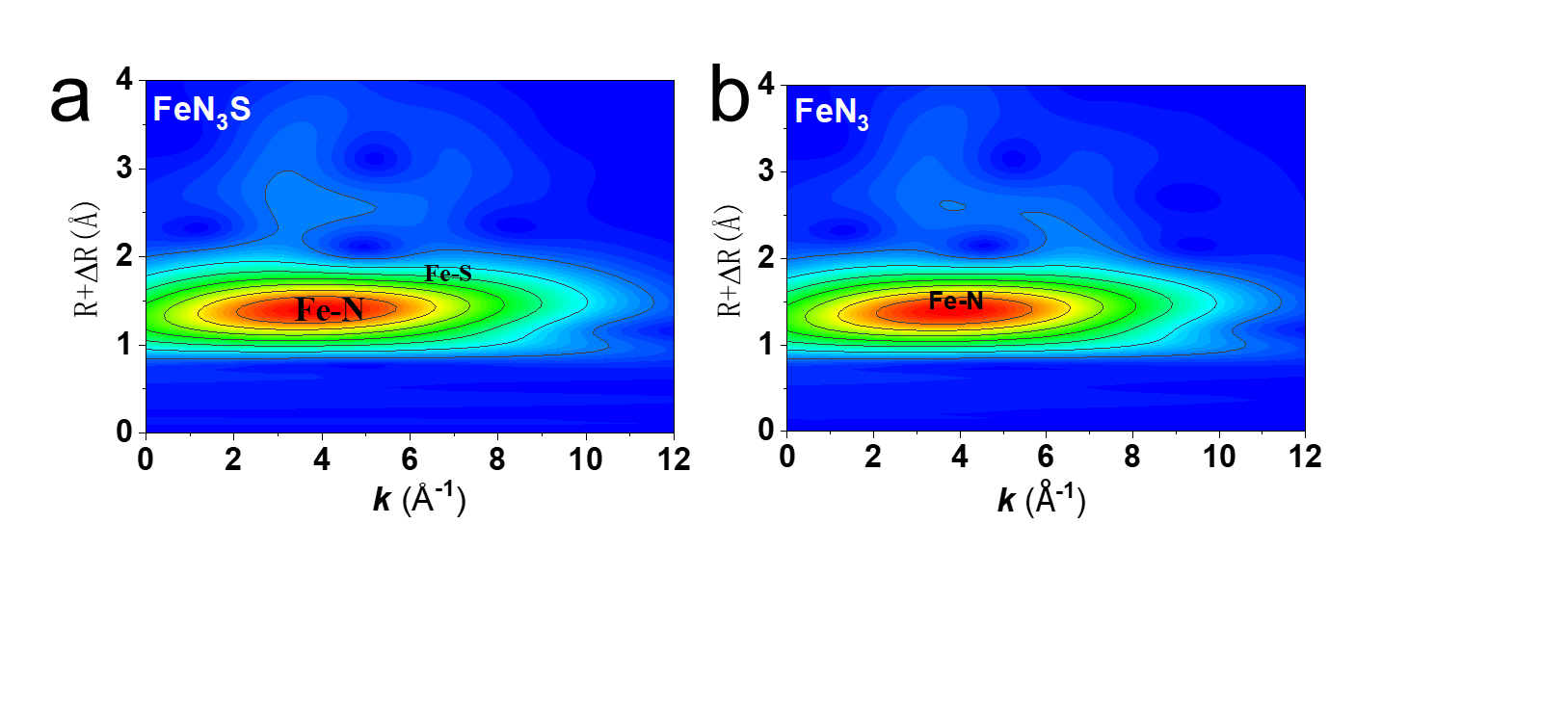


**Figure 20** Experimental 2D WT EXAFS plots for (a) FeN_3_S, and (b) FeN_3_.


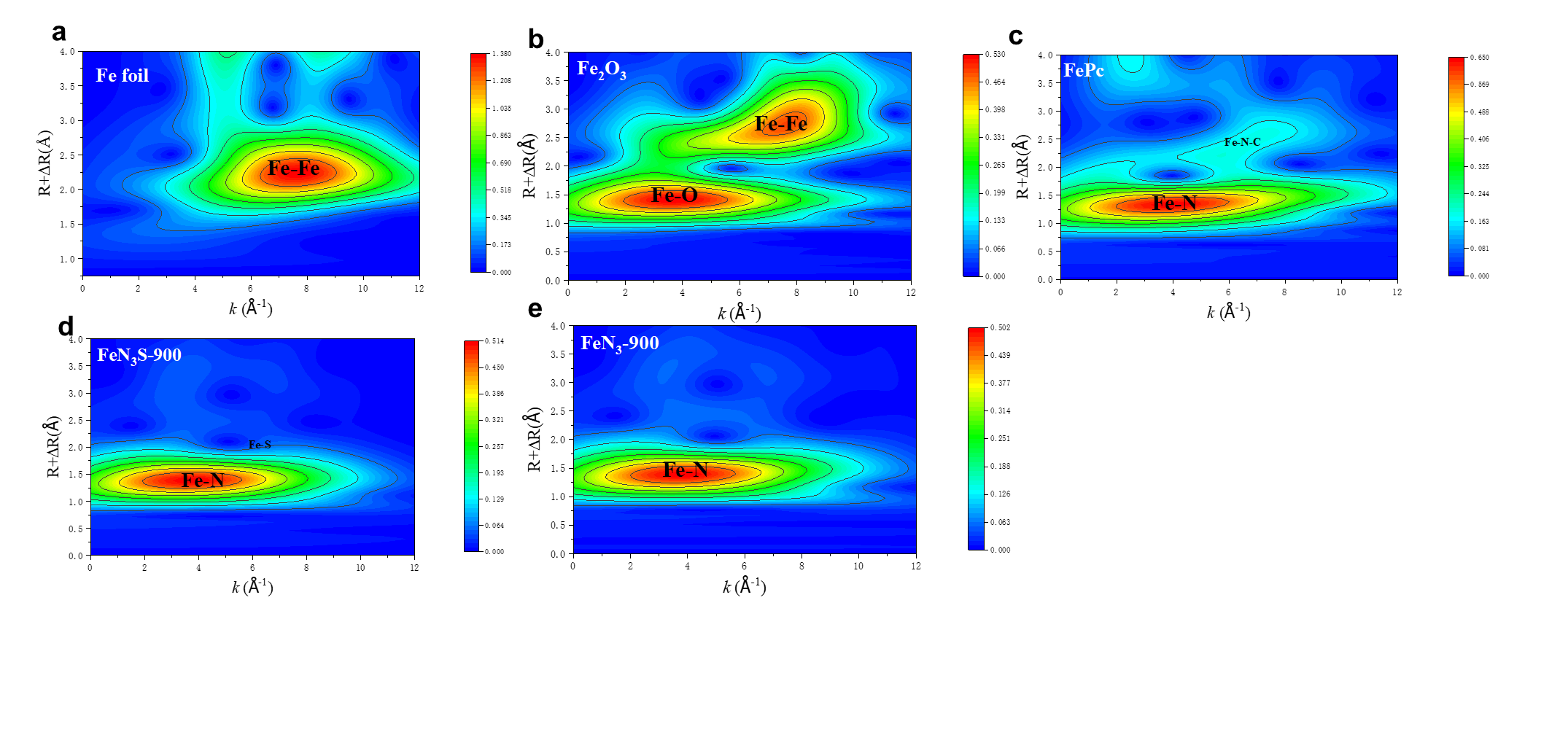


**Figure 21** Experimental 2D WT EXAFS plots for (a) Fe foil, (b) Fe_2_O_3_, (c) FePc, (d) FeN_3_S-900, and FeN_3_-900.


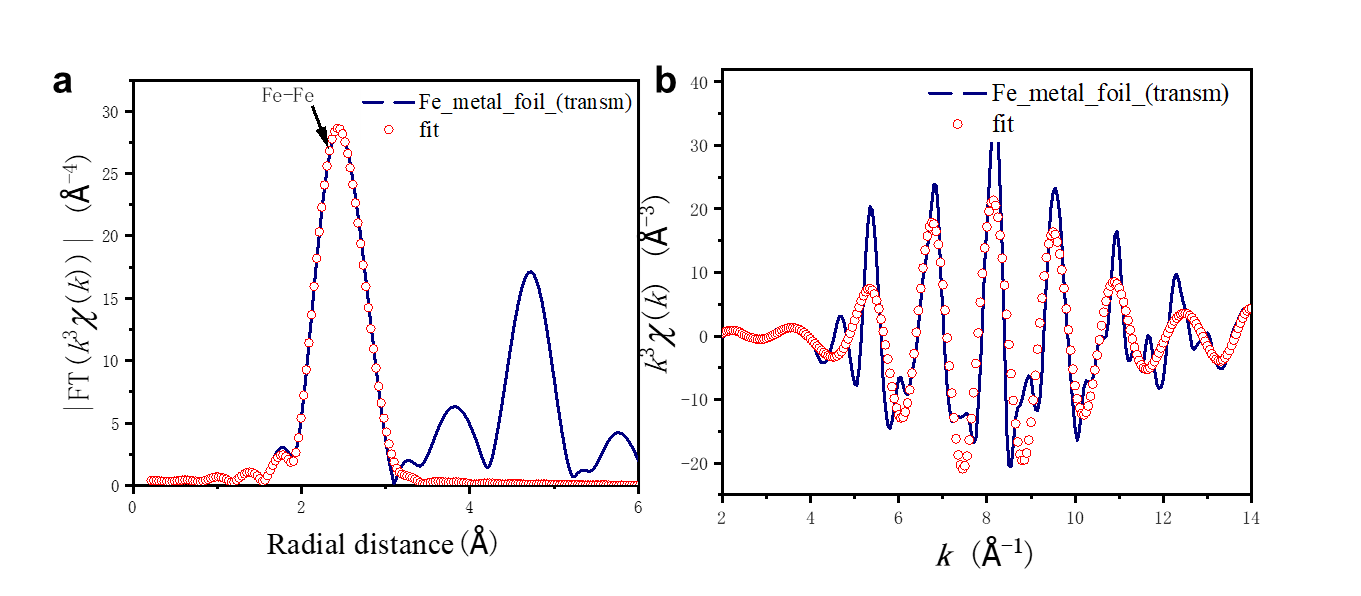


**Figure S22.** Phase-corrected Fe K-edge EXAFS analysis of Fe foil in (a) R space and (b) k spaces. The measured and calculated spectra are in good agreement. The best fit parameters are shown in Table S3.

**Figure S23**. UV–vis absorption spectra of NADH, NAD^+^ and FeN_3_S mixture after reacting for 1-5 min.


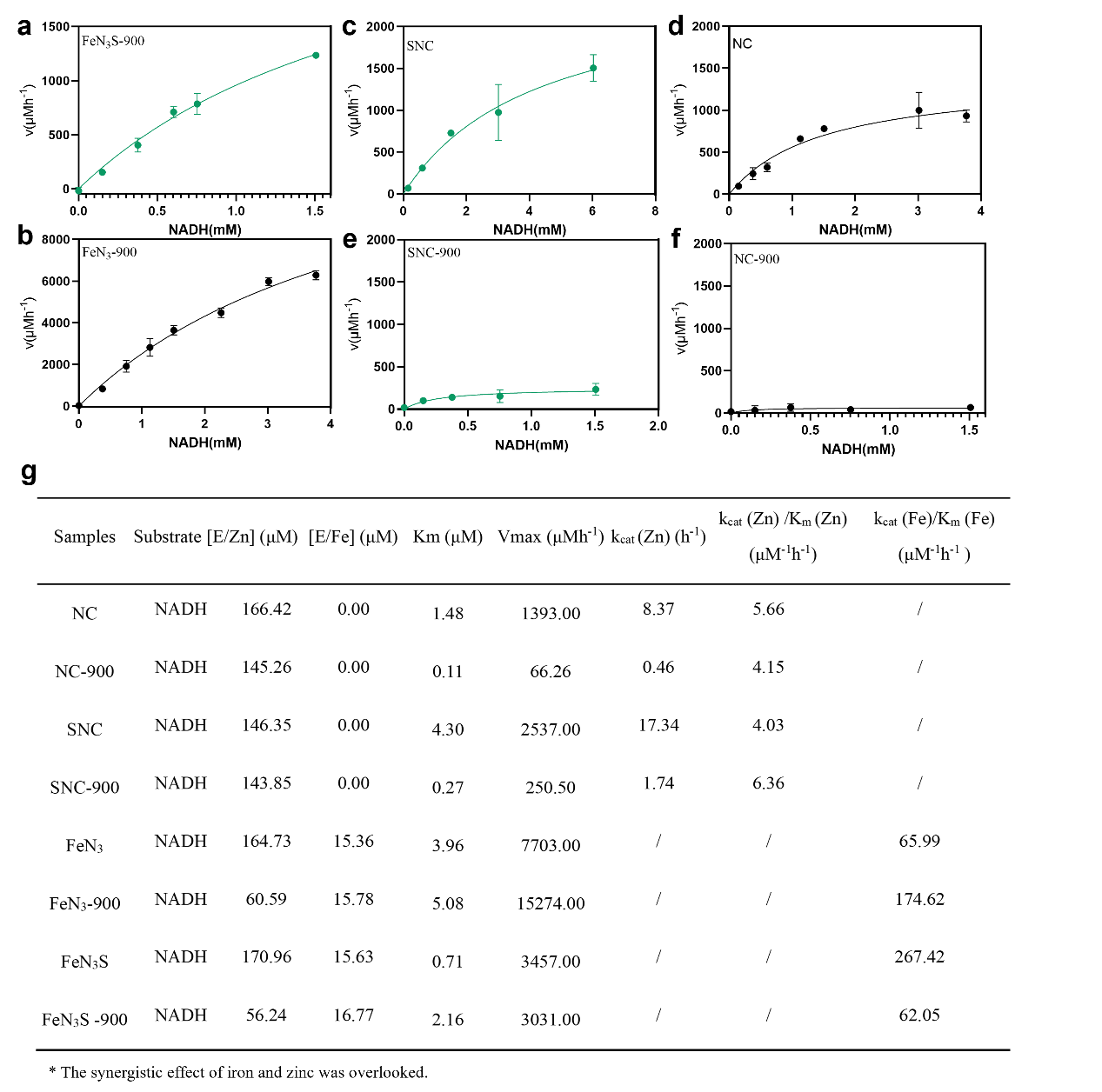


**Figure S24**. Michaelis–Menten kinetic analysis of NOX-like properties of (a) FeN_3_S-900; (b) FeN_3_-900; (c) SNC; (d) NC; (e) SNC-900; (f) NC-900. Quantitative data are shown as mean ± SD (n = 3). (g) The kinetics based on Zn or Fe active sites doped on prepared catalysts without considering the possible coupling effect between Zn and Fe. To eliminate the coupling effect of Zn for NADH, the catalytic efficiency of Zn was deducted during calculation for Fe-SACs according to ICP results. All experiments were conducted in NaAc/HAc buffer pH 4.5.


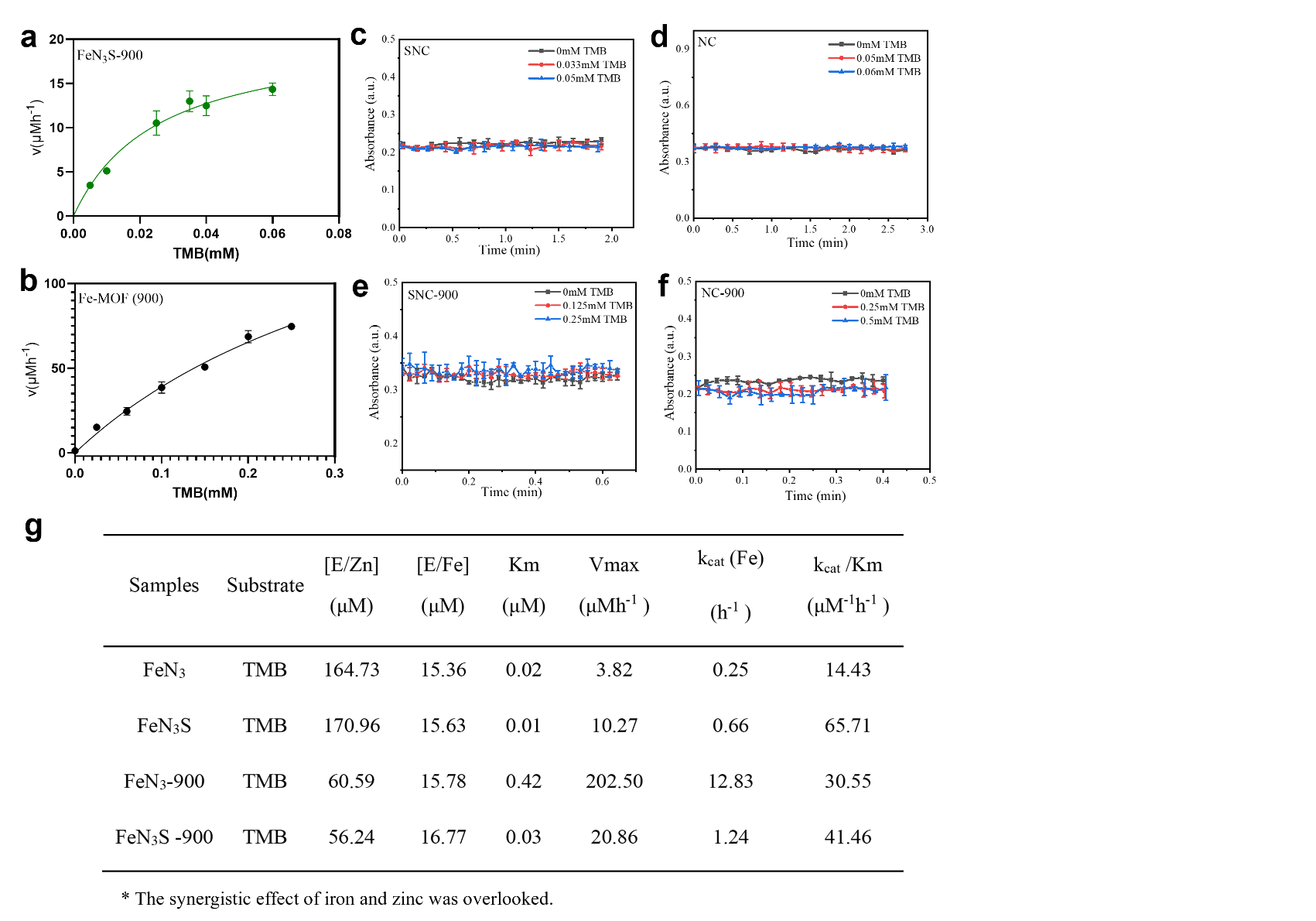


**Figure S25.** Michaelis–Menten kinetic analysis of OXD-like properties of (a) FeN_3_S-900 and (b) FeN_3_-900. OXD-like activity of (c) SNC; (d) NC; (e) SNC-900; (f) NC-900. Quantitative data are shown as mean ± SD (n = 3). (g) The kinetics based on Fe active sites doped on prepared catalysts. All experiments were conducted in NaAc/HAc buffer pH 4.5.

**
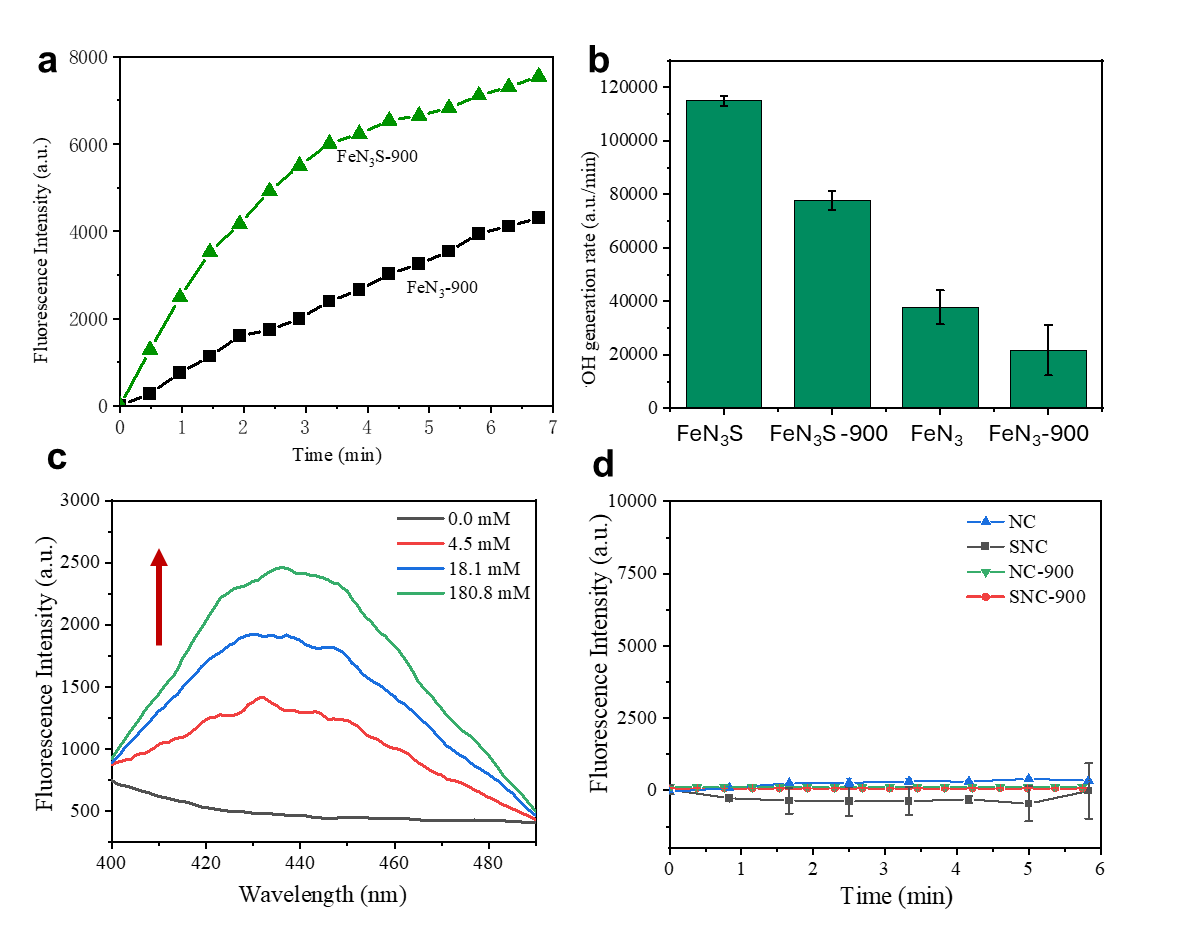
**

**Figure S26.** (a) Enzyme kinetics curve for catalyzing H_2_O_2_ into ·OH with each sample containing equal Fe single-atom loading. (b) Initial reaction rates of different samples. (c) Fluorescence intensity of FeN_3_S with varying H_2_O_2_ concentrations. (d) Control experiments with NC, SNC, SNC-900, and NC-900. All experiments were conducted in NaAc/HAc buffer pH 4.5. Quantitative data are shown as mean ± SD (n = 3).

**Figure S27**. ·OH generation detected by ESR spectra for FeN_3_S+NADH, FeN_3_S+ H_2_O_2_ and FeN_3_+ H_2_O_2_. ESR experiments were carried out to detect the hydroxyl radicals by using 5,5- dimethyl-1-pyrroline N-oxide (DMPO) as a spin trapping agent.

**Figure S28.** ·OH generation detected by ESR spectra for FeN_3_-900 + H_2_O_2_ and FeN_3_S-900 + H_2_O_2_. ESR experiments were carried out to detect the hydroxyl radicals by using DMPO as a spin trapping agent.


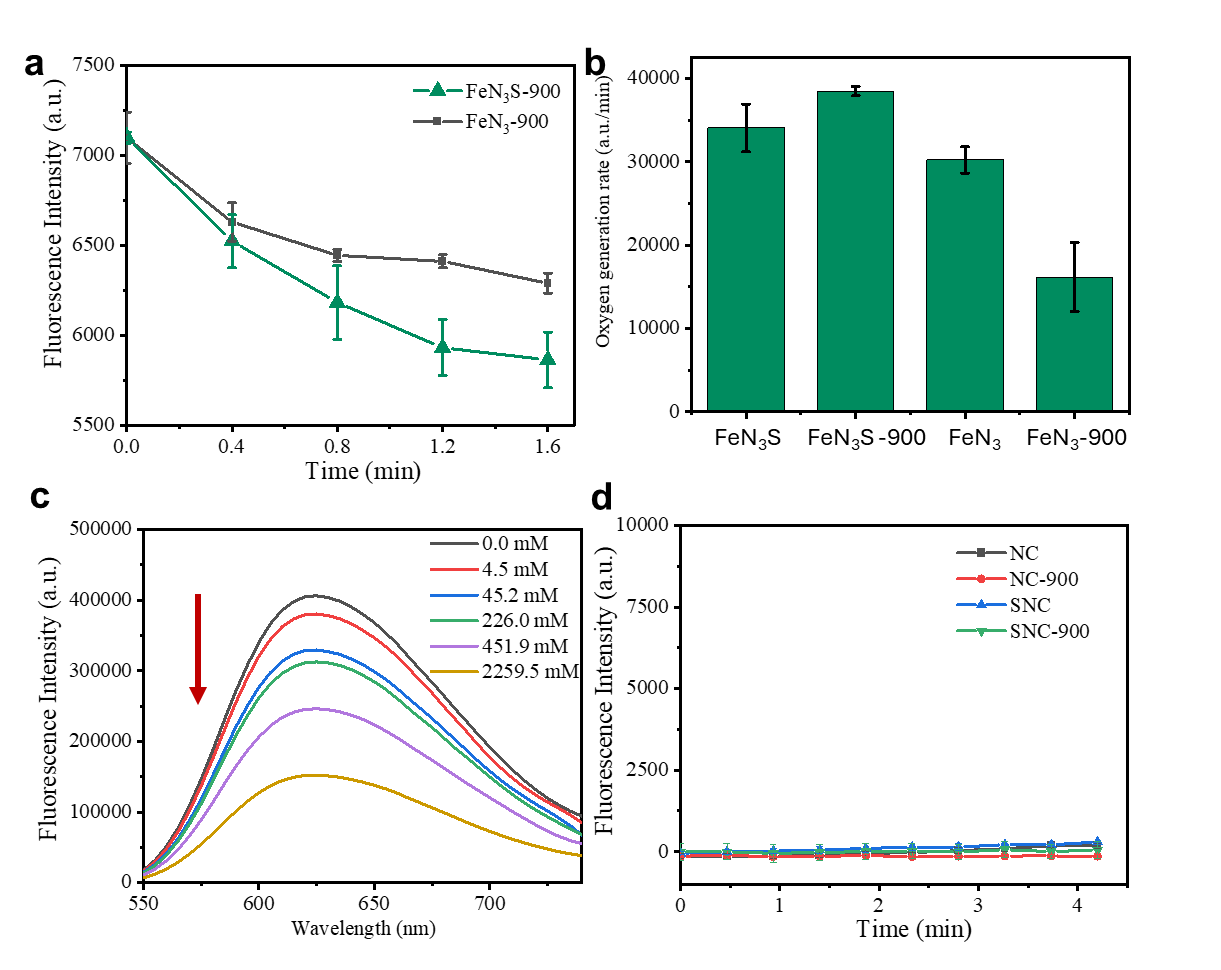


**Figure S29.** (a) Enzyme kinetics curve for catalyzing H_2_O_2_ into O_2_ with each sample containing equal Fe single-atom loading. (b) Initial reaction rates of different samples. (c) Fluorescence intensity of FeN_3_S with varying H_2_O_2_ concentrations. (d) Control experiments with NC, SNC, SNC-900, and NC-900. Quantitative data are shown as mean ± SD (n = 3).


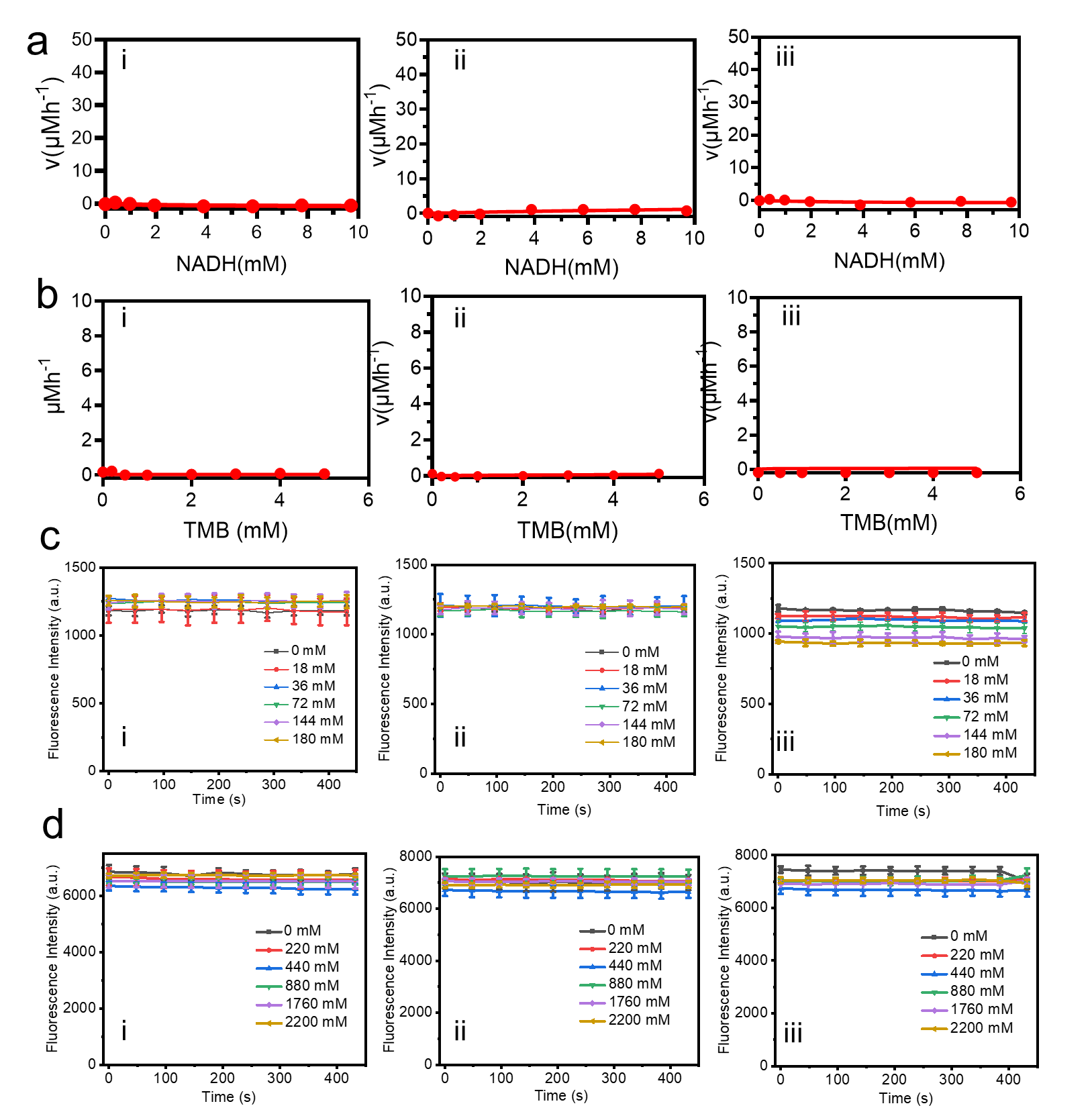


**Figure S30**. (a) NOX-like, (b) OXD-like, (c) POD-like, (d) CAT-like activity of Fe SAC using the different S-ligands: (i) 1,2-echanedithiol, (ii) 1-butanethiol, and (iii) thioglycolic acid. Quantitative data are shown as mean ± SD (n = 3).


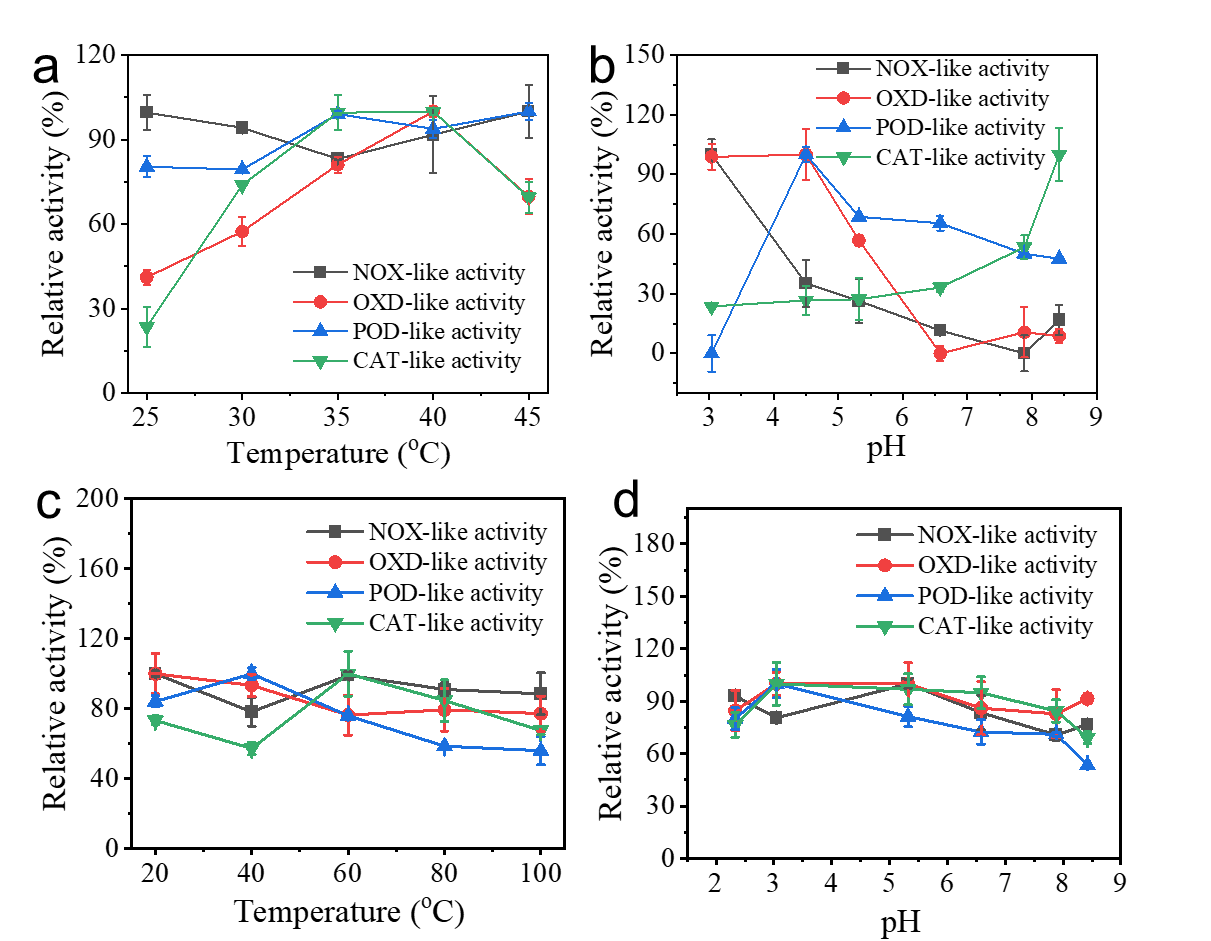


**Figure S31**. (a) Temperature-dependent and (b) pH-dependent catalytic activity of FeN_3_S. Catalytic stability of FeN_3_S after incubated with buffers at (c) different temperatures for 1 hour or (d) pH for 1.5 h. Quantitative data are shown as mean ± SD (n = 3).

**
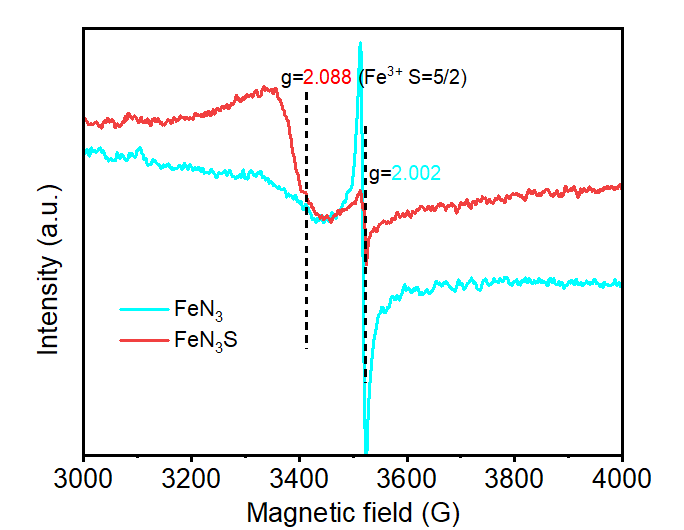
**

**Figure S32**. EPR spectra of the FeN_3_ and FeN_3_S.

**
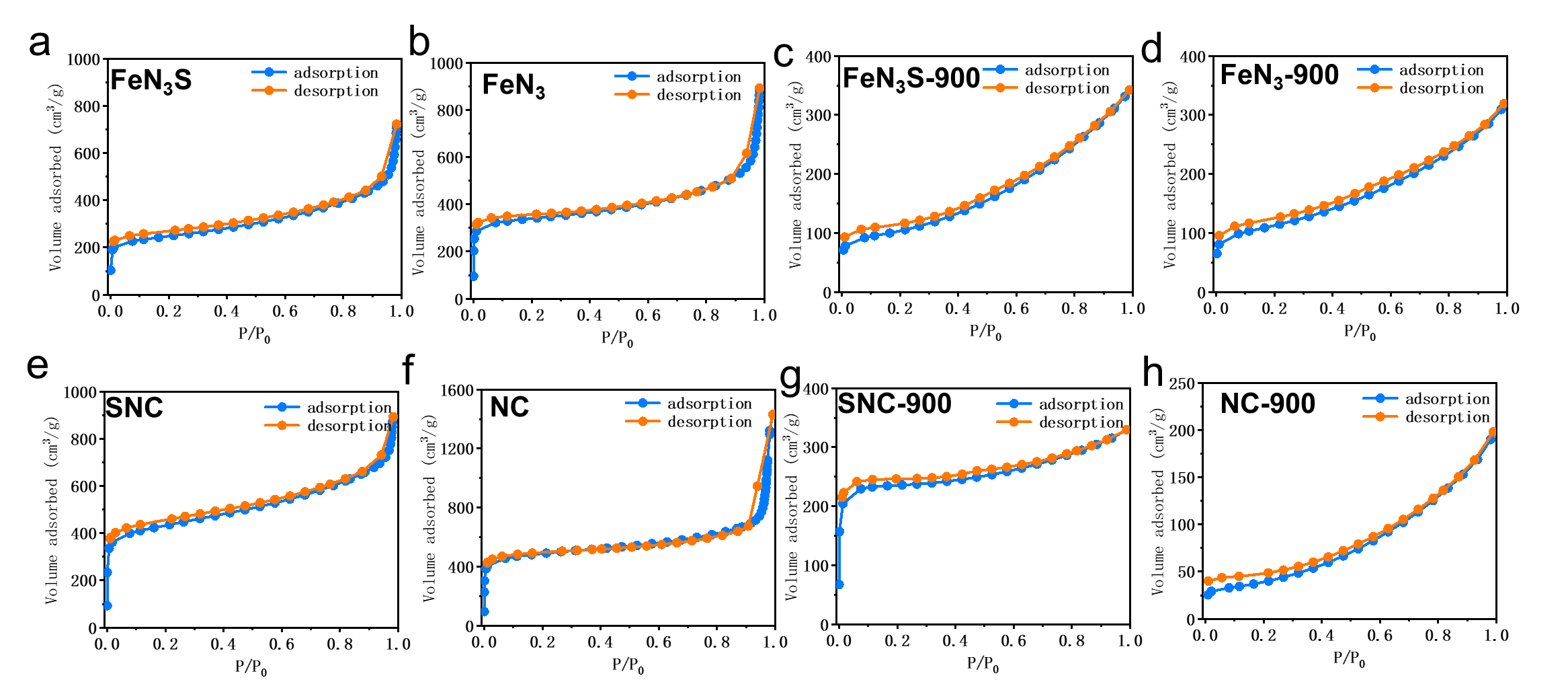
Figure S33**. N_2_ adsorption-desorption isotherms of FeN_3_S, FeN_3_, FeN_3_S-900, FeN_3_-900, SNC, NC, SNC-900, and NC-900.


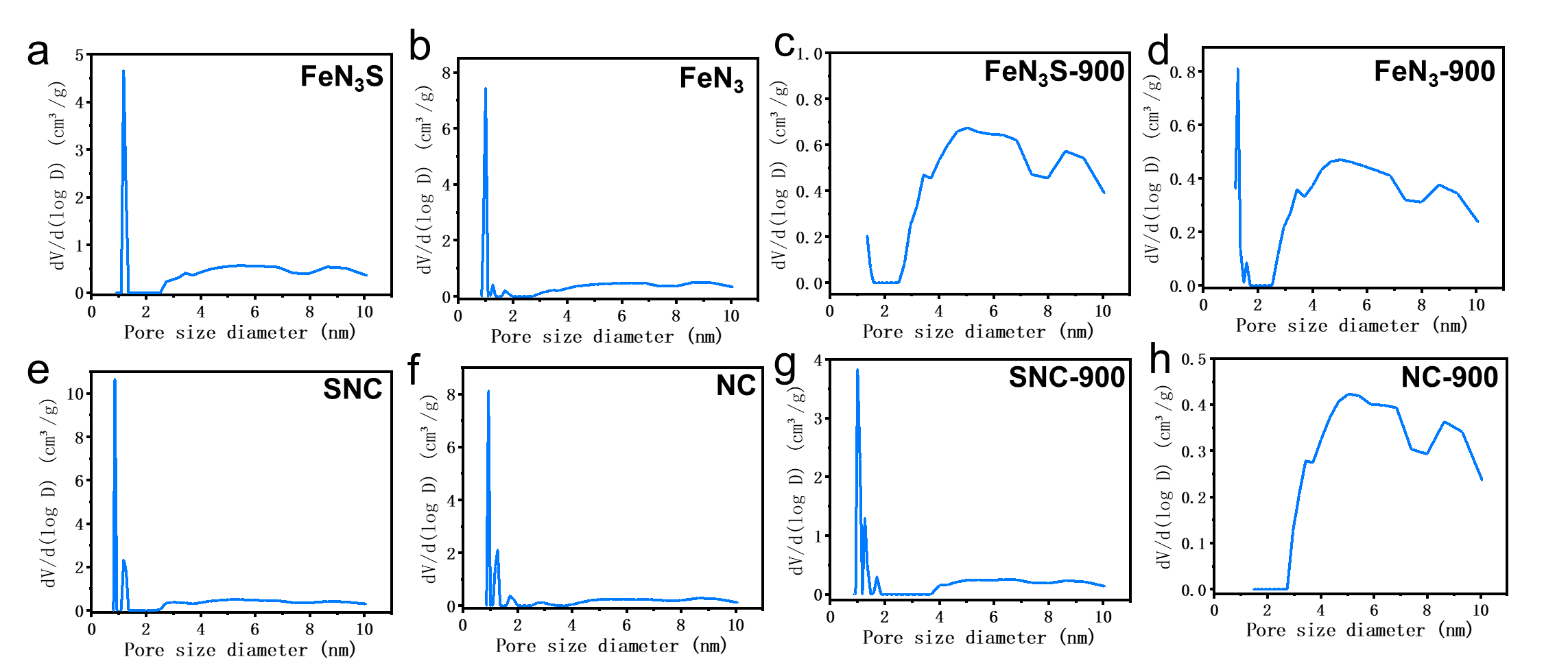


**Figure S34**. Pore size distribution calculated using DFT of FeN_3_S, FeN_3_, FeN_3_S-900, FeN_3_-900, SNC, NC, SNC-900, and NC-900.


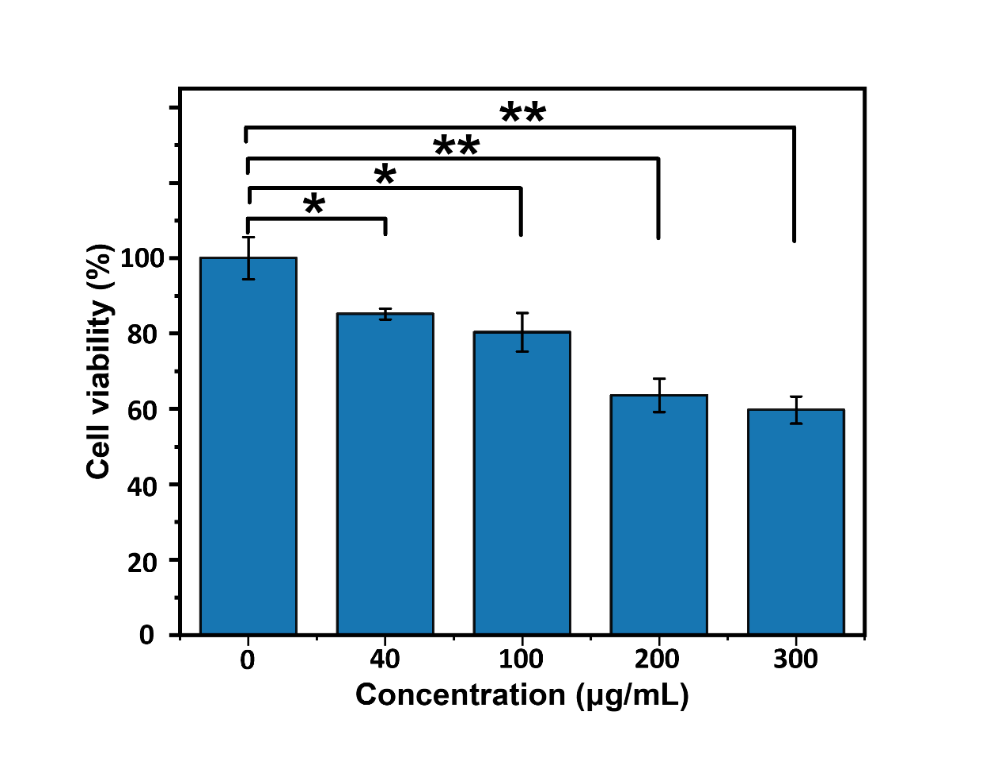


**Figure S35**. Viability of 3T3 fibroblasts co-cultured with FeN_3_S at different concentrations for 24 h. Quantitative data are shown as mean ± SD (n = 3). One-way ANOVA and Sidak's multiple comparisons test were performed. *, p < 0.05; **, p < 0.01; ***, p < 0.001; ****, p < 0.0001; ns, no significance

**Figure S36**. The cytotoxicity of the released Fe, S, and Zn species from FeN_3_S toward healthy 3T3 fibroblasts after 24 h of incubation. Quantitative data are shown as mean ± SD (n = 3). One-way ANOVA and Sidak’s multiple comparisons test were performed. *, p < 0.05; **, p < 0.01; ***, p < 0.001; ****, p < 0.0001; ns, no significance


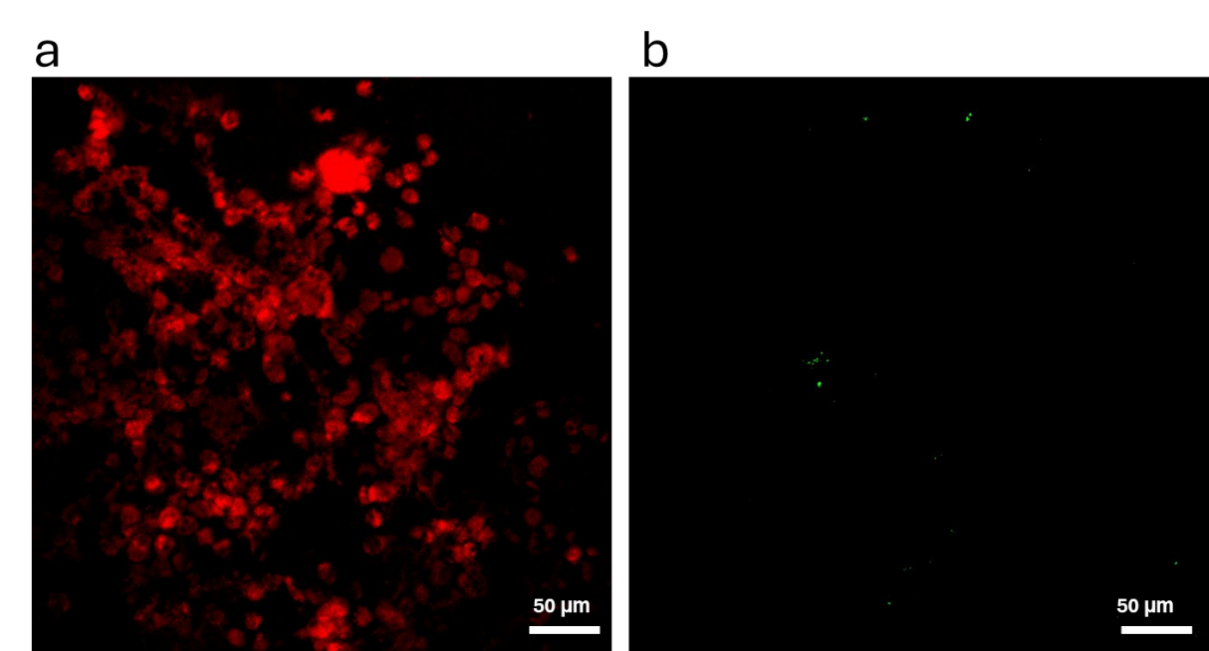


**Figure S37.** Fluorescence microscopy images showing mitochondrial depolarization in 4T1 cells cultured without any treatment for 24 h. Representative fluorescence images from n = 3 independent experiments (each experiment: 3 wells per condition, ≥5 random fields per well). Acquisition settings were identical across groups. No statistical analysis was performed for these images.


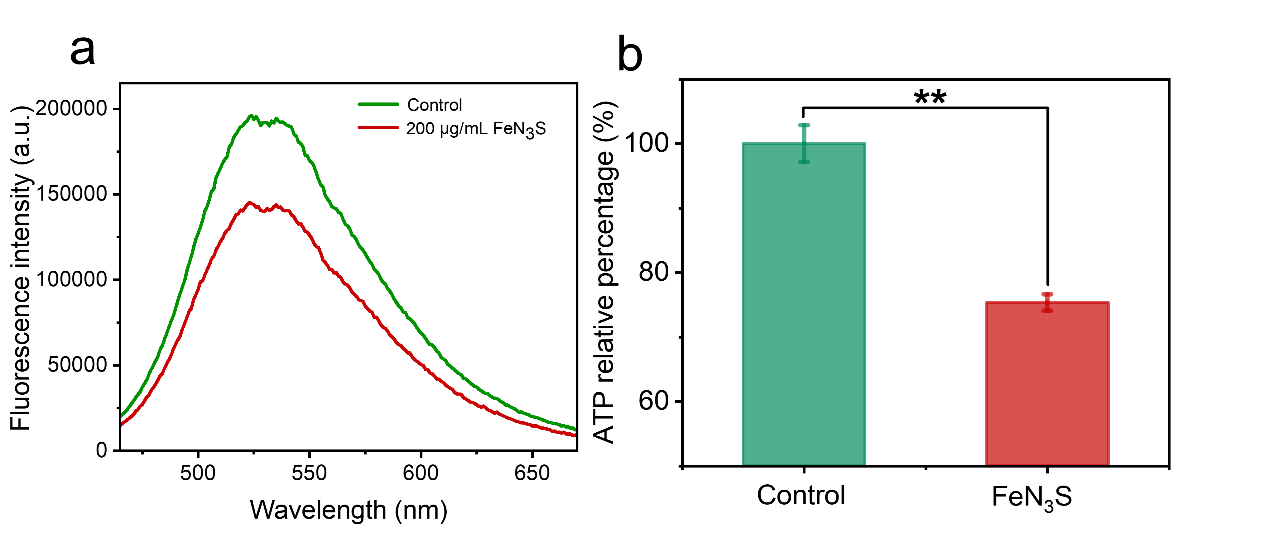


**Figure S38**. ATP levels in 4T1 cells after FeN_3_S treatment: (a) fluorescence intensity comparison; (b) relative ATP percentage. Quantitative data are shown as mean ± SD (n = 3). Two-tailed unpaired t-test with Welch’s correction was performed to compare the two groups. *, p < 0.05; **, p < 0.01; ***, p < 0.001; ****, p < 0.0001; ns, no significance


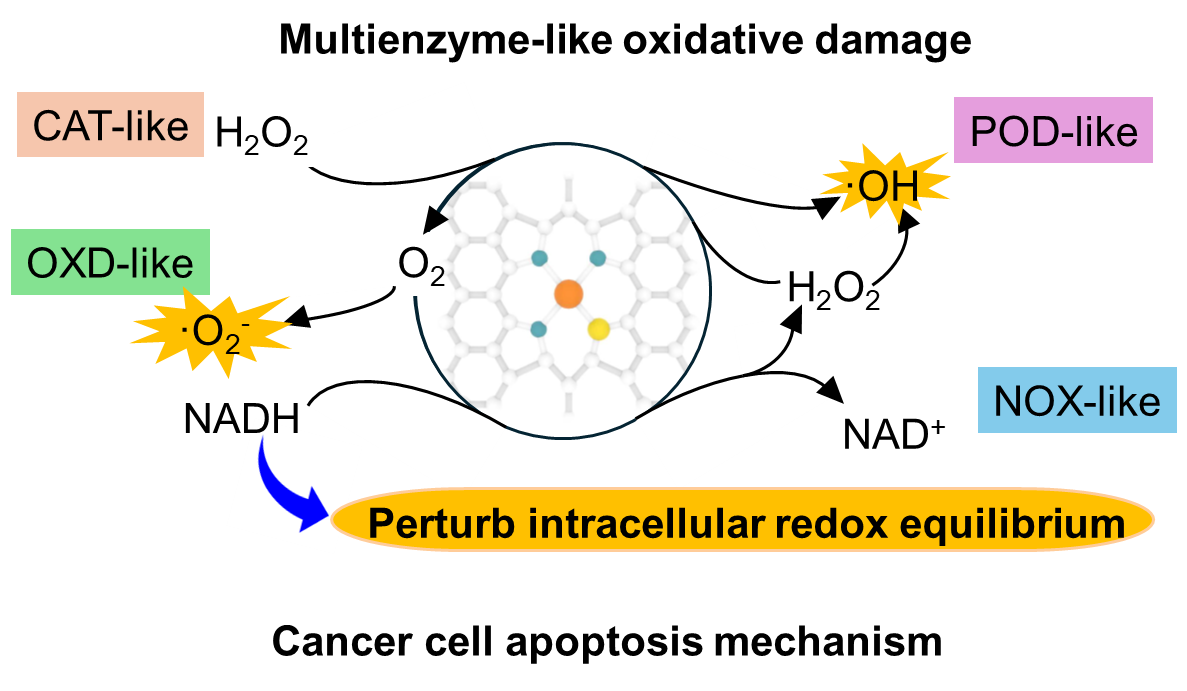


**Figure S39**. Multi-enzyme cascade–induced cell apoptosis mechanism.

**References:**

[1] L. Jiao, Y. Kang, Y. Chen, N. Wu, Y. Wu, W. Xu, X. Wei, H. Wang, W. Gu, L. Zheng, W. Song, C. Zhu. Unsymmetrically coordinated single Fe-N3S1 sites mimic the function of peroxidase. *Nano Today* **2021**, 40, 101261.

[2] W. Liu, Q. Chen, J. Wu, F. Zhang, L. Han, J. Liu, H. Zhang, Z. Hao, E. Shi, Y. Sun, R. Zhang, Y. Wang, L. Zhang. Asymmetric Coordination of Iron Single‐Atom Nanozymes with Efficient Self‐Cascade Catalysis for Ferroptosis Therapy. *Adv. Funct. Mater.* **2024**, 34, 2312308.

[3] S. Ji, B. Jiang, H. Hao, Y. Chen, J. Dong, Y. Mao, Z. Zhang, R. Gao, W. Chen, R. Zhang. Matching the kinetics of natural enzymes with a single-atom iron nanozyme. *Nat. Catal.* **2021**, 4, 407.

[4] Y. Chen, B. Jiang, H. Hao, H. Li, C. Qiu, X. Liang, Q. Qu, Z. Zhang, R. Gao, D. Duan, S. Ji, D. Wang, M. Liang. Atomic-Level Regulation of Cobalt Single-Atom Nanozymes: Engineering High-Efficiency Catalase Mimics. *Angew. Chem.* **2023**, 62 (19), e202301879.

[5] R. Niu, Y. Liu, B. Xu, R. Deng, S. Zhou, Y. Cao, W. Li, H. Zhang, H. Zheng, S. Song, Y. Wang, H. Zhang. Programmed targeting pyruvate metabolism therapy amplified single‐atom nanozyme‐activated pyroptosis for immunotherapy. *Adv. Mater.* **2024**, 36, 2312124.

[6] S. Zhong, C. Xiong, Y. Zhao, S. Yao, Q. Hu, S. Wang, Q. Zhao, L. Li. Self‐driven electricity modulates d‐band electrons of copper single‐atom nanozyme for boosting cancer therapy. *Adv. Funct. Mater.* **2023**, 33, 2305625.

[7] D. Wang, J. Wang, X. J. Gao, H. Ding, M. Yang, Z. He, J. Xie, Z. Zhang, H. Huang, G. Nie. Employing noble metal–porphyrins to engineer robust and highly active single‐atom nanozymes for targeted catalytic therapy in nasopharyngeal carcinoma. *Adv. Mater.* **2024**, 36, 2310033.

[8] X. Lu, S. Gao, H. Lin, L. Yu, Y. Han, P. Zhu, W. Bao, H. Yao, Y. Chen, J. Shi. Bioinspired copper single‐atom catalysts for tumor parallel catalytic therapy. *Adv. Mater.* **2020**, 32, 2002246.

[9] M. Wang, C. Yang, M. Chang, Y. Xie, G. Zhu, Y. Qian, P. Zheng, Q. Sun, J. Lin, C. Li. Single-atom nanozymes based nanobee vehicle for autophagy inhibition-enhanced synergistic cancer therapy. *Nano Today* **2023**, 52, 101981.

[10] Y. Liu, B. Wang, J. Zhu, X. Xu, B. Zhou, Y. Yang. Single‐Atom Nanozyme with Asymmetric Electron Distribution for Tumor Catalytic Therapy by Disrupting Tumor Redox and Energy Metabolism Homeostasis. *Adv. Mater.* **2023**, 35, 2208512.

[11] J. Liang, B. Johannessen, Z. Wu, R. F. Webster, J. Yong, M. Y. B. Zulkifli, J. S. Harbort, Y. R. Cheok, H. Wen, Z. Ao. [B. Kong](https://advanced.onlinelibrary.wiley.com/authored-by/Kong/Biao), [S. Chang](https://advanced.onlinelibrary.wiley.com/authored-by/Chang/Shery+L.+Y.), [J. Scott](https://advanced.onlinelibrary.wiley.com/authored-by/Scott/Jason), [K. Liang](https://advanced.onlinelibrary.wiley.com/authored-by/Liang/Kang). Regulating the Coordination Environment of Mesopore-Confined Single Atoms from Metalloprotein-MOFs for Highly Efficient Biocatalysis. *Adv. Mater.* **2022**, *34*, 2205674.

[12] X. Wang, Q. Chen, Y. Zhu, K. Wang, Y. Chang, X. Wu, W. Bao, T. Cao, H. Chen, Y. Zhang. Destroying pathogen-tumor symbionts synergizing with catalytic therapy of colorectal cancer by biomimetic protein-supported single-atom nanozyme. *Signal Transduct. Target. Ther.* **2023**, 8, 277.
